# Probing the role of the vestibular system in motivation and reward-based attention

**DOI:** 10.1016/j.cortex.2018.02.009

**Published:** 2018-06

**Authors:** Elvio Blini, Caroline Tilikete, Alessandro Farnè, Fadila Hadj-Bouziane

**Affiliations:** a Integrative Multisensory Perception Action & Cognition Team (ImpAct), INSERM U1028, CNRS UMR5292, Lyon Neuroscience Research Center (CRNL), Lyon, France; b University of Lyon 1, Lyon, France; c Hospices Civils de Lyon, Neuro-Ophthalmology and Neurocognition, Hôpital Neurologique Pierre Wertheimer, Bron, France; d Hospices Civils de Lyon, Neuro-Immersion & Mouvement et Handicap, Lyon, France

**Keywords:** Attentional capture, Reward, Motivation, Spatial attention, Brain stimulation, Vestibular stimulation, Galvanic vestibular stimulation, Anterior cingulate cortex, Addiction

## Abstract

The vestibular system has widespread connections in the central nervous system. Several activation loci following vestibular stimulations have been notably reported in deep, limbic areas that are otherwise difficult to reach and modulate in healthy subjects. Following preliminary evidence, suggesting that such stimulations might affect mood and affective processing, we wondered whether the vestibular system is also involved in motivation. Evolutionary accounts suggest that visuo-vestibular mismatches might have a role in preventing the search for and exploitation of goods that previously resulted in aversive reactions, as they would be a fine warning signal which follows the contact with or ingestion of noxious neurotoxins. The first question was thus whether vestibular stimulation alters sensitivity to reward. Secondly, we sought to assess whether attention is allocated in space differently when cued by highly motivational stimuli, and if this interplay is further modulated by the vestibular system. In order to evaluate both motivational and attentional assets, we administered a Posner-like cueing task to 30 healthy subjects concurrently receiving sham or galvanic vestibular stimulation (GVS; Left-Anodal and Right-Anodal configurations). The participants had to discriminate targets appearing in either exogenously cued or uncued locations (50% validity); cues predicted the amount of points (0, 2, or 10) and thus money that they could earn for a correct response. The results highlight a robust inhibition of return (IOR) (faster responses for invalidly-cued targets) which was not modulated by different levels of reward or GVS. Across all stimulation sessions, rewards exerted a powerful beneficial effect over performance: reaction times were faster when rewards were at stake. However, this effect was largest in sham, but greatly reduced in GVS conditions, most notably with the Right-Anodal configuration. This is the first evidence for a decreased sensitivity to rewards causally induced by a perturbation of the vestibular system. While future studies will shed light on its neural underpinnings and clinical implications, here we argue that GVS could be a safe and promising way to enrich our understanding of reward processes and eventually tackle the management of patients with aberrant sensitivity to rewards.

## 1 Introduction

Dr. Brown is attending a tedious talk, stuck in the centre of a crowded theatre. He has just arrived in town after a long and difficult journey that made him miss lunch. Now he's so famished he could “eat an entire horse”. Meanwhile, people in charge of the catering are finally finishing preparing the generous buffet, and long tables full of delicacies are placed along the theatre's sidewalls. In this scenario, Dr. Brown has a very well-defined “priority map” (Serences, 2008), and while he is trying to be attentive to the speaker he also can't help peeking at those tasty sandwiches. What happens in his brain while the food exerts such a magnetic attraction on him, grabbing his eyes? This is both an intriguing and fundamental question, if we consider that the extreme deviation from this widely spread behaviour – i.e., aberrant and excessive attraction for intrinsically rewarding distracters – seems to be a recurrent feature in a variety of addiction disorders (Field, Munafò, & Franken, 2009, for meta-analysis).

To understand the impact reward exerts on attentional capture (AC) in a laboratory setting, several studies have successfully focused on monetary reward (a secondary need) because it's easier to manipulate parametrically and does not depend on transient states. Both immediate and long-term effects of monetary reward have been demonstrated in a variety of tasks (Anderson and Yantis, 2012, Anderson et al., 2011, Camara et al., 2013, Chelazzi et al., 2013, Della Libera and Chelazzi, 2006, Engelmann and Pessoa, 2007 Hickey et al., 2010, Munneke et al., 2015, Theeuwes and Belopolsky, 2012). For instance, in visual search tasks, general performance (Anderson et al., 2011, Chelazzi et al., 2014) and oculomotor behaviour (Bucker et al., 2015, Camara et al., 2013, Theeuwes and Belopolsky, 2012) can be biased by task-irrelevant distracters that were previously rewarded.

A wide network of interacting regions and chemicals (Grabenhorst & Rolls, 2011), every node being in charge of different cognitive operations, has been ascribed to the reward processing circuit in the brain. This network includes, among others, the anterior cingulate cortex (aCC) (Silvetti, Alexander, Verguts, & Brown, 2014). From an anatomical point of view, the aCC is an important hub linking areas in charge of evaluating reward desirability (e.g., orbitofrontal cortex, amygdala) and areas devoted to attentional selection and motor responses (e.g., parietal areas) (Amiez et al., 2006, van den Heuvel and Sporns, 2013; Lavin et al., 2013, Morecraft et al., 2012). It has a well-established role in monitoring outcomes and selecting the most appropriate and rewarding choice for future events (Amiez et al., 2006, Botvinick et al., 2004, Bush et al., 2002, Hadland et al., 2003, Rushworth et al., 2004) and reward-based learning (Kennerley et al., 2006, Lecce et al., 2015). Importantly, it also appears involved in the allocation of attentional resources towards rewarding stimuli in space (Hickey et al., 2010, Lecce et al., 2015, Mesulam, 1999). However, the aCC is unlikely to be the sole substrate of the interplay between motivation and attention, and some of the aforementioned features are also shared by other key areas (Engelmann, Damaraju, Padmala, & Pessoa, 2009) such as the temporo-parietal junction (TPJ) (Bisley and Goldberg, 2010, Mohanty et al., 2008), and the basal ganglia (Anderson, Laurent, & Yantis, 2014).

Interestingly, activation of all these structures has often been reported following non-invasive brain stimulation techniques such as caloric vestibular stimulation in PET (Bottini et al., 1994, Bottini et al., 2001) and fMRI studies (Fasold et al., 2002, Suzuki et al., 2001), as well as following galvanic vestibular stimulation (GVS) (Bense et al., 2001, Cyran et al., 2015, Lobel et al., 1998, Stephan et al., 2005). The latter technique consists of peripheral stimulation of the vestibular nerve (see Curthoys & MacDougall, 2012, for debate) through the application of small intensity currents over the mastoid bones (Fitzpatrick and Day, 2004, Utz et al., 2010). It has risen in popularity in recent years and proved to be a safe and promising tool for providing causal links between brain areas and behavioural performance (Been, Ngo, Miller, & Fitzgerald, 2007), with interesting applications for clinical cases. It also offers a unique way to modulate the activity of deep brain structures. Indeed, vestibular input is centrally represented in an extended network of multisensory areas (Guldin & Grüsser, 1998), including areas nearby TPJ, the operculo-insular/retroinsular cortex (Dieterich & Brandt, 2008, for a review; Lopez, Blanke, & Mast, 2012, and zu Eulenburg, Caspers, Roski, & Eickhoff, 2012, for meta-analyses), and deep and limbic cortices, including cingulate areas (Guldin and Grüsser, 1998, Guldin et al., 1992). Patients with vestibular neuritis exhibit an altered pattern of brain activations and deactivations similar to that observed following GVS (Alessandrini et al., 2013, Becker-Bense et al., 2013, Bense et al., 2004, Dieterich and Brandt, 2008). In such clinical population, aCC is part of a large network whose activity is altered with respect to healthy participants (Helmchen, Ye, Sprenger, & Münte, 2013), perhaps with some role in explaining the high comorbidity with psychiatric symptoms of anxiety and depression (Gurvich et al., 2013, Smith and Zheng, 2013).

Much of the interest around vestibular stimulation techniques arises from their known effectiveness in modulating spatial biases. For instance, they affect the setting of basic spatial coordinates, e.g., the subjective perception of “straight ahead” (Karnath, Sievering, & Fetter, 1994), of verticality (Mars, Popov, & Vercher, 2001), or the midpoint of a visual line (Ferrè, Longo, Fiori, & Haggard, 2013). While they do not seem to affect covert spatial attention (Rorden, Karnath, & Driver, 2001), vestibular stimulation nevertheless ameliorates the clinical manifestations of spatial neglect (Cappa et al., 1987, Rubens, 1985), possibly following parietal activations. By contrast, few studies have suggested that vestibular stimulation has the potential to also reach areas implicated in mood, affective processing, and motivation (Carmona et al., 2009, Mast et al., 2014, Preuss et al., 2014a, Preuss et al., 2014b), and a few case reports testify their effectiveness on psychiatric disorders such as mania (Dodson, 2004, Levine et al., 2012, for a review see also; Lopez, 2016). Experimental evidence is still scarce, though. In one notable exception, Preuss et al., 2014a, Preuss et al., 2014b reported that CVS can modulate affective control in a Go/No-go task that exploited emotional images as visual stimuli. In their study, left-cold CVS concurrently decreased affective control (as assessed by means of the d' sensitivity index) for positive images and self-reported positive mood ratings. Brain activations following vestibular stimulations, thus, extend to several of the deep areas involved in the processing of rewards. Nausea itself, such that originating from visual-vestibular mismatches, has been interpreted in light of evolutionary theories, underlining its role in preventing the search for and exploitation of goods that previously resulted in aversive reactions (Treisman, 1977). Under this view, nausea has been proposed to participate in the process of aversive conditioning, causing sensitization to repulsive stimuli that induce such unpleasant interoceptive sensations (Treisman, 1977). Based on this converging evidence, the aim of this study was therefore to take advantage of GVS to test the hypothesis that the vestibular system is involved in the processing of motivational stimuli. Our first question was whether perturbing the vestibular system results in altered sensitivity to rewards. Our second question was then whether AC is further modulated by GVS.

We administered GVS to healthy subjects performing a task assessing the interplay between attentional and motivational cues (Bucker & Theeuwes, 2014). AC has been described in a number of different paradigms. For the sake of this study, we exploited a well-established discrimination task with lateralized exogenous cueing (Posner, Snyder, & Davidson, 1980). When cues appear in the same spatial position as the target to be discriminated (i.e., valid trial), a behavioural advantage (i.e., validity gain) is commonly observed (Posner et al., 1980). In a few circumstances (e.g., non-predictive exogenous cues, long time intervals between cue and target) validity gain turns into inhibition of return (IOR): performance is impaired for cued locations, possibly reflecting a mechanism to optimally explore the visual environment by avoiding previously attended locations (Chica, Martín-Arévalo, Botta, & Lupiáñez, 2014). The main advantage of these indices is their ability to assess purely attentional processes, setting apart other motor and perceptual factors (Posner et al., 1980). Besides spatial information, cues provided participants with motivationally-relevant information (i.e., the colour of the cue predicted the monetary gain for that trial). Any modulation of the validity effect as a function of the allotted reward, thus, was thought to inform about the interaction between attentional and motivational processes.

A few studies previously assessed the validity of such a paradigm. For example, Munneke et al. (2015) found that validity gain is enhanced when cues predict large, as compared to smaller rewards, suggesting AC. In their discrimination task, Munneke et al. (2015) attached reward information to exogenous, peripheral cues on a trial by trial basis. Other settings yielded partially inconsistent results (Baines et al., 2011, Bucker and Theeuwes, 2014, Engelmann and Pessoa, 2007 Small et al., 2005). The inconsistency might reflect the sensitivity of such an attentional paradigm to even small differences in the experimental setting (Chica et al., 2014). Yet, this task appeared to be an appropriate choice for at least two reasons: 1) it highlights the overall beneficial effect of reward on performance (i.e., not in interaction with spatial attention); 2) neuroimaging studies have found that activity in brain regions coding for spatial expectancy is modulated as a function of reward (Baines et al., 2011, Engelmann et al., 2009, Small et al., 2005).

With this study, we thus sought to assess the behavioural effects of rewards over spatial attention, with particular reference to the validity effect, and then test the concurrent impact of a vestibular stimulation. We assessed any modulation of the validity effect following rewarding cues and during GVS (i.e., the three-way GVS by Reward by Validity interaction) seeking for a signature of the role of the vestibular system. Although the observed brain activations following GVS might possibly suggest a boost of AC phenomena during its administration, it's unclear that such brain activation invariantly leads to the enhancement of a behavioural effect (i.e., inhibitions might be expected as well). To the best of our knowledge, to date there is no evidence on the effect of GVS on reward processes. Thus, no directional starting hypothesis was posed on the two-way (GVS by Reward) and three-way (GVS by Reward by Validity) interactions. The scope of our study was to test whether the vestibular system is implicated in the processing of reward. By attempting to answer the question “whether” instead of “how”, we engaged ourselves in making cautious post-hoc claims in the presence of an actual modulation. This study was meant to provide a first proof of principle on the role of the VS in the interplay between reward and attention, which could pave the way to future clinical and neuroimaging studies.

## 2 Material and methods

### 2.1 Participants

**Fig. 1:**
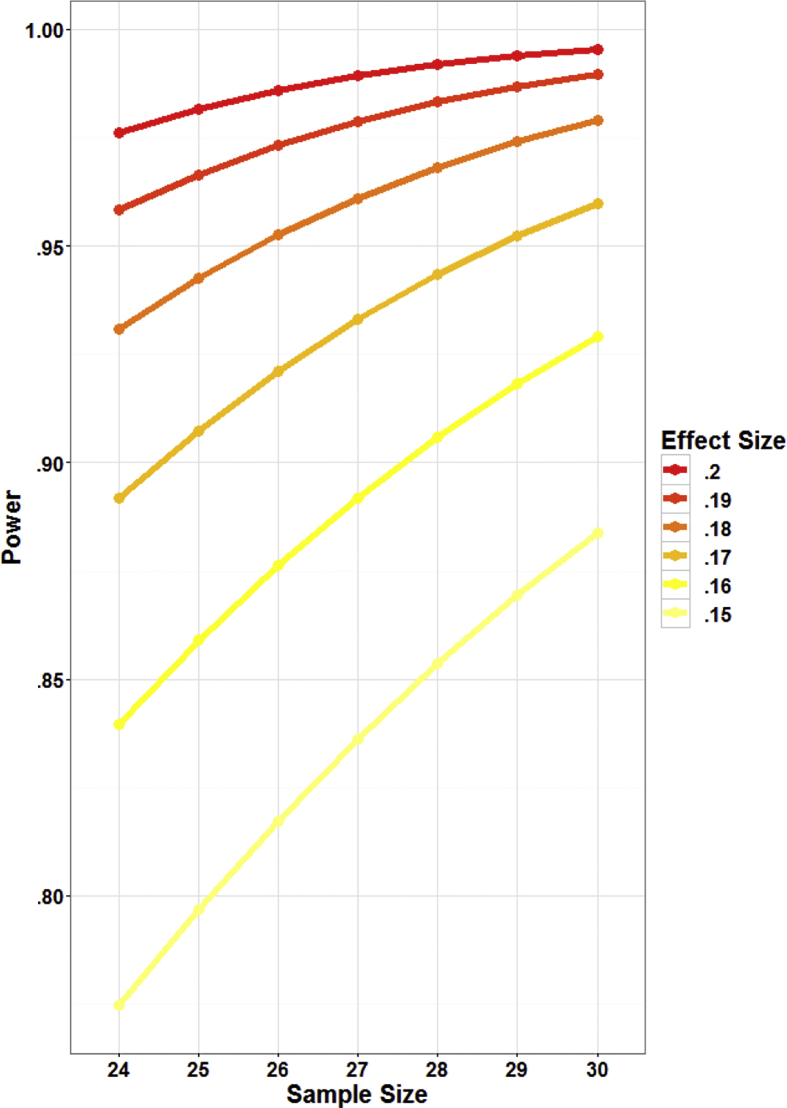
Nominal power is depicted as a function of arbitrary ranges of sample and effect sizes.

A tentative power analysis was performed through the G*Power software (Faul, Erdfelder, Lang, & Buchner, 2007). We computed a priori power for our ANOVA design (F tests family, repeated measures – within factors). We used, as input parameters, an effect size *f* = .17, alpha level = .05, and correlation among repeated measures = .5. We assumed no violations of sphericity, and thus did not apply any correction. Our design only considered 1 group of subjects with 18 repeated measures (GVS by Reward by Validity, 3 × 3 × 2). A power of 90% was found to be achievable at *N* = 25. To properly counterbalance all conditions in our design (see below in the methods) we needed a sample size with a multiple of 6 subjects (3 levels of GVS × 2), thus we back-computed achieved power for *N* = 24 (89.1% power) and *N* = 30 (95.9% power). Fig. 1 depicts the nominal power for a range of *f* values and different sample sizes (R script provided in the supplementary materials).

We decided to enrol a maximum of 30 participants, but a first interim analysis was planned at *N* = 24. Type 1 error correction for optional stopping was adopted (Lakens, 2014) in order to control for the cumulative Type 1 error rate (set to .05). Applying Pocock (1977) continuous boundaries for two sequential analyses not equally spaced is equal to adjust the alpha level to .043. Note that this new alpha threshold did not substantially impact the outcome of power analysis (a power of 90% is reached at *N* = 26). A Bayesian secondary analysis through Bayes Factors (BF) was envisaged to help in clarifying the extent to which the collected data were conclusive (Schönbrodt & Wagenmakers, 2017).

The first interim analysis (*N* = 24) found support for the two-way interaction GVS by Reward, but not for the three-way GVS by Reward by Validity. We therefore enrolled 30 healthy young participants (18–30 years, *M* = 22.9, SD = 3.1), 12 males and 18 females; they were all right-handed and had normal or corrected-to-normal vision. Prior to the experiment, they completed a standard safety questionnaire in order to minimize the occurrence of side effects and check for exclusion criteria. Exclusion criteria were the following:

• Left handedness;
• Colour blindness (due to the fact that reward information was conveyed via colours);
• History of epileptic seizures or close (first degree) familiarity with epilepsy;
• History of neurologic or psychiatric disorders (including recurrent migraine or headaches);
• History/presence of heart diseases;
• History of recurrent otitis media, vestibular disorders, or perforation of the tympanic membrane;
• Presence of metallic implants or metallic splinters in the body;
• Severe sleep deprivation during the last 24 h;
• Consumption of psychotropic drugs, substances, or alcohol during the last 24 h;
• Participation in other experiments involving stimulation techniques during the last week.

Only participants fulfilling the above criteria were officially recruited for this study (*N* = 32). One subject had to be discarded due to an error in the script running the main experimental task. Another one was included but did not attend all the planned sessions. Written informed consent was obtained from all participants. The study was approved by the relevant French Institution (Comité de Protection de Personnes, CPP, 2015-A00623-46, FEEDBACK protocol).

### 2.2 Behavioural tasks

Participants were tested in a dimly lit, quiet room. Their head was restrained by a chinrest, facing a 15 inches large screen at a distance of approximately 57 cm. The open-source software OpenSesame (Mathôt, Schreij, & Theeuwes, 2011, http://osdoc.cogsci.nl/) was used to display experimental stimuli on the screen and record the subjects' response. Participants provided responses by means of keyboard presses (on a standard QWERTY keyboard) using the index and middle fingers of their dominant hand.

Two tasks were administered during GVS. The main task evaluates attentional assets and their modulation according to the rewards at stake (attention and reward task – ART). This part was designed to last a maximum of 25 min. A control task (subjective visual vertical – SVV) was also administered before the main task. SVV requires rotating visual segments until they appear to be in a vertical position. It was meant to provide independent evidence of GVS effectiveness, given that displacements occur towards the site of labyrinthine dysfunction (Böhmer and Rickenmann, 1995, Vibert et al., 1999) or anodal GVS stimulation (Mars et al., 2001, Saj et al., 2006). It was therefore meant to provide evidence of a successful stimulation, and means to correlate the perceptual effects of GVS (effectiveness of the stimulation) with results obtained from the ART task. The scripts to run these tasks can be found in the supplementary materials.

Finally, a brief evaluation of subjective feelings and sensations experienced during each session was administered. It was meant to monitor participants' distress and task compliance across the different days of the experiment and GVS protocols.

#### 2.2.1 ART

**Fig. 2:**
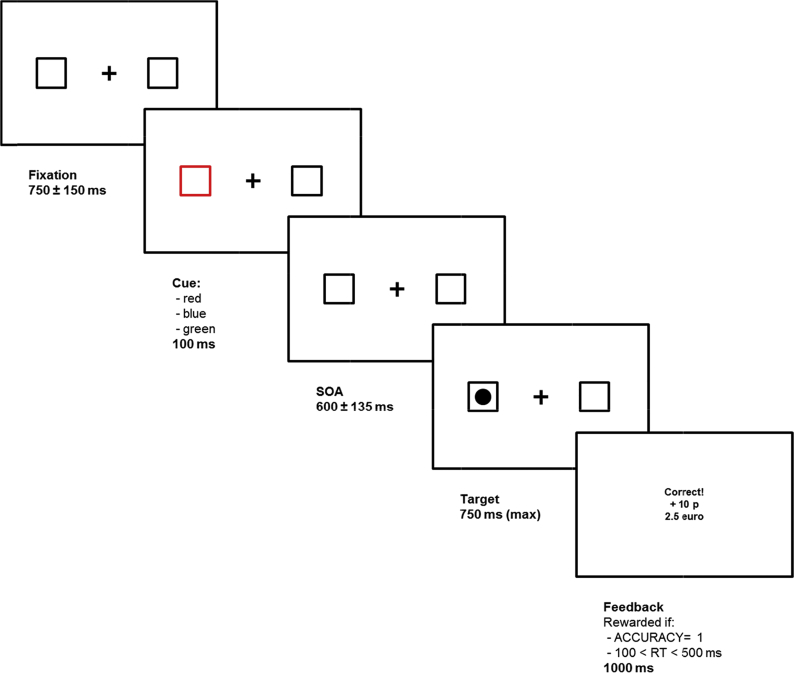
Graphical representation and time-course of a typical trial of the ART.

A schematic depiction of the sequence of the task is illustrated in Fig. 2. Experimental stimuli were white coloured and presented on a black background. Each trial started with a fixation cross (1.8 × 1.8°) appearing at the centre of the screen. Participants were instructed to maintain fixation and avoid eye movements throughout the session. Placeholders (3.5 × 3.5°) were presented at both sides of the screen, at a distance of 8 cm from the centre (8° of visual angle). The fixation phase lasted 750 msec (with a 150 msec uniform jitter). Then, one of the two boxes (the left and right with a probability of 50% each) changed colour (100 msec). Different colours, red, green or blue informed the subject about the possibility to receive a Reward of 0, 2, or 10 points in case of a correct response in the following phase. Colour-reward associations changed randomly for each participant. Then, placeholders returned to their default colour and were presented on screen for 600 msec (jitter: 135 msec; total Stimulus Onset Asynchrony, SOA: 700 msec). After this SOA elapsed, either a square or a circle (1.5 × 1.5°, filled-white) was randomly presented within one of the two placeholders. Target Side (left, right) was the same (valid) or different (invalid) to that of the cue in half of the trials, thus the peripheral exogenous cue was non-predictive (Validity 50%). Participants were informed that these contingencies were equiprobable. Such a procedure (e.g., a relatively long SOA, exogenous non-predictive cueing) is most commonly linked to IOR (Chica et al., 2014, Klein, 2000), which consists of an impaired performance for previously cued locations. IOR is thought to reflect a phenomenon that is complementary (yet distinct) to the classic validity gain occurring at short SOAs (Klein, 2000). Being defined in terms of validity costs, it still indexes the allocation of attention in space and was thus appropriate to respond our experimental questions (also see Bucker & Theeuwes, 2014).

The target remained on screen for a maximum of 750 msec or until a response was provided. Participants were required to press the button corresponding to the previously presented geometric shape (contingencies counterbalanced across participants). To ensure that discrimination was unconfounded by any response bias/motor preparation, the response dimension was orthogonal to stimuli lateralization (that is, the DOWN and UP arrows opposed to left/right stimuli presentation were used, see Spence & Driver, 1994). Reward was provided only in case of a response that was both accurate and given within a 100–500 msec time window, with the purpose of: i) discouraging anticipations, and ii) providing a challenging upper limit, hence promoting active efforts to achieve rewards (expected success rate: 85–90%). Feedback was eventually presented for 1000 msec. The feedback reported the outcome of the trial (correct, incorrect, slow, or fast response). Following each correct response, the amount of points earned was reported, together with the overall amount of euros gained until that moment.

The experiment consisted of 432 trials (Shape, Side, Validity, Reward = 2 × 2 × 2 × 3, 18 trials per cell). It started with a brief practice block composed of 18 randomly selected trials, performed before the onset of the stimulation. Then, three blocks of 144 trials each were administered, with breaks in between that summarised participants' performance. Each of the four experimental variables was equi-represented within each block and randomly selected. The whole experiment was designed to last about 25 min, to accommodate the maximum time limit for GVS administration (see below).

#### 2.2.2 SVV

One segment (17 cm, 2 mm wide; white-coloured, over a black background) was presented at the centre of the screen. Its starting orientation varied randomly between 1 and 20° from the geometric (objective) vertical, in both clockwise and counter-clockwise directions (counterbalanced). Participants were asked to align the segment along the vertical plane by manually rotating it clockwise or counter clockwise using the keyboard. Segments' orientation changed with steps of .1° (between −1.6 and 1.6° from the vertical) up to steps of .7° for more extreme responses (>19°). Participants were thus informed that a counting strategy was counter-productive, and asked to stress accuracy over speed.

A circular black panel covered the borders of the screen, to minimize the use of external anchoring points to perform the task. For the same reason, the experiment was performed in darkness. A total of 24 trials were given.

As dependent variable, the orientation of the SVV (in degrees) was stored. Positive values reflected a shift occurring clockwise (i.e., towards the right ear), while negative values reflected a counter-clockwise bias.

#### 2.2.3 Subjective experienced sensations

After each session, we asked participants questions exploring their subjective experience with the stimulation they received. We asked them to grade their illusion of body movement (left–right, up–down, forward–backward), of head tilting, and of the visual scene moving; their degree of nausea and vertigo; the feeling of some part of their body changing size; the amount of itching, burning, and overall distress due to the electrodes. Furthermore, three questions specifically concerned their feelings about the ART task: the amount of concentration devoted to the task, the amount of motivation to perform well, and the specific motivation prompted by the rewards at stake.

Each question was presented on a computer screen, above a horizontal visual line representing a scale continuum. Subjects graded their experience by means of mouse-clicks on the line. The standardized displacement from the objective centre of the segment (i.e., −100% for the leftmost end, 0% for the exact centre, +100% for the rightmost end) was stored as the dependent variable for each question.

### 2.3 GVS

GVS was delivered via a commercial, CE approved, stimulator (BrainStim, EMS, Bologna). The application of small current intensities over the mastoid bones is associated with the illusion of head and body movements towards the side of anodal stimulation, but induces very few adverse effects with stimulation up to 1.5 mA in both healthy and brain-damaged patients (Utz, Korluss, et al., 2011). A large study (*N* = 255) found that about 10% of subjects felt slight itching and/or tingling sensations below the electrodes, but no occurrence of seizures, vertigo or nausea was reported (Utz, Korluss, et al., 2011).

Electric current of 1 mA was administered continuously during each GVS session. A constant stimulation has been especially employed for rehabilitation purposes (Kerkhoff et al., 2011, Rorsman et al., 1999, Utz et al., 2011). We used spongy electrodes (14 cmˆ2 area) soaked with saline water and fixed in place with adhesive tape and a rubber band. Stimulation was delivered only after an initial impedance check, to minimize potentially painful sensations. Three configurations were adopted: Left- and Right-Anodal are considered active GVS conditions, inducing different (polarity dependent) effects. Electrodes were placed on mastoid processes symmetrically (that is, in the Left-Anodal montage the cathode was placed over the right mastoid bone, and vice versa for the Right-Anodal one). Left-Anodal stimulation activates mainly right hemisphere structures, whereas Right-Anodal activates comparatively more left hemisphere structures. A sham condition was also included, with electrodes placed symmetrically about 5 cm below the mastoids, above the neck, and distant from the trapezoidal muscles yielding proprioceptive signals (Lenggenhager, Lopez, & Blanke, 2007). The sham condition was included to control for unspecific factors of electrical stimulation (e.g., arousal, discomfort) that are known to have an important role in modulating performance in spatial tasks. The anode was placed in this case on the left side (Ferrè et al., 2013). Participants performed the behavioural tasks three times, on three different days, under each GVS condition (Left-Anodal, Right-Anodal and sham). The order of GVS type administration was counterbalanced across subjects. Within each session, active stimulation was delivered for a maximum of 30 min for safety reasons (Rossi, Hallett, Rossini, & Pascual-Leone, 2009).

Participants received a monetary compensation of 75 euros for their participation in the three sessions. They could receive an additional amount up to 25 euro(s)/session according to their performance in the ART task, proportionally to the amount of points gained (over 1728 available points, hence ∼.015 euro per point).

### 2.4 Analyses

#### 2.4.1 Data pre-processing

Data, excluding practice trials, were analysed with the open-source software R (The R Core Team, 2013).

We adopted the following criteria to discard and replace subjects:

• *Lack of participation in at least one of the three planned sessions*. Participants might not attend all the planned sessions, for reasons that might or might not depend on the experimental setting itself (e.g., participants who experience particularly distressing sensations following one GVS session). Technical issues might arise in case of very high impedance on participants' skins, and prevent GVS administration. One subject had to be replaced because he could not attend the second and third sessions for personal reasons (not related to GVS-induced distress).
• *Low performance (ART task)*. A subject could be replaced if the overall proportion of responses that was both correct and provided within 500 msec was below .6. The threshold was set to .3 per cell when assessing performance in each Validity by Reward by GVS occurrence. All the participants outperformed this criterion.
• *Outlier classification (ART task)*. A subject could be replaced if one of the cell means within the GVS by Reward by Validity interaction exceeded ±3 standard deviations from the subject's mean. There were no outliers.

We decided to analyse accuracy only if the group mean was less than 95%, and if fewer than half of the subjects presented more than 95% success rate (to avoid ceiling effects). We decided to assess RTs only for responses that were both accurate and given within the 100–500 msec time window.

#### 2.4.2 Statistical analyses

##### 2.4.2.1 ART

Results were analysed through *mixed-effects multiple regression models* (Baayen, Davidson, & Bates, 2008) using the lme4 package for R (Bates, Maechler, Bolker, & Walker, 2014). Models had a logistic link-function, appropriate for binary variables, when assessing accuracy. As a first step, we defined a model containing the most appropriate random effects. Theoretically, models including all the possible random effects (justified by experimental design) would lead to the most informative solution. However, complicated matrices often result in convergence problems in the face of a negligible increase in the amount of information provided or even overfitting (Bates, Kliegl, Vasishth, & Baayen, 2015). Random effects were thus introduced sequentially, their effect on model fit assessed through likelihood ratio tests (LRT): residuals of each model were compared, and the one with significantly lower deviance as assessed by a chi-squared test was chosen.

*Random effects selection*. Restricted maximum likelihood was used to test random effects. We started with a random intercept for Subject only. Then, we tested random slopes, in the following order:

• GVS (3 levels: sham, Left-Anodal, Right-Anodal);
• Reward (3 levels: no reward, mid reward, high reward);
• Validity (2 levels: valid, invalid);
• Target Side (2 levels: left, right);
• Block (3 levels: block 1, 2, or 3);

Additionally, random slopes for Shape were evaluated (but not the fixed effect). Note that Shape was confounded with response effector (e.g., square-index *vs* circle-middle finger), although the contingency was counterbalanced across subjects. The Block factor was introduced to assess any modulation of rewards effectiveness over time (e.g., at the beginning or at the end of the stimulation).

Random slopes were tested sequentially, meaning that each was retained in the reference model as soon as an LRT supported its role. For example, in case of a significant increased fitting for the random slope of GVS, the random slope of Reward would have been evaluated against a model including it. Once all six random slopes were evaluated, we tested random slopes for the interactions. Only interactions of factors that were selected at the previous step were tested. For example, if both (and only) the random slopes for GVS and Reward were found to be significant, only the two-way GVS by Reward would have been further tested through LRT. The reason for this restriction was to have models fulfilling the marginality principle (each higher order term included only in presence of its lower level terms) and limit problems in model convergence.

*Fixed effect testing*. Maximum likelihood was used to test fixed effects, using models with the final random effects structure. We adopted type 2 sequential tests for the factors listed in the previous paragraph. In this approach, each effect or interaction is compared, through LRT, to a restricted model that excludes the effect itself. For example, a two-way interaction is assessed by comparing the model including the interaction (and relative main effects, to fulfil the marginality principle) against a model that only included the two main effects themselves. Differently from type 3 tests, type 2 tests are not conditional to other covariates being included in the model (thus, results do not depend on the presence/absence of “moderating” factors). The LRT outcome was then our main inferential criterion. We used *p*-values adjustments for multiple testing (see below) and an alpha level of .043 (see section 2.1).

For a Bayesian counterpart, Bayes Factors (BF) were obtained through objective Cauchy-distributed priors (i.e., assuming that 50% of observed normalized effect sizes might fall in the −.7 to +.7 interval; Rouder, Morey, Speckman, & Province, 2012) using the BayesFactor package for R (Morey, Rouder, & Jamil, 2015), in the context of an ANOVA design. A Bayes Factor larger than 1 supports the alternative hypothesis, while a BF smaller than 1 supports H0; it is best used to grade the strength of evidence for one model over another.

*Procedure for dealing with non-convergence*. Despite the selection procedure mentioned above, convergence problems could still arise during the testing of both random and fixed effects. In order to minimize this problem, we exploited several different optimization algorithms and increased the maximum number of iterations up to 10^20^. If this was found to be insufficient, we planned to further simplify the matrix of random effects, starting by dropping the correlation term between random slopes and intercept. In case this was not sufficient, we planned to proceed in dropping one by one higher-order random slopes and all other terms, following the reverse order with respect to the one adopted in the selection phase.

*Corrections for multiple testing*. Four tests were the main focus of our proposal:

• The main effect of Reward;
• The Reward by Validity interaction;
• The GVS by Reward interaction;
• The three-way interaction: GVS by Reward by Validity.

Thus, these four tests formed our family of tests of interest. All other factors and interactions could cast interesting observations, but were not the focus of this work and thus formed a separate family of tests. For both families independently, we applied *p*-value adjustments for false discovery rate (FDR; Benjamini & Hochberg, 1995).

##### 2.4.2.2 SVV

Procedures were the same as above, but data were trimmed, for each subject, at ±2.5 standard deviations from the subject-specific mean.

The fixed (and random) factors that were introduced and tested are:

• GVS (3 levels: sham, Left-Anodal, Right-Anodal);
• Starting Side (2 levels: clockwise, counter clockwise).

We expected a large, yet not of interest, effect for Starting Side – lines originally displayed as tilted clockwise associated with a clockwise response bias (similarly to what is typically found for line bisection tasks, Jewell & McCourt, 2000). *p*-Values for GVS and the two-way GVS by Starting Side were adjusted for FDR and evaluated against an alpha level of .043.

##### 2.4.2.3 Subjective experienced sensations

The SVV task represents the elective control for the effectiveness of GVS. In addition, we evaluated subjective experience following GVS (e.g., probing subjective feeling of distress or motivation). We did not pre-register any specific analysis for these evaluations. This part was exploratory and capitalised on visually- and data-driven procedures. Any inferential technique deemed relevant for the scope of this study exploited Bayes Factors with objective Cauchy-distributed priors. Given the explorative aspect of this evaluation, we set a more conservative threshold (BF_10_ ≥ 10), with respect to the previous two tasks.

#### 2.4.3 Outcome quality controls

##### 2.4.3.1 Effect of rewards

We assumed that points, because they were converted into monetary rewards at the end of the experiment, could provide participants enhanced motivation to produce a valid response. This was assessed through the main effect of Reward in the ART task (a significant LRT). As a second point, at least one coefficient in the model had to indicate an improved performance for the maximum reward with respect to the null reward. Particular expectations were posed on reaction times, which were expected to be faster with high rewards and possibly with higher accuracy for high rewards. In case of speed-accuracy trade-offs, we deemed this criterion as fulfilled if the proportion of valid trials (that is, both correct and timely) was higher for the maximum reward.

The aim of this outcome quality control was to ensure that rewards (2 points ≈ .03 euro, or 10 points ≈ .15 euro) modulated participants' performance, and thus that any other interaction (e.g., Reward by Validity) could be safely interpretable as induced by an altered motivational state.

##### 2.4.3.2 Effect of GVS on the SVV

GVS was expected to tilt the SVV towards the site of anodal stimulation (Lenggenhager et al., 2007, Mars et al., 2001). We therefore predicted that the LRT for GVS would have been significant. We decided then to proceed in assessing model coefficients. No prediction was made for specific outcome configurations (e.g., Right-Anodal > sham, Right-Anodal > Left-Anodal, sham > Left-Anodal). In principle a gradient could have been observed, though we adapted a clinical test to a (quick) computer-based testing and minor deviations along this pattern could have been tolerated. At least one coefficient, however, had to suggest that the SVV was tilted towards the anodal site (e.g., sham < Right-Anodal).

An effect on the SVV confirms the effectiveness of GVS and thus the activation of the vestibular system, and specifically its otolithic component.

##### 2.4.3.3 Effect of reward over spatial attention

Evidence for a two-way interaction Reward by Validity (a significant LRT) was expected to greatly ease the interpretation of any modulation given by GVS. The validity effect had to be either enhanced (Bucker and Theeuwes, 2014, Munneke et al., 2015) or abolished (Engelmann et al., 2009) by rewards. The interaction and follow-up comparisons had to suggest that spatial attention was more strongly attracted by exogenous cues in case rewards were attached to them, resulting in a modulation of validity gain/IOR (e.g., Munneke et al., 2015). Yet, this criterion was not meant to be necessarily fatal if not met. We thought in principle possible that GVS might enhance a subtle effect, leading to a meaningful modulation, although, if this was the case, caution was warranted in discussing results. Furthermore, our second question, i.e., whether the vestibular system is involved in the processing of motivational stimuli (that is the GVS by Reward interaction), was independent of this quality check.

## 3 Results

Raw data, the full analysis pipeline and scripts, and additional graphical depictions are available in the supplementary materials and at the following link: https://data.mendeley.com/datasets/b5grw3pz3r/draft?a=a7556d48-1f30-4b2c-a9e4-2031012e3541. As previously stated, we eventually enrolled 30 participants because the first interim analysis (*N* = 24) only provided evidence for the GVS by Reward interaction (but not for the other key tests of this work, e.g., the three way GVS by Reward by Validity). The following packages for R greatly eased our work: afex (Singmann, Bolker, Westfall, & Aust, 2015); BayesFactor (Morey, Rouder, & Jamil, 2015); lme4 (Bates et al., 2014); tidyverse (Wickham, 2017), and notably ggplot2 (Wickham, 2016).

### 3.1 Pre-registered: SVV

We discarded a small (<.01) percentage of outliers. The selection procedure described above led us to select the full random effects model, i.e., which included random slopes for GVS and Starting Side as well as their interaction.

**Fig. 3:**
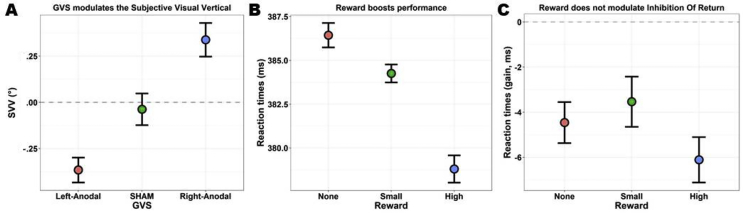
Quality checks. A) GVS modulated the subjective visual vertical. The visual lines were tilted counter-clockwise with Left-Anodal GVS, clockwise for Right-Anodal. This allows us to conclude that the vestibular system was effectively perturbed by our stimulation. B) Rewards had a powerful effect on performance in the ART task. Reaction times decreased as a function of reward, demonstrating that participants' motivation was effectively manipulated by our points-system. C) The inhibition of return (IOR) consists in impaired performance for cued locations (shown as negative validity gains), and it was not affected by the allotted reward. Visual inspection may suggest an enhancement of IOR with high rewards, but statistical tests failed to reach significance. The gain (*y*-axis) reflects the difference in RTs between valid and invalid trials. All plots depict the mean and within-subjects SEM (Morey, 2008). Please refer to the supplementary materials for more information and graphical depictions.

As predicted, Starting Side was significant (*χ*^2^_(1)_ = 19.71, *p* < .001, BF > 1000). The SVV was tilted clockwise when lines originally appeared on screen tilted clockwise, and it was tilted counter-clockwise when trials started with a line tilted in the counter-clockwise direction. GVS was also significant (*χ*^2^_(2)_ = 30.19, *p*_*fdr*_ < .001, BF > 1000). All post hoc contrasts between the three GVS conditions were significant (all *|z|* > 2.86, all *p*_*fdr*_ < .004) indicating the existence of a gradient Left-Anodal < sham < Right-Anodal supporting the claim that the SVV is tilted towards the position of the site of anodal stimulation and the effectiveness of GVS in the present study. Results are depicted in Fig. 3 and detailed in Supplementary 1. Finally, GVS and Starting Side did not interact (*χ*^2^_(2)_ = .13, *p*_*fdr*_ = .94, BF = .1).

### 3.2 Post hoc: evaluation of GVS effects

We evaluated vestibular-specific and unspecific effects of GVS after each session through a questionnaire. Among the 15 follow-up questions, which can be found together with details in Supplementary 2, only one (barely) reached significance in an ANOVA (main effect of GVS: *F*_(2, 58)_ = 3.24, *p* = .046). At the sentence “I felt my body rotating” subjects more often responded “clockwise” for Right-Anodal, counter-clockwise for Left-Anodal. This was the only question for which the Bayes Factor exceeded 1 (BF = 1.6), yet still remaining below our predefined threshold of 10. For all remaining questions, the Bayes Factor approach supported, with variable degree, the absence of a GVS effect (and no other ANOVA was found to be significant). Thus, GVS conditions did not differ from sham in terms of subjective sensations/unspecific effects, ruling out the possibility that general discomfort, nausea, vertigo, or other causes of distress (e.g., itching/burning) might have played a major role in driving the effects observed in the ART task.

### 3.3 Pre-registered: ART

Subjects were overall very accurate (94.2%). Accuracy was higher than 95% for 13 subjects (43.3%). Our criterion to proceed with analyses was thus fulfilled, yet with performance at ceiling for a few cells and subjects. We eliminated convergence problems only with the simplest matrix of random effects (i.e., without random slopes). The only effect that survived FDR correction was that of Side (*χ*^2^_(1)_ = 17.7, *p*_*fdr*_ < .001): responses to right-sided targets were more accurate than those to left-sided ones (*|z|* = 4.34, *p* < .001). More details are available in Supplementary 4.

We then moved to assessing RTs. The selection procedure for random effects led us to select the random slopes for GVS, Reward, and Validity, which were thus included in all models and LRT for fixed effects. The results of fixed effects testing are reported in Table 1 and detailed in Supplementary 3.

**Table 1:** Results of fixed effects testing for reaction times in the ART task. The table reports the statistics of LRT for all fixed effects in our design. The four tests of interest for this study are reported over a grey background; these were corrected for false discovery rate separately from the other factors. The tests surviving corrections and significant at a threshold of .043 (alpha level corrected for sequential analyses) appear in bold font.

Test	Chisq	Chi Df	Pr (>Chisq)	Pr (fdr)	Sig (*p* < .043)	ηp2
GVS	1.9	2	.387	.631		.029
**Reward**	**29.25**	**2**	**<.001**	**<.001**	***	**.539**
**Validity**	**9.23**	**1**	**.002**	**.016**	*	**.262**
**Side**	**55.24**	**1**	**<.001**	**<.001**	***	**.251**
**Block**	**12.63**	**2**	**.002**	**.016**	*	**.042**
**GVS:Reward**	**19.11**	**4**	**<.001**	**.001**	**	**.095**
GVS:Validity	2.18	2	.336	.631		.019
Reward:Validity	3.69	2	.158	.211		.042
GVS:Side	3.37	2	.185	.417		.049
Reward:Side	1.05	2	.591	.76		.011
Validity:Side	5.03	1	.025	.084		.072
**GVS:Block**	**23.63**	**4**	**<.001**	**.001**	**	**.066**
Reward:Block	12.71	4	.013	.057		.081
**Validity:Block**	**9.8**	**2**	**.007**	**.04**	*	**.093**
Side:Block	.27	2	.873	.99		.003
GVS:Reward:Validity	3.98	4	.409	.409		.04
GVS:Reward:Side	3.73	4	.444	.631		.032
GVS:Validity:Side	1.7	2	.427	.631		.03
Reward:Validity:Side	4.82	2	.09	.243		.068
GVS:Reward:Block	18.43	8	.018	.07		.077
GVS:Validity:Block	1.63	4	.803	.986		.01
Reward:Validity:Block	0.3	4	.99	.99		.005
GVS:Side:Block	4.25	4	.373	.631		.031
Reward:Side:Block	5.44	4	.245	.508		.035
Validity:Side:Block	.14	2	.93	.99		.004
GVS:Reward:Validity:Side	7.67	4	0.1	.256		.08
GVS:Reward:Validity:Block	3.35	8	.91	.99		.014
GVS:Reward:Side:Block	8.06	8	.428	.631		.046
GVS:Validity:Side:Block	.42	4	.981	.99		.006
Reward:Validity:Side:Block	8.18	4	.085	.243		.065
GVS:Reward:Validity:Side:Block	7.06	8	.53	.715		.036

#### 3.3.1 Pre-registered: tests of interest

**Fig. 4:**
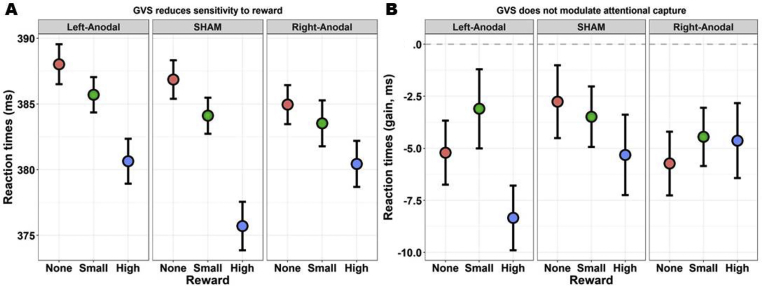
Tests of interest. A) If rewards generally resulted in a performance boost, especially for the maximum of 10 points, this effect was significantly reduced in the Right-Anodal GVS condition. A similar trend was observed for Left-Anodal, but this effect did not survive corrections. We conclude that Right-Anodal GVS reduces sensitivity to rewards. B) On the other hand, despite the visual trend, there was no three-way interaction between GVS, Reward, and Validity. Our ART setting induced IOR, as seen by negative validity gains. The IOR visually appears modulated by rewards – high rewards enhancing it – in the sham and Left-Anodal condition, though not in the Right-Anodal, yet the test did not reach significance. All plots depict the mean and within-subjects SEM (Morey, 2008). Please refer to Supplementary 3 for more information and graphical depictions.

The four tests of interest are highlighted in grey in Table 1. First, the main effect of Reward was significant (*χ*^2^_(2)_ = 29.25, *p*_*fdr*_ < .001). Reaction times were modulated by the amount of points that were at stake, being slowest when no points were given, faster with a small reward of 2 points, and fastest with the maximum reward (10 points). All the contrasts reached significance at the FDR-corrected threshold (all |*z*| > 2.77, all *p*_fdr_ < .006). Results are depicted in Fig. 3. Second, the effect of Reward was not modulated by Validity (*χ*^2^_(2)_ = 3.69, *p*_*fdr*_ = .211; also shown in Fig. 3). Thus, despite the visual trend, IOR was not significantly modulated by large rewards. Third, while IOR also visually appears enhanced for high rewards in sham and Left-Anodal conditions, but not in the Right-Anodal condition, the three-way interaction GVS by Reward by Validity was not significant (*χ*^2^_(4)_ = 3.99, *p*_*fdr*_ = .409). Results are depicted in Fig. 4. Finally, the last key test, namely the GVS by Reward interaction, was significant (*χ*^2^_(4)_ = 19.11, *p*_*fdr*_ = .001; Fig. 4). We thus compared the magnitude of motivation-induced boosts of performance (i.e., the effect of Reward) across GVS conditions. We found that the benefits of high rewards (compared to both no rewards and small rewards) were greatly reduced in the Right-Anodal condition with respect to sham (all *|z|* > 3.37, all *p*_fdr_ < .004). There was also a similar trend for the Left-Anodal condition in which, as compared to sham, the benefits of high rewards were reduced, but the contrasts did not survive FDR correction (all *|z|* < 2.22, all *p*_fdr_ > .079). Complementary analyses probing the robustness of this test will be presented below (section 3.4).

**Fig. 5:**
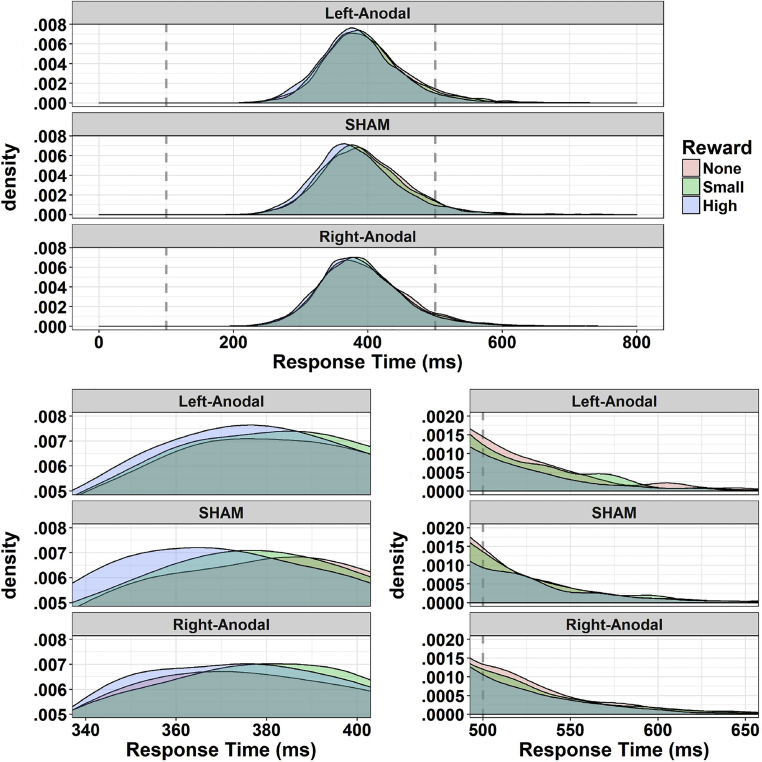
Distribution of RTs in the three GVS conditions. The distribution (density) of the reaction times for correct responses, as a function of Reward and GVS, is depicted. Reward was associated with three clearly separated RTs distributions in the sham condition, but with comparatively more overlap in the two GVS conditions. This is best shown in the bottom left panel zoom. There was a tendency, though not significant, for the reverse effect in the long tails of the distribution (bottom right panel), thus for values that were not included in other analyses. The residual effects of rewards appear to persist longer for the two GVS conditions, less so in the sham condition. This suggests an important role of the time-pressure given to participants (500 msec time limit).

**Fig. 6:**
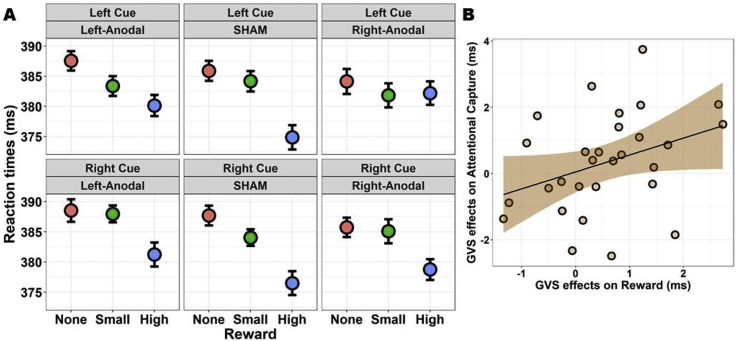
Exploratory analyses. A) An exploratory ANOVA using the Side of the *cue* instead of that of the target revealed some evidence for a largest reduction of sensitivity to rewards in the Right-Anodal GVS following left-sided cues. Note that this effect did not survive corrections. B) There was a non-significant (*p* = .064) positive trend between the reduction of sensitivity to rewards and the reduction of attentional capture by high rewards induced by GVS (*r* = .35). On the *x*-axis, we computed the amount of reduction of sensitivity to rewards induced by the Right-Anodal condition relative to the sham condition using the difference between slope coefficients. On the *y*-axis, similarly, we computed the amount of reduction of AC induced by the Right-Anodal condition relative to the sham condition. Note that one very influential observation was omitted in this plot (see the main text).

#### 3.3.2 Pre-registered: other findings

Table 1 also includes results of tests of secondary interest for our study. The main effect of Validity, for example, was significant (*χ*^2^_(1)_ = 9.23, *p*_*fdr*_ = .016), indicating that Invalid trials were generally faster than Valid ones (*|z|* = 3.25, *p* = .001), resulting in the presence of IOR. This effect was modulated by Block (*χ*^2^_(2)_ = 9.8, *p*_*fdr*_ = .04): IOR grew over time, being larger for the last block with respect to the first one (*|z|* = 3.04, *p*_fdr_ = .007). The differences were less pronounced between the first and second block (*|z|* = 2.12, *p*_fdr_ = .051), or between the second and last block (*|z|* = .92, *p*_fdr_ = .36).

The main effect of Side (*χ*^2^_(1)_ = 55.24, *p*_*fdr*_ < .001) reflected that responses to right-sided stimuli were faster than left-sided ones (*|z|* = 7.4, *p* < .001). The main effect of Block (*χ*^2^_(2)_ = 12.63, *p*_*fdr*_ = .016) reflected slower responses in the second block compared to both the first and last ones (all *|z|* > 2.14, all *p*_fdr_ < .049), while no difference was found between the first and last blocks (*|z|* = 1.41, *p*_*fdr*_ = .16). Finally, GVS and Block interacted (*χ*^2^_(4)_ = 23.63, *p*_*fdr*_ = .001), such that reaction times became slower over time in the sham condition, but faster in the Left-Anodal condition; no effect for Right-Anodal was found. Please refer to Supplementary 3 for more detailed information and additional graphical depictions. The readers interested in spatial effects of a vestibular stimulation might notice that GVS did not interact with the Side of target presentation (*χ*^2^_(2)_ = 3.37, *p*_*fdr*_ = .417), as previously reported in similar tasks (see, for example, a null effect in a temporary order judgement task, Rorden et al., 2001).

### 3.4 Post hoc: robustness checks

In this section, we report secondary analyses and several more robustness checks especially aimed to collect converging statistical evidence for the GVS by Reward interaction, which was found to be significant in our main inferential technique. All details, and further graphical depictions, can be retrieved in Supplementary 5.

*Bayesian ANOVA*. A Bayesian ANOVA was performed with the objective priors described in the methods. This analysis only provided strong evidence (all BF >> 1000) for the main effects of Reward, Validity, and Side. Among the other tests, three were inconclusive: GVS (BF = 1.169), GVS by Block (BF = .613), and Validity by Side (BF = .371); all remaining tests yielded a Bayes Factor favouring the null hypothesis (all BF < .3). The two-way interaction GVS by Reward obtained a Bayes Factor of .124, which favours the null hypothesis by a factor of about 8. We further assessed this specific test in depth because of its importance in this study. Given that results of a Bayesian ANOVA partially depend on the choice of the prior, we varied its width (from .1 to 1, with steps of .1) to perform a sensitivity check. With the narrowest prior scale (*r* = .1) the Bayes Factor for the two-way interaction slightly favoured H1 (BF = 3.186). The Bayes Factors decreased sensibly soon after with wider priors (for *r* = .2 BF = 1.379, for *r* = .3 BF = .554, for wider *r* values all BF < .25). Thus, for this specific test, BFs were not robust across different priors, and the overall results remain ambiguous. In contrast, we then repeated the same procedure for the three-way GVS by Reward by Validity interaction. The results in this case were more conclusive: even with the narrowest prior (*r* = .1) the BF supported the absence of an effect (BF = .19).

*Frequentist ANOVA*. We ran an ANOVA with the same analytic precautions we adopted for mixed models (i.e., corrections for FDR). With respect to the main analysis, we confirm that the main effects of Reward, Validity, and Side were significant, together with the GVS by Reward interaction (*F*_(4, 116)_ = 3.06, *p*_*fdr*_ = .039, *η*_*p*_^*2*^ = .095). All other tests did not reach significance at the FDR threshold.

*Inter-individual variability*. We evaluated the proportion of subjects who showed decreased sensitivity to rewards in the Right-Anodal with respect to the sham condition. We computed the slope coefficients for the effect of Reward separately for each GVS condition. The reaction times were thus predicted by the amount of points allotted for a given trial (0, 2, or 10); overall we had negative slopes, indicating faster reaction times for larger rewards. First, we multiplied these values by −1: larger (positive) values now indicated stronger sensitivity to rewards. Then, for each participant, we subtracted the coefficient obtained in the Right-Anodal condition from that obtained in the sham condition. Differences are thus interpreted as follows: positive values indicate that sensitivity to rewards is reduced in the Right-Anodal condition, whereas negative values indicate that sensitivity to rewards is actually enhanced. We found that 22 subjects over 30 (73.3%) presented reduced sensitivity to rewards in the Right-Anodal GVS with respect to sham (*t*_(29)_ = 3.04, *p* = .005, one-sample, two-tailed). On average, the performance improvement induced by rewards was of .66 msec per point smaller in the Right-Anodal condition with respect to the sham (CI_95%_ of the difference between coefficients: .22–1.11 msec). We have identified this value as the ideal candidate for exploratory analyses assessing correlations (see below).

*RTs distribution*. The distribution of the RTs for correct responses is shown in Fig. 5. Visual inspection suggests that Reward is associated with three clearly distinguishable distributions in the sham condition, whereas the two GVS conditions present comparatively more overlapping distributions. On the contrary, an effect of Reward appears to persist in the long tails of the distributions for the two GVS conditions, but less so for the sham condition. In this study, we had a time limit of 500 msec to induce more challenging demands. Analyses presented so far only analysed RTs for responses that were both correct and given within the time limit, thus discarding what was lying in the long tails. To explore the combined effects of GVS and Reward on the distribution of reaction times as a whole, we computed three different parameters (Whelan, 2008): mu and sigma, representing respectively the mean and standard deviation of the first, Gaussian part of the distribution; tau, representing both the mean and standard deviation of the exponential, rightmost component. The GVS by Reward interaction was highlighted for the mu parameter (*F*_(4, 116)_ = 2.62, *p* = .039, bottom left panel in Fig. 5). The estimates showed a clear displacement of the distribution in the sham condition (356.2, 354.8, and 344.6 msec for the three levels of Reward) that was greatly reduced in Left-Anodal (356.2, 351.9, and 351.8 msec) and Right-Anodal (353.1, 354, and 350.6 msec) conditions. There were no effects on the sigma (*F*_(4, 116)_ = 1.36, *p* = .25) or tau parameters (*F*_(4, 116)_ = 2.01, *p* = .098, bottom right panel in Fig. 5) though the latter was indeed numerically inferior for high rewards in the two GVS conditions. Overall, the results indicate that the time limit given to participants had an important role in the effect described here. It is in principle possible that the reduction in sensitivity to rewards might be especially observed under time-pressure conditions. Indeed, such temporal constraints might be necessary in order to observe effects of motivation over RTs tout court, and consequently their modulation.

*Counterbalancing/Order effects*. Participants became more accurate (from 92.1 to 95.7%) and faster (from 386.9 to 380.3 msec) over the three sessions. We ran an ANOVA with the counterbalancing Order as a factor (with 6 levels) to check whether this could account for some of the effects observed (for example, larger Reward effects in the sham could be seen mostly for participants who started with this condition). There were, however, no effects of or interactions with the Order (all *F* < 1.54, all *p* > .21).

### 3.5 Post hoc: exploratory analyses

A detailed overview of all tests, including more graphical depictions, can be retrieved in Supplementary 5.

*Side of the cue*. The factor Side referred so far to where the *target* was presented on the screen. We ran an ANOVA using the Side of the *cue*, instead of that of the target, in case hemispheric effects of GVS arise for cued locations. The results, with respect to the ANOVA performed above with the preregistered factors, were coherent. The main effects of Reward and Validity, together with the GVS by Reward interaction, remained significant. With respect to the previous analysis, the Side of the cue interacted with Validity (*F*_(1, 29)_ = 9.73, *p*_*fdr*_ = .038, *η*_*p*_^2^ = .25). There was little difference between the Validity gains following right-sided cues, but left-sided cues that were invalid induced faster RTs; in other words, IOR was larger following left-sided cues. This is compatible with the main effect of target Side signalled in the previous analysis, targets appearing in the right hemifield triggering faster reaction times. We also report the three-way GVS by Reward by Side of the cue interaction (*F*_(4, 116)_ = 2.52, *p* = .045, *p*_*fdr*_ = .2, *η*_*p*_^2^ = .08), which only reached significance at the uncorrected threshold. The Bayes Factors for this interaction, even with narrow priors (e.g., BF = .48 with *r* = .1), consistently supported the absence of a modulation. We decided nevertheless to report this result for completeness. The visual inspection of the data (see Fig. 6) suggests that the larger abolishment of sensitivity to rewards in the Right-Anodal condition might be seen – especially, but not only – for cues presented in the left side of space.

*Correlations*. Following the results described above, suggesting that Right-Anodal specifically reduces sensitivity to rewards with respect to the sham condition, we identified a few variables of potential interest, and then ran correlations. For the sake of brevity, here we only report an interesting, though non-significant, trend depicted in Fig. 6. In section 3.4 we computed the slope coefficients for the effect of Reward separately for each GVS condition, and then obtained indices that reflect the amount of reduction of sensitivity to rewards induced by the Right-Anodal condition. We applied a similar procedure for the amount of AC shown by participants in each GVS condition. The three-way GVS by Reward by Validity was not significant in our previous analyses. Nevertheless, we observed a visual trend for Right-Anodal to reduce AC (i.e., enhanced IOR for high rewards). The validity gain for each reward condition (0, 2, 10 points) was thus used as dependent variable, and slopes computed for each level of GVS. With a procedure similar to that described above, we obtained indices reflecting the amount of reduction of AC induced by Right-Anodal with respect to the sham condition. We found a trend between the reduction of sensitivity to reward and the reduction of AC by high rewards following Right-Anodal GVS (*r* = .35, *p* = .064, BF = 1.4; one observation was excluded because its Cook's distance exceeded the 4/N threshold). In other words, participants with the largest reduction of sensitivity to reward tended to also exhibit the largest reduction of AC by high rewards. This was not due to unspecific factors related for example to speed (e.g., faster RTs for one GVS condition), altered perception of the vertical (no correlation with the SVV task), or self-reported feelings of body rotation (no correlation with the values of this specific item of the questionnaire).

### 3.6 Summary of results

According to the pre-registered criteria, we can safely discuss the results because they met both of the mandatory conditions for their interpretation. First, GVS did modulate verticality judgements in the SVV task; second, motivation did speed up performance as measured by the ART. The first result fits well with the literature showing that the SVV is tilted towards the side of anodal stimulation (e.g., Mars et al., 2001). The second confirms that rewards modulate participants' performance, which is greatly ameliorated when high rewards are at stake. Our third criterion, namely the Reward by Validity interaction, was not fulfilled, as we found no evidence for high rewards to enhance IOR. The criterion was important in order to readily and safely interpret a possible three-way interaction with GVS, but we don't have evidence for the presence of such modulation either. We thus conclude that GVS, at least in our setting, does not modulate the interplay between attention and motivation, or that its effect is too small to be meaningful. What we did find, however, was that GVS modulated motivational assets per se. Specifically, the performance gain that is observed with high rewards was greatly reduced with concurrent GVS stimulation. Additionally, there was a tendency for Left-Anodal GVS to be effective in this direction, but we can only provide conclusive statistical evidence for the Right-Anodal condition. This result was proven robust across a range of different frequentist inferential techniques, whereas the Bayesian ANOVA yielded mixed results; some caution remains therefore advisable at this stage. The most interesting finding of post hoc follow-up analyses, though with low evidential value, was a trend for the larger reductions of sensitivity to rewards in the Right-Anodal GVS to correlate with larger reductions of AC by high rewards in the same condition.

## 4 Discussion

With this study we sought to assess whether a perturbation of the vestibular system can affect motivational assets and the interplay between motivation and attention. The vestibular system is phylogenetically old, and it is subserved by a very wide brain network including deep-limbic areas (Lopez et al., 2012). It has a pervasive role in many cognitive functions (Guidetti, 2013, Lopez, 2016, Mast et al., 2014), including affective and emotional processing (Preuss et al., 2014a, Preuss et al., 2014b). A few case reports suggest that (caloric) vestibular stimulation might be beneficial in the treatment of manic disorders (Dodson, 2004, Levine et al., 2012). In addition, patients with vestibular disorders present a range of psychiatric comorbidities that have been suggested to arise directly from the vestibular failure (Gurvich et al., 2013). However, such evidence is sparse, and much is yet unknown about the role of the vestibular system in these domains. We thus aimed at probing the role of the vestibular system in reward processing, because establishing this link would be an important piece of the puzzle and of paramount clinical importance. The vestibular system and reward processing might be functionally tied for evolutionary and adaptive reasons. Visual-vestibular mismatches might be the first signals of noxious reactions to ingested neurotoxins (Treisman, 1977), and might participate, together with nausea, to the process of aversive conditioning. The unspecialized feeders experiencing such mismatches and adverse effects should optimize future foraging behaviours by decreasing the exploration of spaces containing maladaptive items. Following this speculation, we hypothesized that a vestibular simulation might alter the way attention is distributed in space by rewards (i.e., AC by salient stimuli). This was also justified by the fact that vestibular stimulations activate areas (i.e., the aCC) that are known to allocate spatial attention as a function of motivational salience.

Here, we administered GVS (Utz et al., 2010) to 30 healthy subjects engaged in a Posner-like task. Exogenous spatial cues also conveyed motivational information, signalling the number of points that were at stake for a correct and fast response. This way, we could evaluate the effects of GVS on both the mere processing of these cues (rewards) and on their interplay with spatial attention (i.e., the changes in IOR for the different levels of reward). The results, however, failed to highlight the latter interaction: contrary to other studies (Bucker & Theeuwes, 2014), we have no evidence for rewards to enhance IOR. There are, of course, several differences between the present study and that of Bucker and Theeuwes (2014); for example, here rewards were delivered on a trial-by-trial basis (and not in separate blocks), with only one (and not two) SOA, the spatial discrimination task was easier, and the response dimension (up/down buttons) was orthogonal to stimuli lateralization (left/right). We did observe a trend for this effect to be present for left-sided stimuli, but it did not reach statistical significance. We also observed a visual trend for IOR to be enhanced by high rewards in the Left-Anodal and sham conditions, whereas the pattern was reversed for Right-Anodal GVS. Again, this was far from being significant, despite a power estimated to highlight small-to-medium effect sizes. We conclude that either a vestibular stimulation does not affect how attention is allocated in space according to rewarding cues, or this effect is rather small in healthy individuals and/or laboratory settings.

In contrast, here we report that GVS affects the processing of rewards, making participants less sensitive to them. We observed a robust advantage in performance to responses that were signalled by a cue indicating the possibility to receive a reward. However, this performance boost was largely reduced with concurrent GVS: Right-Anodal stimulation appeared more effective, though a tendency was also seen for Left-Anodal GVS (but did not survive corrections for multiple tests). We suggest that this result can indicate a decreased sensitivity to rewards due to the vestibular stimulation. This finding could be interesting for several reasons. First, it is, to the best of our knowledge, the first empirical and causal evidence for a role of the vestibular system in the processing of rewards. Visual-vestibular mismatches have been proposed to participate to the process of aversive conditioning before (Treisman, 1977), but we are not aware of studies directly probing this hypothesis. Second, the effect was found for monetary reward, a secondary-associative type of reward, and not for primary goods (e.g., food), for which the evolutionary hypothesis put forward by Treisman was originally devised. This would perhaps call for an embodied mechanism in charge of the evaluation of diverse motivational stimuli, with its roots in a phylogenetically ancient and adaptive link between visuo-vestibular mismatches or altered bodily states and (ingestion of) noxious substances. Finally, it would also have important clinical applications. An aberrant sensitivity to rewards is a crucial feature of a range of clinical disorders (like depression, apathy, and anhedonia on one side, mania and addiction on the other side of the continuum, e.g., Hyman, Malenka, & Nestler, 2006). Several studies on addiction disorders highlight the role of interoceptive, bodily states in the urge of taking drugs (Naqvi and Bechara, 2009, Naqvi and Bechara, 2010). The insula has been suggested to integrate these interoceptive/visceral feelings into more conscious memory and decision-making processes (Gray and Critchley, 2007, Naqvi and Bechara, 2009, Naqvi and Bechara, 2010). A damage to the insula following stroke reduces addiction to nicotine and the chances of relapse (Naqvi, Rudrauf, Damasio, & Bechara, 2007). While the present study did not include measures of brain activity, we speculate that enhanced activations within the parieto-insular vestibular cortex (Augustine, 1985, Eickhoff et al., 2006, Grüsser et al., 1990), possibly in interaction with the aCC or other limbic areas, might eventually subserve the reduction of sensitivity to rewards that we reported, perhaps in light of a perturbation of autonomic interoceptive states. If this is true – and further (neuroimaging) studies are needed to prove it – GVS might represent a potentially interesting therapeutic adjuvant. Future neuroimaging studies might also shed light on potential hemispheric asymmetries. We indeed observed the largest effects with Right-Anodal stimulation. The Right-Anodal stimulation comparatively activates more bilateral regions, whereas the Left-Anodal stimulation mainly taps onto right-lateralized vestibular regions (e.g., Preuss, Kalla, Müri, & Mast, 2017). One possibility is that the more extensive activations seen with Right-Anodal GVS might explain its prominent role.

Finally, even if with low statistical evidence, we observed an interesting correlation: despite the absence of GVS effects on AC, a weak correlation suggested that larger GVS-induced reduction of sensitivity to rewards (in the Right-Anodal GVS) might be tied to larger reductions of AC by rewards in the same condition. From a clinical perspective, this is also interesting, because the most dramatic AC occurs in patients with addiction disorders and for “ecological” stimuli that are related to the addiction itself (e.g., Field et al., 2009). Here, we tested healthy subjects in a laboratory setting. As a consequence, we don't feel like dismissing entirely the possibility to observe GVS-induced effects on AC, possibly mediated by a perturbed sensitivity to rewards, in populations where AC and sensitivity to rewards are both prominent and distinctive features.

### 4.1 Conclusion

The present study reports an intriguing novel finding: (galvanic) vestibular stimulation, most notably Right-Anodal, reduced motivation-induced performance benefits. We interpreted this finding in terms of decreased sensitivity to reward. Although this was one of the possibilities we envisaged before data collection, the study was – at least with respect to the directionality of our hypotheses – rather explorative. We thus call for more studies exploring this fascinating issue, namely the interaction between the vestibular and motivational systems: neuroimaging studies may shed light on the neural underpinning of this interplay, while clinical studies may probe the utility of GVS as adjuvant in the treatment of addictive disorders.
