# Supplementary material for: Probing the role of the vestibular system in motivation and reward-based attention
Source: Cortex. 2018 Jun;103:82–99. doi: 10.1016/j.cortex.2018.02.009 (PMC6002611; doi:10.1016/j.cortex.2018.02.009)
Supplement: Supplementary file 1 [file mmc1.zip › cortex_2252_mmc1.html]

S1: Subjective Visual Vertical (SVV)


# S1: Subjective Visual Vertical (SVV)

#### *Elvio Blini INSERM U1028, ImpAct team, CRNL, and University of Lyon elvio.blini@gmail.com*

#### *15 November 2017*

# Subjective visual vertical

In this task subjects had to rotate a segment presented on the screen until they perceived it to be vertical.

It was our main control task, administered to assess if GVS was indeed effective. This is because a whealth of literature shows that the subjective vertical is tilted towards the site of anodal stimulation (not to mention results with patients with unilateral vestibular disfunction). Consequently, we assessed this task at the beginning of each of the three GVS sessions, expecting to observe a pattern along the gradient Left-Anodal <- SHAM <- Right-Anodal. Because of some inconsistency in the literature, we decided that - in order to declare GVS effective - at least one of the contrasts had to be significant and in the predicted direction.

We stressed accuracy over velocity. The dependent variable is stored as “FinalOrientation” of the line, once validated by subjects. Lines could rotate with steps of 0.1°. We stored deviation in the anti-clockwise sense as negative values, positive if clockwise. Finally, lines were initially presented on the screen either tilted clockwise or anti-clockwise (in a random, balanced presentation order). We expected this factor to be highly relevant, such that lines originally tilted clockwise would be eventually biased in the same (clockwise) direction, and viceversa.

## Preliminary setup

It’s sometimes good to clean the current environment to avoid conflicts. You can do it with `rm(list=ls())` (but be sure everything is properly saved for future use).

In order to run this script we need a few packages available on CRAN. You might need to install them first, e.g. by typing `install.packages("BayesFactor")` in the console.

```
#list packages
packages= c("plyr", "magrittr", "tidyverse", "BayesFactor", "lme4", "multcomp",
            "gridExtra", "ez" , "lsmeans")

#load them
lapply(packages, require, character.only= T)
```

```
## Warning: package 'tidyverse' was built under R version 3.4.3
```

```
## Warning: package 'tibble' was built under R version 3.4.3
```

```
## Warning: package 'tidyr' was built under R version 3.4.3
```

```
## Warning: package 'purrr' was built under R version 3.4.3
```

For package `afex` I’m using the developer version on github (you will need to install `devtools`).

```
#devtools::install_github("singmann/afex@master")
library(afex)
```

For reproducibility, here’s details of the system on which the script was tested and a seed for random numbers:

```
set.seed(1)

sessionInfo()
```

```
## R version 3.4.2 (2017-09-28)
## Platform: x86_64-w64-mingw32/x64 (64-bit)
## Running under: Windows 10 x64 (build 16299)
## 
## Matrix products: default
## 
## locale:
## [1] LC_COLLATE=English_United Kingdom.1252 
## [2] LC_CTYPE=English_United Kingdom.1252   
## [3] LC_MONETARY=English_United Kingdom.1252
## [4] LC_NUMERIC=C                           
## [5] LC_TIME=English_United Kingdom.1252    
## 
## attached base packages:
## [1] stats     graphics  grDevices utils     datasets  methods   base     
## 
## other attached packages:
##  [1] afex_0.18-0          lsmeans_2.27-2       estimability_1.2    
##  [4] ez_4.4-0             gridExtra_2.3        multcomp_1.4-7      
##  [7] TH.data_1.0-8        MASS_7.3-47          survival_2.41-3     
## [10] mvtnorm_1.0-6        lme4_1.1-14          BayesFactor_0.9.12-2
## [13] Matrix_1.2-11        coda_0.19-1          forcats_0.2.0       
## [16] stringr_1.2.0        dplyr_0.7.4          purrr_0.2.4         
## [19] readr_1.1.1          tidyr_0.7.2          tibble_1.4.1        
## [22] ggplot2_2.2.1        tidyverse_1.2.1      magrittr_1.5        
## [25] plyr_1.8.4           printr_0.1          
## 
## loaded via a namespace (and not attached):
##  [1] nlme_3.1-131        pbkrtest_0.4-7      lubridate_1.7.1    
##  [4] RColorBrewer_1.1-2  httr_1.3.1          rprojroot_1.2      
##  [7] tools_3.4.2         backports_1.1.1     R6_2.2.2           
## [10] rpart_4.1-11        Hmisc_4.1-1         lazyeval_0.2.0     
## [13] mgcv_1.8-20         colorspace_1.3-2    nnet_7.3-12        
## [16] mnormt_1.5-5        compiler_3.4.2      cli_1.0.0          
## [19] rvest_0.3.2         quantreg_5.33       htmlTable_1.11.1   
## [22] SparseM_1.77        xml2_1.1.1          sandwich_2.4-0     
## [25] checkmate_1.8.4     scales_0.5.0        psych_1.7.8        
## [28] pbapply_1.3-3       digest_0.6.12       foreign_0.8-69     
## [31] minqa_1.2.4         rmarkdown_1.7       base64enc_0.1-3    
## [34] pkgconfig_2.0.1     htmltools_0.3.6     htmlwidgets_0.9    
## [37] rlang_0.1.6         readxl_1.0.0        rstudioapi_0.7     
## [40] bindr_0.1           zoo_1.8-0           jsonlite_1.5       
## [43] gtools_3.5.0        acepack_1.4.1       car_2.1-6          
## [46] modeltools_0.2-21   Formula_1.2-2       Rcpp_0.12.13       
## [49] munsell_0.4.3       stringi_1.1.5       yaml_2.1.14        
## [52] grid_3.4.2          parallel_3.4.2      crayon_1.3.4       
## [55] lattice_0.20-35     haven_1.1.0         splines_3.4.2      
## [58] hms_0.3             knitr_1.17          pillar_1.1.0       
## [61] reshape2_1.4.2      codetools_0.2-15    stats4_3.4.2       
## [64] glue_1.2.0          evaluate_0.10.1     latticeExtra_0.6-28
## [67] data.table_1.10.4   modelr_0.1.1        nloptr_1.0.4       
## [70] MatrixModels_0.4-1  cellranger_1.1.0    gtable_0.2.0       
## [73] assertthat_0.2.0    coin_1.2-1          xtable_1.8-2       
## [76] broom_0.4.2         lmerTest_2.0-33     bindrcpp_0.2       
## [79] cluster_2.0.6
```

Thanks to the function retrieved here, not displayed, the following hyperlink downloads the Rdata file:

That can be loaded then with:

```
load("SVV data.RData")
```

Now all relevant variables are stored in the `ART` data.frame, that you can navigate and explore with the usual commands, e.g. `str(SVV)`.

## Useful functions

This is a minimal (shared by all plots) list of graphic attributes for ggplot:

```
#ggplot defaults
commonTheme = list(theme_bw(),
                   theme(text = element_text(size=18, face="bold"),
                         axis.text = element_text(size=16, face="bold", color= "black"),
                         plot.margin = unit(c(1,1,1,1), "cm")))
```

Then (ONLY SHOWN IN THE BOTTOM PART OF THIS SCRIPT) we have a convenient summarising function that produces means and within subjects SEM as in Morey (2008). I retrieved it here: link

## Preprocessing

A bunch of variables is to declare (e.g., numbers into factors). Then, for the sake of clarity, levels are renamed when helpful.

```
#declare
SVV$Subject= as.factor(SVV$Subject)
SVV$GVS= factor(SVV$GVS, labels = c("SHAM", "Left-Anodal", "Right-Anodal"))
SVV$GVS= relevel(SVV$GVS, "Left-Anodal")
```

We must drop a few subjects ( :( ). Reasons are in the comments.

```
#subject 1 to replace for a bug in the ART script, my fault, :(
SVV= SVV[!SVV$Subject== 1, ] #replaced by subject 51

#subject 10 didn't show up after first session, >:(
SVV= SVV[!SVV$Subject== 10, ] #replaced by subject 60

SVV$Subject= factor(SVV$Subject)
```

We registered a trim at ± 2.5 standard deviations for each subject.

```
#create a variables containing normalized values, for each subject
SVV$scaledFO= 0
for (i in levels(SVV$Subject)) {
  SVV$scaledFO[SVV$Subject== i]= scale(SVV$FinalOrientation[SVV$Subject== i])
}

#outlier if more than 2.5 away from the mean
SVV$Outlier= ifelse(abs(SVV$scaledFO)>2.5, 1, 0)
```

We eventually drop the outliers (percentage omitted reported before that):

```
with(SVV, tapply(Outlier, Subject, mean)) %>% mean(na.rm= T)
```

```
## [1] 0.008796296
```

```
SVV= SVV[SVV$Outlier==0,]
```

Here’s what we have as results!

```
#for each subject and GVS session
with(SVV, tapply(FinalOrientation, list(subject_nr, GVS), mean))
```

|  | Left-Anodal | SHAM | Right-Anodal |
| --- | --- | --- | --- |
| 2 | -0.0333333 | 0.4458333 | 0.9000000 |
| 3 | -0.5695652 | -0.2583333 | -0.4333333 |
| 4 | 0.2791667 | 0.6375000 | -0.0833333 |
| 5 | -0.2166667 | -0.3166667 | 1.0916667 |
| 6 | -0.1521739 | 0.1250000 | 0.1166667 |
| 7 | 0.6083333 | 0.6347826 | 0.2500000 |
| 8 | -0.3541667 | 0.2666667 | 0.5958333 |
| 9 | -0.0125000 | -0.0041667 | 0.6333333 |
| 11 | -0.3875000 | 1.2333333 | -0.3291667 |
| 12 | 0.1826087 | 0.0916667 | 0.2791667 |
| 13 | 0.0791667 | -1.1041667 | 1.1583333 |
| 14 | -0.2625000 | 0.3791667 | 0.5304348 |
| 15 | 0.7608696 | 1.6666667 | 2.2083333 |
| 16 | -0.1750000 | 0.1791667 | 0.5250000 |
| 17 | 0.5208333 | 1.1043478 | 1.2916667 |
| 18 | -0.4086957 | -0.9541667 | 0.5608696 |
| 19 | -0.9208333 | -0.3791667 | 0.1250000 |
| 20 | -1.3375000 | -0.0250000 | -0.2782609 |
| 21 | -1.0833333 | 0.0375000 | 0.5041667 |
| 22 | -1.0583333 | -1.0333333 | -0.2666667 |
| 23 | -0.8434783 | -0.9375000 | -0.1217391 |
| 24 | -0.1333333 | -0.6666667 | -0.5565217 |
| 25 | -0.6130435 | -0.0217391 | -0.0416667 |
| 26 | -1.0500000 | -0.3250000 | 0.5333333 |
| 27 | -0.4125000 | -0.1208333 | 1.1750000 |
| 28 | -0.4666667 | 0.1416667 | 0.3250000 |
| 29 | -1.1250000 | -0.6750000 | 0.4826087 |
| 30 | -0.8652174 | -0.5217391 | -0.0875000 |
| 51 | -0.5333333 | -0.2916667 | -0.6625000 |
| 60 | -0.4000000 | -0.4391304 | -0.2750000 |

```
#the group mean
with(SVV, tapply(FinalOrientation, list(subject_nr, GVS), mean)) %>% colMeans(na.rm= T)
```

```
##  Left-Anodal         SHAM Right-Anodal 
##  -0.36612319  -0.03769928   0.33835749
```

```
#the group median
with(SVV, tapply(FinalOrientation, list(subject_nr, GVS), median)) %>% colMeans(na.rm= T)
```

```
##  Left-Anodal         SHAM Right-Anodal 
##  -0.41500000  -0.06833333   0.34666667
```

It looks promising! :)

## Analyses

The main procedure on which we rely is based on mixed linear regression models, explained below and in the manuscript. However, we can start with a basic ANOVA to have a first hint (and then check for consistency between results). For ANOVA data are first collapsed by GVS, StartingSide, and Subject. We then exploit the `ez` package to have a type 2 SS ANOVA. Note that p-values are not adjusted for this analysis.

```
ddply(SVV, c("GVS", "StartingSide", "Subject"), summarise, svv= mean(FinalOrientation)) %>%
ezANOVA(dv= .(svv), wid = .(Subject), within = c("GVS", "StartingSide"), 
        detailed=T, type = 2) %$% 
ANOVA
```

```
## Warning: Converting "StartingSide" to factor for ANOVA.
```

| Effect | DFn | DFd | SSn | SSd | F | p | p<.05 | ges |
| --- | --- | --- | --- | --- | --- | --- | --- | --- |
| (Intercept) | 1 | 29 | 0.0913638 | 42.136922 | 0.0628795 | 0.8037694 |  | 0.0010710 |
| GVS | 2 | 58 | 15.0991585 | 23.488442 | 18.6421730 | 0.0000006 | \* | 0.1505209 |
| StartingSide | 1 | 29 | 14.6062535 | 16.258841 | 26.0523707 | 0.0000190 | \* | 0.1463262 |
| GVS:StartingSide | 2 | 58 | 0.0072507 | 3.329349 | 0.0631567 | 0.9388609 |  | 0.0000851 |

As expected, StartingSide has a huge effect. But, crucially, the main effect of GVS seems to hold. Our secondary analysis was based on a Bayesian ANOVA, as implemented in the `BayesFactor` package (see for example here. We will use objective prior to avoid some degree of freedom.

```
DF= ddply(SVV, c("GVS", "StartingSide", "Subject"), summarise, svv= mean(FinalOrientation)) %>%
    mutate(StartingSide= as.factor(StartingSide)) 

anovaBF(svv ~ GVS*StartingSide + Subject, DF, whichRandom="Subject", 
        whichModels= "bottom", progress=F)
```

When compared against the model 

...

Enter one or more search terms in the box to filter the models in the table. If more than one term is included, matching will be performed with `or`. Special search terms are allowed:

| Code | Function | Example | What example does |
| --- | --- | --- | --- |
| + | Require this term in search results | +shape | Requires all models to include âshapeâ |
| - | Require this term NOT to appear in search results | -shape | Requires all models to exclude âshapeâ |
| # (with number) | Return results with certain number of terms | #2 | Requires all models to have two terms |
| @ (with :, ::, â¦) | Return results containing interactions of certain size | @:: | Requires all models to have a three-way interaction |
| < or > | Limits sizes of Bayes factors | >2 | Returns models whose Bayes factor is greater than 2 |

Click on a row in the Bayes factor table to make that model the denominator. Sort by clicking on the arrows in the column headers.

|  |  |  |
| --- | --- | --- |
| ...the model below... | ...is preferred by... |  |

From which we see that the preferred model is the one including both main effects of GVS and StartingSide, but not the interaction.

We can now start with model selection procedure. The general strategy is to evaluate beforehand the random effects that increase model fitting, as to reach a parsimonious solution (i.e. supported by data). We create several different (nested) models and evaluate them against a simpler reference one through likelihood ratio tests (LRT). This holds for both random and fixed effects testing.

The simplest model to start with only includes the random intercept for Subjects (baseline level). We then start testing random slopes one by one, following this order:

1. GVS
2. StartingSide

Each random slope - that informs about variability in performance across levels of a factor, e.g. differences in experimental manipulations across subjects - will be retained in the model if the LRT is proven significant. Following evaluations will be made with reference models that include this slope. For example, if GVS improves model fit as random slope, StartingSide will be evaluated against the model including it. As a second step we introduce interactions for all combination of slopes proven significant (this is to respect marginality, thus include high-order terms together with their lower-order ones).

Fixed effects testing will use a similar (type 2) approach.

This is the starting model:

```
svvmod0= lmer(FinalOrientation ~ (1|Subject), data= SVV, REML=T,
            control=lmerControl(optimizer="bobyqa"))
```

Note that: we asked for the bobyqa algorithm, which handles convergence problems very well; we asked for restricted maximum likelihood fitting in this stage (we will switch to maximum likelihood for fixed effects). The first slope to be evaluated is for GVS:

```
svvmod0a= lmer(FinalOrientation ~ (1+GVS|Subject), data= SVV, REML=T,
            control=lmerControl(optimizer="bobyqa"))
```

We can test for its role with a LRT:

```
anova(svvmod0, svvmod0a, refit= F)
```

|  | Df | AIC | BIC | logLik | deviance | Chisq | Chi Df | Pr(>Chisq) |
| --- | --- | --- | --- | --- | --- | --- | --- | --- |
| svvmod0 | 3 | 5136.19 | 5153.197 | -2565.095 | 5130.19 | NA | NA | NA |
| svvmod0a | 8 | 4458.68 | 4504.032 | -2221.340 | 4442.68 | 687.51 | 5 | 0 |

It is very important (also look at AIC and BIC criteria!). We move to StartingSide:

```
svvmod0b= lmer(FinalOrientation ~ (1+GVS+StartingSide|Subject), data= SVV, REML=T,
            control=lmerControl(optimizer="bobyqa"))

anova(svvmod0a, svvmod0b, refit= F)
```

|  | Df | AIC | BIC | logLik | deviance | Chisq | Chi Df | Pr(>Chisq) |
| --- | --- | --- | --- | --- | --- | --- | --- | --- |
| svvmod0a | 8 | 4458.680 | 4504.032 | -2221.340 | 4442.680 | NA | NA | NA |
| svvmod0b | 12 | 3417.421 | 3485.450 | -1696.711 | 3393.421 | 1049.259 | 4 | 0 |

It is also incredibly important! Next: the two-way interaction.

```
svvmod0c= lmer(FinalOrientation ~ (1+GVS*StartingSide|Subject), data= SVV, REML=T,
            control=lmerControl(optimizer="bobyqa"))

anova(svvmod0b, svvmod0c, refit= F)
```

|  | Df | AIC | BIC | logLik | deviance | Chisq | Chi Df | Pr(>Chisq) |
| --- | --- | --- | --- | --- | --- | --- | --- | --- |
| svvmod0b | 12 | 3417.421 | 3485.450 | -1696.711 | 3393.421 | NA | NA | NA |
| svvmod0c | 23 | 3379.572 | 3509.959 | -1666.786 | 3333.572 | 59.84964 | 11 | 0 |

The two-way interaction also appear to play some role. We have finally chosen our model, let’s switch to fixed effects exploiting the `afex::mixed` function:

```
svv_models= mixed(FinalOrientation ~ GVS*StartingSide + (1+GVS*StartingSide|Subject),
               expand_re = F, all_fit = FALSE,  
               data= SVV, method= "LRT",  type= "2",
               control= lmerControl(optimizer="bobyqa", optCtrl=list(maxfun=500000)))
```

```
## Contrasts set to contr.sum for the following variables: GVS, Subject
```

```
## REML argument to lmer() set to FALSE for method = 'PB' or 'LRT'
```

```
## Fitting 5 (g)lmer() models:
## [.....]
```

We can display results with:

```
svv_models %>% nice
```

| Effect | df | Chisq | p.value |
| --- | --- | --- | --- |
| GVS | 2 | 30.19 \*\*\* | <.0001 |
| StartingSide | 1 | 19.71 \*\*\* | <.0001 |
| GVS:StartingSide | 2 | 0.13 | .94 |

In which we can see that both main effects are significant! However, there results are uncorrected for multiple testing. We declared our interesting tests to be the GVS main effect and its interaction with starting side. We apply a fdr correction to these two p-values:

```
p.adjust(c(svv_models$anova_table["GVS", ]$`Pr(>Chisq)`,
           svv_models$anova_table["GVS:StartingSide", ]$`Pr(>Chisq)`), "fdr")
```

```
## [1] 5.555172e-07 9.366978e-01
```

The main effect of GVS remains significant after correction. Furthermore - because we planned sequential analyses at 24 and then 30 subjects - the main effect of GVS also survives correction for multiple longitudinal data assessment (equals to set a new alpha level of 0.043 instead of 0.05). We now run post-hoc tests to see which contrast reaches significance.

```
lsm.options(lmer.df = "asymptotic") # the safest and fastest, no df
lsmeans(svv_models, pairwise ~ GVS, adjust= "fdr")$contrast
```

```
## lsmeans are based on full model which includes all effects.
```

```
## NOTE: Results may be misleading due to involvement in interactions
```

```
##  contrast                     estimate         SE df z.ratio p.value
##  Left-Anodal - SHAM         -0.3329847 0.09996524 NA  -3.331  0.0013
##  Left-Anodal - Right-Anodal -0.7092301 0.10870559 NA  -6.524  <.0001
##  SHAM - Right-Anodal        -0.3762454 0.13172313 NA  -2.856  0.0043
## 
## Results are averaged over the levels of: StartingSide 
## P value adjustment: fdr method for 3 tests
```

## Plots

Here’s a function to plot data, with option to display variability and data distribution:

```
my_plot= function(DF, dv= c("FinalOrientation"), withinvars, betweenvars= NULL, 
                  idvar= "Subject", 
                  individual_data= c("none", "p", "l", "violin", "bp")){
  
  individual_data= match.arg(individual_data)
  dv= match.arg(dv)
  
  allvars= c(withinvars, betweenvars)
  
  Xs= ddply(DF, c(allvars, idvar), summarise, y= mean(get(dv)))
  
  X= summarySEwithin(Xs, measurevar = "y", 
                     betweenvars = betweenvars, 
                     withinvars = withinvars, idvar = idvar)
  
  if(dv=="FinalOrientation")(ylab= "SVV (°)") else(ylab= "Self report")
  
  p= ggplot(X, aes(y= y, x= get(allvars[1]), fill= get(allvars[1]))) + commonTheme +
    geom_hline(yintercept = 0, linetype= "dashed", color= "dark gray", size= 1.1)
  
  if(individual_data=="l"){
    p= p + geom_line(data = Xs, aes(x= get(allvars[1]), y=  y, group= get(idvar)), 
                     size= 1.2, color= "dark gray", alpha= 0.5)} 
  if(individual_data=="p"){
    pd= position_dodge(0.5)
    p= p + geom_point(data = Xs, aes(x= get(allvars[1]), y=  y, group= get(idvar)),
                      position = pd, size= 3, shape= 21, color= "black", alpha= 0.3)} 
  if(individual_data=="violin"){
    p= p + geom_violin(data = Xs, aes(x= get(allvars[1]), y=  y), alpha= 0.3)} 
 
  if(individual_data=="bp"){
    p= p + geom_boxplot(data = Xs, aes(x= get(allvars[1]), y=  y), alpha= 0.3, 
                        outlier.size = 3.5)} 
  
  if(individual_data=="none"){
    p= p + geom_errorbar(data=X, aes(ymin= y-se, ymax= y+se), 
                       size= 1.5, width= .2, colour= "black") + 
           geom_point(size= 6, stroke= 2, shape= 21, color= "black")}
  
  p= p  + labs(y= ylab, x= allvars[1]) + guides(fill=F)
  
  if(length(allvars)>1) (p= p + facet_wrap(allvars[2:length(allvars)]))
  
  return(p)  
  
}
```

We can thus explore both StartingSide main effect:

```
my_plot(SVV, dv <- "FinalOrientation", withinvars = "StartingSide", individual_data = "none")
```

```
## Automatically converting the following non-factors to factors: StartingSide
```

Showing that starting with a line rotated counter-clockwise results into counter-clockwise bias, and viceversa.

Then the main effect of GVS:

```
my_plot(SVV, dv <- "FinalOrientation", withinvars = "GVS", individual_data = "none")
```

Shows that the subjective visual vertical is tilted towards the anodal side of stimulation. The effect does not appear to interact with StartingSide:

```
my_plot(SVV, dv = "FinalOrientation", withinvars = c("GVS", "StartingSide"), 
        individual_data = "none")
```

```
## Automatically converting the following non-factors to factors: StartingSide
```

Finally, here’s how data is distributed for the main effect of GVS:

```
p1= my_plot(SVV, dv <- "FinalOrientation", withinvars = "GVS", individual_data = "p")
p2= my_plot(SVV, dv = "FinalOrientation", withinvars = "GVS", individual_data = "l")
p3= my_plot(SVV, dv = "FinalOrientation", withinvars = "GVS", individual_data = "violin")
p4= my_plot(SVV, dv = "FinalOrientation", withinvars = "GVS", individual_data = "bp")
 
grid.arrange(p1, p2, p3, p4)
```

## Appendix

This is the function to summarise data (`summarySEwithin`):

```
## Summarizes data.
## Gives count, mean, standard deviation, standard error of the mean, and confidence interval (default 95%).
##   data: a data frame.
##   measurevar: the name of a column that contains the variable to be summariezed
##   groupvars: a vector containing names of columns that contain grouping variables
##   na.rm: a boolean that indicates whether to ignore NA's
##   conf.interval: the percent range of the confidence interval (default is 95%)
summarySE <- function(data=NULL, measurevar, groupvars=NULL, na.rm=FALSE,
                      conf.interval=.95, .drop=TRUE) {
  library(plyr)
  
  # New version of length which can handle NA's: if na.rm==T, don't count them
  length2 <- function (x, na.rm=FALSE) {
    if (na.rm) sum(!is.na(x))
    else       length(x)
  }
  
  # This does the summary. For each group's data frame, return a vector with
  # N, mean, and sd
  datac <- ddply(data, groupvars, .drop=.drop,
                 .fun = function(xx, col) {
                   c(N    = length2(xx[[col]], na.rm=na.rm),
                     mean = mean   (xx[[col]], na.rm=na.rm),
                     sd   = sd     (xx[[col]], na.rm=na.rm)
                   )
                 },
                 measurevar
  )
  
  # Rename the "mean" column    
  datac <- plyr::rename(datac, c(mean = measurevar))
  
  datac$se <- datac$sd / sqrt(datac$N)  # Calculate standard error of the mean
  
  # Confidence interval multiplier for standard error
  # Calculate t-statistic for confidence interval: 
  # e.g., if conf.interval is .95, use .975 (above/below), and use df=N-1
  ciMult <- qt(conf.interval/2 + .5, datac$N-1)
  datac$ci <- datac$se * ciMult
  
  return(datac)
}


## Norms the data within specified groups in a data frame; it normalizes each
## subject (identified by idvar) so that they have the same mean, within each group
## specified by betweenvars.
##   data: a data frame.
##   idvar: the name of a column that identifies each subject (or matched subjects)
##   measurevar: the name of a column that contains the variable to be summariezed
##   betweenvars: a vector containing names of columns that are between-subjects variables
##   na.rm: a boolean that indicates whether to ignore NA's
normDataWithin <- function(data=NULL, idvar, measurevar, betweenvars=NULL,
                           na.rm=FALSE, .drop=TRUE) {
  library(plyr)
  
  # Measure var on left, idvar + between vars on right of formula.
  data.subjMean <- ddply(data, c(idvar, betweenvars), .drop=.drop,
                         .fun = function(xx, col, na.rm) {
                           c(subjMean = mean(xx[,col], na.rm=na.rm))
                         },
                         measurevar,
                         na.rm
  )
  
  # Put the subject means with original data
  data <- merge(data, data.subjMean)
  
  # Get the normalized data in a new column
  measureNormedVar <- paste(measurevar, "_norm", sep="")
  data[,measureNormedVar] <- data[,measurevar] - data[,"subjMean"] +
    mean(data[,measurevar], na.rm=na.rm)
  
  # Remove this subject mean column
  data$subjMean <- NULL
  
  return(data)
}


## Retrieved here: http://www.cookbook-r.com/Graphs/Plotting_means_and_error_bars_(ggplot2)/
## Summarizes data, handling within-subjects variables by removing inter-subject variability.
## It will still work if there are no within-S variables.
## Gives count, un-normed mean, normed mean (with same between-group mean),
##   standard deviation, standard error of the mean, and confidence interval.
## If there are within-subject variables, calculate adjusted values using method from Morey (2008).
##   data: a data frame.
##   measurevar: the name of a column that contains the variable to be summariezed
##   betweenvars: a vector containing names of columns that are between-subjects variables
##   withinvars: a vector containing names of columns that are within-subjects variables
##   idvar: the name of a column that identifies each subject (or matched subjects)
##   na.rm: a boolean that indicates whether to ignore NA's
##   conf.interval: the percent range of the confidence interval (default is 95%)
summarySEwithin <- function(data=NULL, measurevar, betweenvars=NULL, withinvars=NULL,
                            idvar=NULL, na.rm=FALSE, conf.interval=.95, .drop=TRUE) {
  
  # Ensure that the betweenvars and withinvars are factors
  factorvars <- vapply(data[, c(betweenvars, withinvars), drop=FALSE],
                       FUN=is.factor, FUN.VALUE=logical(1))
  
  if (!all(factorvars)) {
    nonfactorvars <- names(factorvars)[!factorvars]
    message("Automatically converting the following non-factors to factors: ",
            paste(nonfactorvars, collapse = ", "))
    data[nonfactorvars] <- lapply(data[nonfactorvars], factor)
  }
  
  # Get the means from the un-normed data
  datac <- summarySE(data, measurevar, groupvars=c(betweenvars, withinvars),
                     na.rm=na.rm, conf.interval=conf.interval, .drop=.drop)
  
  # Drop all the unused columns (these will be calculated with normed data)
  datac$sd <- NULL
  datac$se <- NULL
  datac$ci <- NULL
  
  # Norm each subject's data
  ndata <- normDataWithin(data, idvar, measurevar, betweenvars, na.rm, .drop=.drop)
  
  # This is the name of the new column
  measurevar_n <- paste(measurevar, "_norm", sep="")
  
  # Collapse the normed data - now we can treat between and within vars the same
  ndatac <- summarySE(ndata, measurevar_n, groupvars=c(betweenvars, withinvars),
                      na.rm=na.rm, conf.interval=conf.interval, .drop=.drop)
  
  # Apply correction from Morey (2008) to the standard error and confidence interval
  #  Get the product of the number of conditions of within-S variables
  nWithinGroups    <- prod(vapply(ndatac[,withinvars, drop=FALSE], FUN=nlevels,
                                  FUN.VALUE=numeric(1)))
  correctionFactor <- sqrt( nWithinGroups / (nWithinGroups-1) )
  
  # Apply the correction factor
  ndatac$sd <- ndatac$sd * correctionFactor
  ndatac$se <- ndatac$se * correctionFactor
  ndatac$ci <- ndatac$ci * correctionFactor
  
  # Combine the un-normed means with the normed results
  merge(datac, ndatac)
}
```
