# Supplementary material for: Probing the role of the vestibular system in motivation and reward-based attention
Source: Cortex. 2018 Jun;103:82–99. doi: 10.1016/j.cortex.2018.02.009 (PMC6002611; doi:10.1016/j.cortex.2018.02.009)
Supplement: Supplementary file 2 [file mmc2.zip › cortex_2252_mmc2.html]

S2: GVS Evaluation


# S2: GVS Evaluation

#### *Elvio Blini INSERM U1028, ImpAct team, CRNL, and University of Lyon elvio.blini@gmail.com*

#### *15 November 2017*

# Subjective Evaluation of GVS effects

After each GVS session, we asked subjects a few questions to explore their subjective experiences. The aim was twofold:

1. Ensure that the three sessions were comparable in terms of reported distress/feelings, especially when it comes to “arousal” effects (because more specifically vestibular sensations, e.g. illusions of movement, might indeed characterize active vestibular stimulation sessions with respect to the SHAM condition);
2. Potentially correlate these self-report with interesting indices obtained from the principal task of this study, the ART.

The choice of asking for self report after GVS was necessary to reduce brain stimulation time and leave space to the main tasks. However, this could have hampered to some extent a proper and accurate report of usually short-lasting sensations. Note, anyway, that we rely on an objective task to test GVS effectiveness (SVV, administered at the beginning of the stimulation).

We presented several questions, in fixed-order, on top of a computer screen. Below the questions an horizontal line was depicted, and at each side the response keys allowing to set a response space continuum were written. For example, for the question “I felt nausea” the line was flanked by the sentences “Not at all” and “Very much”. Subjects had to click to the position on the line that best matched their subjective experience. We stored the percent deviation of the response with respect to the center of the segment. We sought to introduce in this way more variability for later analyses, though note that at least one paper suggests that GVS might shift the perceived center of visual segments towards the anodal site (e.g. Ferré et al., 2013).

Being this part exploratory, we did not register specific analyses. We only ran Bayesian ANOVAs to check for a GVS effect across all questions, setting a threshold to a BF of at least 10 in order to consider a finding relevant/interesting. As you will see, quite all questions actually firmly support the absence of a (at least explicitly) perceived difference between conditions, allowing us to conclude that the SHAM condition effectively controlled for unspecific effects of brain stimulation such as tingling or itching (or discomfort in general).

## Preliminary setup

It’s sometimes good to clean the current environment to avoid conflicts. You can do it with `rm(list=ls())` (but be sure everything is properly saved for future use).

In order to run this script we need a few packages available on CRAN. You might need to install them first, e.g. by typing `install.packages("BayesFactor")` in the console.

```
#list packages
packages= c("plyr", "magrittr", "tidyverse", "BayesFactor", "lme4", "multcomp",
            "gridExtra", "ez" , "lsmeans")

#load them
lapply(packages, require, character.only= T)
```

For reproducibility, here’s details of the system on which the script was tested and a seed for random numbers:

```
set.seed(1)

sessionInfo()
```

```
## R version 3.4.2 (2017-09-28)
## Platform: x86_64-w64-mingw32/x64 (64-bit)
## Running under: Windows 10 x64 (build 15063)
## 
## Matrix products: default
## 
## locale:
## [1] LC_COLLATE=English_United Kingdom.1252 
## [2] LC_CTYPE=English_United Kingdom.1252   
## [3] LC_MONETARY=English_United Kingdom.1252
## [4] LC_NUMERIC=C                           
## [5] LC_TIME=English_United Kingdom.1252    
## 
## attached base packages:
## [1] stats     graphics  grDevices utils     datasets  methods   base     
## 
## other attached packages:
##  [1] lsmeans_2.27-2       estimability_1.2     ez_4.4-0            
##  [4] gridExtra_2.3        multcomp_1.4-7       TH.data_1.0-8       
##  [7] MASS_7.3-47          survival_2.41-3      mvtnorm_1.0-6       
## [10] lme4_1.1-14          BayesFactor_0.9.12-2 Matrix_1.2-11       
## [13] coda_0.19-1          dplyr_0.7.4          purrr_0.2.3         
## [16] readr_1.1.1          tidyr_0.7.1          tibble_1.3.4        
## [19] ggplot2_2.2.1        tidyverse_1.1.1      magrittr_1.5        
## [22] plyr_1.8.4           printr_0.1          
## 
## loaded via a namespace (and not attached):
##  [1] httr_1.3.1         jsonlite_1.5       splines_3.4.2     
##  [4] modelr_0.1.1       gtools_3.5.0       assertthat_0.2.0  
##  [7] cellranger_1.1.0   yaml_2.1.14        backports_1.1.1   
## [10] lattice_0.20-35    quantreg_5.33      glue_1.1.1        
## [13] digest_0.6.12      rvest_0.3.2        minqa_1.2.4       
## [16] colorspace_1.3-2   sandwich_2.4-0     htmltools_0.3.6   
## [19] psych_1.7.8        pkgconfig_2.0.1    broom_0.4.2       
## [22] SparseM_1.77       haven_1.1.0        xtable_1.8-2      
## [25] scales_0.5.0       MatrixModels_0.4-1 mgcv_1.8-20       
## [28] car_2.1-5          pbapply_1.3-3      nnet_7.3-12       
## [31] lazyeval_0.2.0     pbkrtest_0.4-7     mnormt_1.5-5      
## [34] readxl_1.0.0       evaluate_0.10.1    nlme_3.1-131      
## [37] forcats_0.2.0      xml2_1.1.1         foreign_0.8-69    
## [40] tools_3.4.2        hms_0.3            stringr_1.2.0     
## [43] munsell_0.4.3      bindrcpp_0.2       compiler_3.4.2    
## [46] rlang_0.1.2        grid_3.4.2         nloptr_1.0.4      
## [49] rmarkdown_1.7      gtable_0.2.0       codetools_0.2-15  
## [52] reshape2_1.4.2     R6_2.2.2           zoo_1.8-0         
## [55] lubridate_1.6.0    knitr_1.17         bindr_0.1         
## [58] rprojroot_1.2      stringi_1.1.5      parallel_3.4.2    
## [61] Rcpp_0.12.13
```

Thanks to the function retrieved here, not displayed, the following hyperlink downloads the Rdata file:

That can be loaded then with:

```
load("EST data.RData")
```

Now all relevant variables are stored in the `EST` data.frame, that you can navigate and explore with the usual commands, e.g. `str(EST)`.

## Useful functions

This is a minimal (shared by all plots) list of graphic attributes for ggplot:

```
#ggplot defaults
commonTheme = list(theme_bw(),
                   theme(text = element_text(size=18, face="bold"),
                         axis.text = element_text(size=16, face="bold", color= "black"),
                         plot.margin = unit(c(1,1,1,1), "cm")))
```

Then (ONLY SHOWN IN THE BOTTOM PART OF THIS SCRIPT) we have a convenient summarising function that produces means and within subjects SEM as in Morey (2008). I retrieved it here: link

## Preprocessing

I’ll be translating French questions into English (some ASCII character messed up in conversion, but it’s not a big deal):

```
FraToEngQuestions= list("I felt my body moving", "I felt my body moving",
                "I felt my body rotating", "I felt my head leaning",
                "I felt the visual scene moving", "I felt vertigo",
                "I felt nausea", "I felt my body or one part of it changing size",
                "I felt itching", "I felt burning",
                "I felt uncomfortable", "I was focused on the task",
                "I felt motivated in accomplishing the task", "I felt motivated by rewards")

FraToEngQuestions= setNames(FraToEngQuestions, unique(EST$Question))

#check
names(FraToEngQuestions) %in% EST$Question
```

```
##  [1] TRUE TRUE TRUE TRUE TRUE TRUE TRUE TRUE TRUE TRUE TRUE TRUE TRUE TRUE
```

```
#now substitute
EST$Eng_Question= unlist(FraToEngQuestions[EST$Question])
```

The same for response keys:

```
#same for responses left / right
FraToEngLeft= list("Left", "Downward", "Backward", "Not at all")
FraToEngRight= list("Right", "Upward", "Forward", "Very much")

FraToEngLeft= setNames(FraToEngLeft, unique(EST$RespLeft))
FraToEngRight= setNames(FraToEngRight, unique(EST$RespRight))

EST$Eng_Left= unlist(FraToEngLeft[EST$RespLeft])
EST$Eng_Right= unlist(FraToEngRight[EST$RespRight])
```

A bunch of variables is to declare (e.g., numbers into factors). Then, for the sake of clarity, levels are renamed when helpful.

```
#declare
EST$Subject= as.factor(EST$Subject)

if(sum(levels(as.factor(EST$GVS))== c("A", "B", "C"))==3){
EST$GVS= factor(EST$GVS, labels = c("SHAM", "Left-Anodal", "Right-Anodal"))
EST$GVS= relevel(EST$GVS, "Left-Anodal")} else (warning("levels unordered? Check"))
```

We must drop a few subjects ( :( ). Reasons are in the comments.

```
#subject 1 to replace for a bug in the script, my fault, :(
EST= EST[!EST$Subject== 1, ] #replaced by subject 51

#subject 10 didn't show up after first session, >:(
EST= EST[!EST$Subject== 10, ] #replaced by subject 60

EST$Subject= factor(EST$Subject)
```

I forgot to add a variable specifying the question’s number, it will help:

```
#turn-around because there were three similar questions...
EST$Quest_Nb= 0
interaction= interaction(EST$Subject, EST$GVS)

invisible(sapply(levels(interaction(EST$Subject, EST$GVS)), function(x){
  EST$Quest_Nb[interaction== x] <<- 1:15
}))
```

We have all set up. We now convert the dependent variable into percent deviation. Consider that the response is already stored as deviation (pixels) from the objective center of the screen (and thus of the segment), and that the segment is 700 pixels long.

```
#half-length of the segment is 350 pixels
EST$X_Dev= EST$X_Resp/3.50
```

Now we have values of -100 for extreme leftward responses and of 100 for extreme rightward responses. Because mouse-click was not super precise we might have in some cases values of -101% (subject went slightly out of the line border).

## Depiction and Analyses

Here’s a function to plot data, with option to display variability and data distribution, which automatically computes the BF (base 10) of the GVS main effect and prints the frequentist ANOVA by default:

```
my_plot= function(DF, dv= c("X_Dev"), withinvars, betweenvars= NULL, 
                  idvar= "Subject", 
                  individual_data= c("none", "p", "l", "violin", "bp"),
                  compute_BF= T, compute_ANOVA=T){
  
  individual_data= match.arg(individual_data)
  dv= match.arg(dv)
  
  allvars= c(withinvars, betweenvars)
  
  Xs= ddply(DF, c(allvars, idvar), summarise, y= mean(get(dv)))
  
  X= summarySEwithin(Xs, measurevar = "y", 
                     betweenvars = betweenvars, 
                     withinvars = withinvars, idvar = idvar)
  
  if(dv=="FinalOrientation")(ylab= "SVV (??)") else(ylab= "Self report")
  
  p= ggplot(X, aes(y= y, x= get(allvars[1]), fill= get(allvars[1]))) + commonTheme +
    geom_hline(yintercept = 0, linetype= "dashed", color= "gray", size= 1.1)
  
  if(individual_data=="l"){
    p= p + geom_line(data = Xs, aes(x= get(allvars[1]), y=  y, group= get(idvar)), 
                     size= 1.2, color= "dark gray", alpha= 0.5)} 
  if(individual_data=="p"){
    pd= position_dodge(0.5)
    p= p + geom_point(data = Xs, aes(x= get(allvars[1]), y=  y, group= get(idvar)),
                      position = pd, size= 3, shape= 21, color= "black", alpha= 0.3)} 
  if(individual_data=="violin"){
    p= p + geom_violin(data = Xs, aes(x= get(allvars[1]), y=  y), alpha= 0.3)} 
  if(individual_data=="bp"){
    p= p + geom_boxplot(data = Xs, aes(x= get(allvars[1]), y=  y), alpha= 0.3,
                        outlier.size = 3.5)} 
  
  p= p + geom_errorbar(data=X, aes(ymin= y-se, ymax= y+se), 
                       size= 1.5, width= .2, colour= "black") + 
         geom_point(size= 6, stroke= 2, shape= 21, color= "black") + 
         labs(y= ylab, x= allvars[1]) + guides(fill=F)
  
  if(length(allvars)>1) (p= p + facet_wrap(allvars[2:length(allvars)]))
  
  if(dv=="X_Dev"){
    title= DF$Eng_Question[1]
    R_Left= DF$Eng_Left[1]
    R_Right= DF$Eng_Right[1]
    labels= c(R_Left, "-50%", "<< 0 >>", "50%", R_Right)
    
    p= p + ggtitle(title) + scale_y_continuous(lim= c(-110, 110), labels= labels)
  }
  
  p= p+ coord_flip()
  
  if(compute_BF){
    bf= anovaBF(y ~ GVS + Subject, Xs, whichRandom = "Subject", progress = F) %>%
      extractBF() %$% bf
    
    txt= paste("BF[10] == ", format(bf, digits= 2))
    
    p= p + annotate(geom = "text", x= 0.5, y= 25, size= 8, fontface =2, hjust= 0,
                   label= txt, parse= T)
    
  }
  
  if(compute_ANOVA){
    EZ= ezANOVA(Xs, dv= .(y), wid= .(Subject), within= .(GVS), type= 2)
    print(EZ)
  }
  
  return(p)  
  
}
```

We can thus explore conveniently all items. Remember that a BF >1 supports a difference across at least one of the three GVS conditions; a BF <1 actually sopports the *lack* of a difference. Results of about 1 are actually fairly ambiguous, we have decided to consider a result informative if BF< 0.1 | BF>10.

```
for (i in 1:15){
    p= my_plot(EST[EST$Quest_Nb==i, ], dv <- "X_Dev", withinvars = "GVS", individual_data = "violin")
    print(p)
}
```

```
## $ANOVA
##   Effect DFn DFd        F         p p<.05        ges
## 2    GVS   2  58 2.053381 0.1375258       0.04055268
## 
## $`Mauchly's Test for Sphericity`
##   Effect         W         p p<.05
## 2    GVS 0.8853398 0.1817773      
## 
## $`Sphericity Corrections`
##   Effect       GGe     p[GG] p[GG]<.05      HFe     p[HF] p[HF]<.05
## 2    GVS 0.8971344 0.1430978           0.952521 0.1400922
```

```
## $ANOVA
##   Effect DFn DFd         F         p p<.05        ges
## 2    GVS   2  58 0.7566337 0.4738182       0.01385603
## 
## $`Mauchly's Test for Sphericity`
##   Effect         W        p p<.05
## 2    GVS 0.9602512 0.566745      
## 
## $`Sphericity Corrections`
##   Effect       GGe     p[GG] p[GG]<.05      HFe     p[HF] p[HF]<.05
## 2    GVS 0.9617707 0.4692343           1.028684 0.4738182
```

```
## $ANOVA
##   Effect DFn DFd       F         p p<.05      ges
## 2    GVS   2  58 1.50035 0.2315805       0.021573
## 
## $`Mauchly's Test for Sphericity`
##   Effect         W         p p<.05
## 2    GVS 0.9049195 0.2469103      
## 
## $`Sphericity Corrections`
##   Effect       GGe    p[GG] p[GG]<.05       HFe     p[HF] p[HF]<.05
## 2    GVS 0.9131749 0.232903           0.9713545 0.2320531
```

```
## $ANOVA
##   Effect DFn DFd        F          p p<.05        ges
## 2    GVS   2  58 3.237322 0.04646567     * 0.06193765
## 
## $`Mauchly's Test for Sphericity`
##   Effect         W          p p<.05
## 2    GVS 0.8123749 0.05452441      
## 
## $`Sphericity Corrections`
##   Effect       GGe      p[GG] p[GG]<.05       HFe      p[HF] p[HF]<.05
## 2    GVS 0.8420166 0.05585261           0.8881434 0.05293392
```

```
## $ANOVA
##   Effect DFn DFd        F         p p<.05        ges
## 2    GVS   2  58 1.466403 0.2391815       0.03029136
## 
## $`Mauchly's Test for Sphericity`
##   Effect         W         p p<.05
## 2    GVS 0.8916292 0.2007151      
## 
## $`Sphericity Corrections`
##   Effect       GGe     p[GG] p[GG]<.05       HFe     p[HF] p[HF]<.05
## 2    GVS 0.9022252 0.2402645           0.9584934 0.2396974
```

```
## $ANOVA
##   Effect DFn DFd         F         p p<.05         ges
## 2    GVS   2  58 0.3580603 0.7005652       0.007326208
## 
## $`Mauchly's Test for Sphericity`
##   Effect         W          p p<.05
## 2    GVS 0.8389088 0.08550792      
## 
## $`Sphericity Corrections`
##   Effect       GGe     p[GG] p[GG]<.05       HFe     p[HF] p[HF]<.05
## 2    GVS 0.8612588 0.6691188           0.9105592 0.6808696
```

```
## $ANOVA
##   Effect DFn DFd        F         p p<.05        ges
## 2    GVS   2  58 1.091121 0.3426331       0.01714366
## 
## $`Mauchly's Test for Sphericity`
##   Effect         W         p p<.05
## 2    GVS 0.9490999 0.4812457      
## 
## $`Sphericity Corrections`
##   Effect       GGe    p[GG] p[GG]<.05     HFe     p[HF] p[HF]<.05
## 2    GVS 0.9515652 0.340455           1.01661 0.3426331
```

```
## $ANOVA
##   Effect DFn DFd        F        p p<.05        ges
## 2    GVS   2  58 1.623071 0.206124       0.03585497
## 
## $`Mauchly's Test for Sphericity`
##   Effect         W         p p<.05
## 2    GVS 0.8598002 0.1206605      
## 
## $`Sphericity Corrections`
##   Effect       GGe     p[GG] p[GG]<.05       HFe     p[HF] p[HF]<.05
## 2    GVS 0.8770392 0.2094373           0.9289896 0.2081034
```

```
## $ANOVA
##   Effect DFn DFd        F         p p<.05        ges
## 2    GVS   2  58 1.720412 0.1880001       0.02718008
## 
## $`Mauchly's Test for Sphericity`
##   Effect         W         p p<.05
## 2    GVS 0.9118634 0.2748001      
## 
## $`Sphericity Corrections`
##   Effect       GGe     p[GG] p[GG]<.05       HFe     p[HF] p[HF]<.05
## 2    GVS 0.9190022 0.1908998           0.9782075 0.1887949
```

```
## Warning: Removed 1 rows containing non-finite values (stat_ydensity).
```

```
## $ANOVA
##   Effect DFn DFd         F         p p<.05         ges
## 2    GVS   2  58 0.8268679 0.4425069       0.007990474
## 
## $`Mauchly's Test for Sphericity`
##   Effect         W          p p<.05
## 2    GVS 0.8370644 0.08291329      
## 
## $`Sphericity Corrections`
##   Effect       GGe     p[GG] p[GG]<.05       HFe     p[HF] p[HF]<.05
## 2    GVS 0.8598928 0.4272704           0.9089659 0.4328968
```

```
## $ANOVA
##   Effect DFn DFd       F         p p<.05        ges
## 2    GVS   2  58 1.14166 0.3263578       0.01542648
## 
## $`Mauchly's Test for Sphericity`
##   Effect         W           p p<.05
## 2    GVS 0.6620335 0.003106848     *
## 
## $`Sphericity Corrections`
##   Effect       GGe     p[GG] p[GG]<.05       HFe     p[HF] p[HF]<.05
## 2    GVS 0.7474029 0.3149339           0.7788379 0.3167431
```

```
## $ANOVA
##   Effect DFn DFd         F         p p<.05         ges
## 2    GVS   2  58 0.4060074 0.6681841       0.005684989
## 
## $`Mauchly's Test for Sphericity`
##   Effect         W         p p<.05
## 2    GVS 0.9197698 0.3101045      
## 
## $`Sphericity Corrections`
##   Effect       GGe     p[GG] p[GG]<.05       HFe     p[HF] p[HF]<.05
## 2    GVS 0.9257286 0.6525984           0.9861251 0.6653642
```

```
## Warning: Removed 1 rows containing non-finite values (stat_ydensity).
```

```
## $ANOVA
##   Effect DFn DFd           F         p p<.05          ges
## 2    GVS   2  58 0.008147732 0.9918865       0.0001280792
## 
## $`Mauchly's Test for Sphericity`
##   Effect       W         p p<.05
## 2    GVS 0.96668 0.6222402      
## 
## $`Sphericity Corrections`
##   Effect       GGe     p[GG] p[GG]<.05      HFe     p[HF] p[HF]<.05
## 2    GVS 0.9677544 0.9907015           1.035772 0.9918865
```

```
## $ANOVA
##   Effect DFn DFd        F         p p<.05         ges
## 2    GVS   2  58 1.033076 0.3623658       0.007625854
## 
## $`Mauchly's Test for Sphericity`
##   Effect         W          p p<.05
## 2    GVS 0.8193771 0.06148565      
## 
## $`Sphericity Corrections`
##   Effect       GGe     p[GG] p[GG]<.05      HFe     p[HF] p[HF]<.05
## 2    GVS 0.8470105 0.3529371           0.893955 0.3560877
```

```
## $ANOVA
##   Effect DFn DFd         F         p p<.05         ges
## 2    GVS   2  58 0.6450588 0.5283537       0.002289968
## 
## $`Mauchly's Test for Sphericity`
##   Effect         W           p p<.05
## 2    GVS 0.7178124 0.009641691     *
## 
## $`Sphericity Corrections`
##   Effect       GGe     p[GG] p[GG]<.05       HFe     p[HF] p[HF]<.05
## 2    GVS 0.7799171 0.4922444           0.8162309 0.4988312
```

To summarise: 1) variability is either very high, depending on subject-specific response “bias”, or very low, with results clustered around “I felt nothing”; 2) quite all questions actually support the absence of a (at least explicitly) perceived difference between conditions, allowing us to conclude that the SHAM condition effectively controlled for unspecific effects of brain stimulation such as tingling or itching (or discomfort in general); 3) there was a very small effect for the question “I felt my body rotating”: subjects more often responded clockwise for Right-Anodal, counterclockwise for Left-Anodal. This is probably driven by a subset of participants, because results are generally clustered around the center of the line in the majority of cases. This is a specific vestibular effect though, and values for this item were entered into exploratory analyses exploiting correlations.

## Appendix

This is the function to summarise data (`summarySEwithin`):

```
## Summarizes data.
## Gives count, mean, standard deviation, standard error of the mean, and confidence interval (default 95%).
##   data: a data frame.
##   measurevar: the name of a column that contains the variable to be summariezed
##   groupvars: a vector containing names of columns that contain grouping variables
##   na.rm: a boolean that indicates whether to ignore NA's
##   conf.interval: the percent range of the confidence interval (default is 95%)
summarySE <- function(data=NULL, measurevar, groupvars=NULL, na.rm=FALSE,
                      conf.interval=.95, .drop=TRUE) {
  library(plyr)
  
  # New version of length which can handle NA's: if na.rm==T, don't count them
  length2 <- function (x, na.rm=FALSE) {
    if (na.rm) sum(!is.na(x))
    else       length(x)
  }
  
  # This does the summary. For each group's data frame, return a vector with
  # N, mean, and sd
  datac <- ddply(data, groupvars, .drop=.drop,
                 .fun = function(xx, col) {
                   c(N    = length2(xx[[col]], na.rm=na.rm),
                     mean = mean   (xx[[col]], na.rm=na.rm),
                     sd   = sd     (xx[[col]], na.rm=na.rm)
                   )
                 },
                 measurevar
  )
  
  # Rename the "mean" column    
  datac <- plyr::rename(datac, c(mean = measurevar))
  
  datac$se <- datac$sd / sqrt(datac$N)  # Calculate standard error of the mean
  
  # Confidence interval multiplier for standard error
  # Calculate t-statistic for confidence interval: 
  # e.g., if conf.interval is .95, use .975 (above/below), and use df=N-1
  ciMult <- qt(conf.interval/2 + .5, datac$N-1)
  datac$ci <- datac$se * ciMult
  
  return(datac)
}


## Norms the data within specified groups in a data frame; it normalizes each
## subject (identified by idvar) so that they have the same mean, within each group
## specified by betweenvars.
##   data: a data frame.
##   idvar: the name of a column that identifies each subject (or matched subjects)
##   measurevar: the name of a column that contains the variable to be summariezed
##   betweenvars: a vector containing names of columns that are between-subjects variables
##   na.rm: a boolean that indicates whether to ignore NA's
normDataWithin <- function(data=NULL, idvar, measurevar, betweenvars=NULL,
                           na.rm=FALSE, .drop=TRUE) {
  library(plyr)
  
  # Measure var on left, idvar + between vars on right of formula.
  data.subjMean <- ddply(data, c(idvar, betweenvars), .drop=.drop,
                         .fun = function(xx, col, na.rm) {
                           c(subjMean = mean(xx[,col], na.rm=na.rm))
                         },
                         measurevar,
                         na.rm
  )
  
  # Put the subject means with original data
  data <- merge(data, data.subjMean)
  
  # Get the normalized data in a new column
  measureNormedVar <- paste(measurevar, "_norm", sep="")
  data[,measureNormedVar] <- data[,measurevar] - data[,"subjMean"] +
    mean(data[,measurevar], na.rm=na.rm)
  
  # Remove this subject mean column
  data$subjMean <- NULL
  
  return(data)
}


## Retrieved here: http://www.cookbook-r.com/Graphs/Plotting_means_and_error_bars_(ggplot2)/
## Summarizes data, handling within-subjects variables by removing inter-subject variability.
## It will still work if there are no within-S variables.
## Gives count, un-normed mean, normed mean (with same between-group mean),
##   standard deviation, standard error of the mean, and confidence interval.
## If there are within-subject variables, calculate adjusted values using method from Morey (2008).
##   data: a data frame.
##   measurevar: the name of a column that contains the variable to be summariezed
##   betweenvars: a vector containing names of columns that are between-subjects variables
##   withinvars: a vector containing names of columns that are within-subjects variables
##   idvar: the name of a column that identifies each subject (or matched subjects)
##   na.rm: a boolean that indicates whether to ignore NA's
##   conf.interval: the percent range of the confidence interval (default is 95%)
summarySEwithin <- function(data=NULL, measurevar, betweenvars=NULL, withinvars=NULL,
                            idvar=NULL, na.rm=FALSE, conf.interval=.95, .drop=TRUE) {
  
  # Ensure that the betweenvars and withinvars are factors
  factorvars <- vapply(data[, c(betweenvars, withinvars), drop=FALSE],
                       FUN=is.factor, FUN.VALUE=logical(1))
  
  if (!all(factorvars)) {
    nonfactorvars <- names(factorvars)[!factorvars]
    message("Automatically converting the following non-factors to factors: ",
            paste(nonfactorvars, collapse = ", "))
    data[nonfactorvars] <- lapply(data[nonfactorvars], factor)
  }
  
  # Get the means from the un-normed data
  datac <- summarySE(data, measurevar, groupvars=c(betweenvars, withinvars),
                     na.rm=na.rm, conf.interval=conf.interval, .drop=.drop)
  
  # Drop all the unused columns (these will be calculated with normed data)
  datac$sd <- NULL
  datac$se <- NULL
  datac$ci <- NULL
  
  # Norm each subject's data
  ndata <- normDataWithin(data, idvar, measurevar, betweenvars, na.rm, .drop=.drop)
  
  # This is the name of the new column
  measurevar_n <- paste(measurevar, "_norm", sep="")
  
  # Collapse the normed data - now we can treat between and within vars the same
  ndatac <- summarySE(ndata, measurevar_n, groupvars=c(betweenvars, withinvars),
                      na.rm=na.rm, conf.interval=conf.interval, .drop=.drop)
  
  # Apply correction from Morey (2008) to the standard error and confidence interval
  #  Get the product of the number of conditions of within-S variables
  nWithinGroups    <- prod(vapply(ndatac[,withinvars, drop=FALSE], FUN=nlevels,
                                  FUN.VALUE=numeric(1)))
  correctionFactor <- sqrt( nWithinGroups / (nWithinGroups-1) )
  
  # Apply the correction factor
  ndatac$sd <- ndatac$sd * correctionFactor
  ndatac$se <- ndatac$se * correctionFactor
  ndatac$ci <- ndatac$ci * correctionFactor
  
  # Combine the un-normed means with the normed results
  merge(datac, ndatac)
}
```
