# Supplementary material for: Probing the role of the vestibular system in motivation and reward-based attention
Source: Cortex. 2018 Jun;103:82–99. doi: 10.1016/j.cortex.2018.02.009 (PMC6002611; doi:10.1016/j.cortex.2018.02.009)
Supplement: Supplementary file 3 [file mmc3.zip › cortex_2252_mmc3.html]

S3: ART task, reaction times


# S3: ART task, reaction times

#### *Elvio Blini INSERM U1028, ImpAct team, CRNL, and University of Lyon elvio.blini@gmail.com*

#### *30 November 2017*

# Attention and Reward Task (ART)

This is the main task administered to subjects. Each trial started with a fixation cross lasting 750ms. Then, one of the two lateral placeholders, either the left or right one, changed colour for 100 ms (cue). After additional 600 ms (on average) a target - either square or circle - was presented either on the left or right side of the screen. The position of the cue was not predictive of the side of target presentation (50%). However, the color of the cue predicted (100%) the amount of points that the subject could earn for a correct and fast response. A feeback was presented at the end of each trial reporting whether the response was correct, incorrect, too fast, or too slow; the number of points rewarded for the current trial; the overall gain since the beginning of the task.

Time course of a typical trial of the ART.

There’s a whealth on information we can extract from this task. We expected, to begin with, a main effect of Reward, suggesting that our manipulation effectively modulated motivation. Then, we sought to look for the interaction of Reward and Validity. In theory, motivational and spatial cues might interact, and the validity gain/inhibition of return commonly observed in Posner-like tasks might be modulated consequently. With this respect, studies in literature have been inconsistent, though the specific paradigm employed changed sensibly as well. In a study very similar to ours, high rewards were found to enhance inhibition of return specifically (Bucker and Theeuwes, 2014). However, the key interests of our study were the possible interactions with a vestibular stimulation (GVS, see the companion paper). The first question was whether GVS modulates sensitivity to rewards. With this respect, we wondered whether reward-based performance boosts could be further modulated by GVS, either in the sense of an abolishment, or in the sense of a further enhancement (bi-directional hypotheses). Finally, a three-way interaction GVS by Reward by Validity was also predicted, showing that GVS might affect the interplay between motivation and spatial attention (again, please refer to the main text for the rationale).

**This document presents results for reaction times, a companion file, S4, reports the results for accuracy.**

## Preliminary setup

It’s sometimes good to clean the current environment to avoid conflicts. You can do it with `rm(list=ls())` (but be sure everything is properly saved for future use).

In order to run this script we need a few packages available on CRAN. You might need to install them first, e.g. by typing `install.packages("BayesFactor")` in the console.

```
#list packages
packages= c("plyr", "magrittr", "tidyverse", "BayesFactor", "lme4", "multcomp",
            "gridExtra", "ez" , "lsmeans", "retimes")

#load them
lapply(packages, require, character.only= T)
```

For package `afex` I’m using the developer version on github (you will need to install `devtools`).

```
#devtools::install_github("singmann/afex@master")
library(afex)
```

For reproducibility, here’s details of the system on which the script was tested and a seed for random numbers:

```
set.seed(1)

sessionInfo()
```

```
## R version 3.4.2 (2017-09-28)
## Platform: x86_64-w64-mingw32/x64 (64-bit)
## Running under: Windows 10 x64 (build 15063)
## 
## Matrix products: default
## 
## locale:
## [1] LC_COLLATE=English_United Kingdom.1252 
## [2] LC_CTYPE=English_United Kingdom.1252   
## [3] LC_MONETARY=English_United Kingdom.1252
## [4] LC_NUMERIC=C                           
## [5] LC_TIME=English_United Kingdom.1252    
## 
## attached base packages:
## [1] stats     graphics  grDevices utils     datasets  methods   base     
## 
## other attached packages:
##  [1] afex_0.18-0          retimes_0.1-2        lsmeans_2.27-2      
##  [4] estimability_1.2     ez_4.4-0             gridExtra_2.3       
##  [7] multcomp_1.4-7       TH.data_1.0-8        MASS_7.3-47         
## [10] survival_2.41-3      mvtnorm_1.0-6        lme4_1.1-14         
## [13] BayesFactor_0.9.12-2 Matrix_1.2-11        coda_0.19-1         
## [16] dplyr_0.7.4          purrr_0.2.3          readr_1.1.1         
## [19] tidyr_0.7.1          tibble_1.3.4         ggplot2_2.2.1       
## [22] tidyverse_1.1.1      magrittr_1.5         plyr_1.8.4          
## [25] printr_0.1          
## 
## loaded via a namespace (and not attached):
##  [1] nlme_3.1-131        pbkrtest_0.4-7      lubridate_1.6.0    
##  [4] RColorBrewer_1.1-2  httr_1.3.1          rprojroot_1.2      
##  [7] tools_3.4.2         backports_1.1.1     R6_2.2.2           
## [10] rpart_4.1-11        Hmisc_4.0-3         lazyeval_0.2.0     
## [13] mgcv_1.8-20         colorspace_1.3-2    nnet_7.3-12        
## [16] mnormt_1.5-5        compiler_3.4.2      rvest_0.3.2        
## [19] quantreg_5.33       htmlTable_1.9       SparseM_1.77       
## [22] xml2_1.1.1          sandwich_2.4-0      checkmate_1.8.4    
## [25] scales_0.5.0        psych_1.7.8         pbapply_1.3-3      
## [28] stringr_1.2.0       digest_0.6.12       foreign_0.8-69     
## [31] minqa_1.2.4         rmarkdown_1.7       base64enc_0.1-3    
## [34] pkgconfig_2.0.1     htmltools_0.3.6     htmlwidgets_0.9    
## [37] rlang_0.1.2         readxl_1.0.0        bindr_0.1          
## [40] zoo_1.8-0           jsonlite_1.5        gtools_3.5.0       
## [43] acepack_1.4.1       car_2.1-5           modeltools_0.2-21  
## [46] Formula_1.2-2       Rcpp_0.12.13        munsell_0.4.3      
## [49] stringi_1.1.5       yaml_2.1.14         grid_3.4.2         
## [52] parallel_3.4.2      forcats_0.2.0       lattice_0.20-35    
## [55] haven_1.1.0         splines_3.4.2       hms_0.3            
## [58] knitr_1.17          reshape2_1.4.2      codetools_0.2-15   
## [61] stats4_3.4.2        glue_1.1.1          evaluate_0.10.1    
## [64] latticeExtra_0.6-28 data.table_1.10.4   modelr_0.1.1       
## [67] nloptr_1.0.4        MatrixModels_0.4-1  cellranger_1.1.0   
## [70] gtable_0.2.0        assertthat_0.2.0    coin_1.2-1         
## [73] xtable_1.8-2        broom_0.4.2         lmerTest_2.0-33    
## [76] bindrcpp_0.2        cluster_2.0.6
```

Thanks to the function retrieved here, not displayed, the following hyperlinks download the Rdata files:

That can be loaded then with:

```
load("ART data.RData")
```

Now all relevant variables are stored in the `ART` data.frame, that you can navigate and explore with the usual commands, e.g. `str(ART)`.

## Useful functions

This is a minimal (shared by all plots) list of graphic attributes for ggplot:

```
#ggplot defaults
commonTheme = list(theme_bw(),
                   theme(text = element_text(size=18, face="bold"),
                         axis.text = element_text(size=16, face="bold", color= "black"),
                         plot.margin = unit(c(1,1,1,1), "cm")))
```

Then (ONLY SHOWN IN THE BOTTOM PART OF THIS SCRIPT) we have a convenient summarising function that produces means and within subjects SEM as in Morey (2008). I retrieved it here: link

## Preprocessing

A bunch of variables is to declare (e.g., numbers into factors). Then, for the sake of clarity, levels are renamed when helpful.

```
#declare
ART$Subject= as.factor(ART$Subject)

if(sum(levels(as.factor(ART$Reward))== c("0", "2", "10"))==3){
  ART$Reward= factor(ART$Reward, labels = c("None", "Small", "High"))} else 
    (warning("levels unordered? Check"))

if(sum(levels(as.factor(ART$GVS))== c("A", "B", "C"))==3){
  ART$GVS= factor(ART$GVS, labels = c("SHAM", "Left-Anodal", "Right-Anodal"))
  ART$GVS= relevel(ART$GVS, "Left-Anodal")} else (warning("levels unordered? Check"))
```

We must drop a few subjects ( :( ). Reasons are in the comments.

```
#subject 1 to replace for a bug in the script, my fault, :(
ART= ART[!ART$Subject== 1, ] #replaced with subject 51

#subject 10 didn't show up after first session, >:(
ART= ART[!ART$Subject== 10, ] #replaced with subject 60

ART$Subject= factor(ART$Subject)
```

We create a “Block” variable.

```
#insert variable "block"
ART$Block= ifelse(ART$Trial< 145, 1, 
                  ifelse(ART$Trial> 288, 3, 2)) %>% as.factor
```

A good response (valid) was defined as being correct, slower than 100ms, and faster than 500ms.

```
#exclusion criteria applies to valid responses
ART$Valid_Response= ifelse(ART$correct== 1 &
                           ART$response_time> 100 &
                           ART$response_time< 500, 1, 0)
```

We can start with our quality checks! First, subjects with less that 60% of trials per cell remaining have to be discarded:

```
#must be >60% for each subject
ddply(ART, "Subject", summarise, 
      acc= mean(Valid_Response)) %$% acc %>%
      sapply(function(x) x<0.6)
```

```
##  [1] FALSE FALSE FALSE FALSE FALSE FALSE FALSE FALSE FALSE FALSE FALSE
## [12] FALSE FALSE FALSE FALSE FALSE FALSE FALSE FALSE FALSE FALSE FALSE
## [23] FALSE FALSE FALSE FALSE FALSE FALSE FALSE FALSE
```

Second, the threshold was set to 30% when considering the GVS by Reward by Validity cells:

```
#must be >30% for each cell in the triple interaction
ddply(ART, c("GVS", "Reward", "Validity", "Subject"), summarise, 
      acc= mean(Valid_Response)) %>% 
      with(tapply(acc, Subject, function(x)(x<0.3))) %>%
      lapply(function(x) (sum(x)))>0
```

```
##     2     3     4     5     6     7     8     9    11    12    13    14 
## FALSE FALSE FALSE FALSE FALSE FALSE FALSE FALSE FALSE FALSE FALSE FALSE 
##    15    16    17    18    19    20    21    22    23    24    25    26 
## FALSE FALSE FALSE FALSE FALSE FALSE FALSE FALSE FALSE FALSE FALSE FALSE 
##    27    28    29    30    51    60 
## FALSE FALSE FALSE FALSE FALSE FALSE
```

Thus, so far no replacement is necessary! We had two criteria to proceed with analyses for accuracy (the objective was to avoid ceiling effects). The group mean must be less than 95%:

```
#group mean >95%
ddply(ART, "Subject", summarise, 
      acc= mean(correct)) %$% print(mean(acc))<0.95
```

```
## [1] 0.9420782
```

```
## [1] TRUE
```

Very close… Then, less than half of the subjects must present >95% of correct responses:

```
#less than half of subjects has >95%
ddply(ART, "Subject", summarise, 
      acc= mean(correct)) %$% print(mean(acc<0.95))>0.5
```

```
## [1] 0.5666667
```

```
## [1] TRUE
```

As it is now, we can proceed with analyses! (Though a few subjects still present 100% accuracy in some cells, I wonder whether this will be problematic when fitting mixed models…).

We can save a separate dataframe with wrong responses, this way we will work with reaction times for good responses only:

```
#save data for follow-up analyses and plots (before excluding wrong responses)
ART.accuracy= ART

#exclude bad responses
ART= ART[ART$Valid_Response==1, ]
```

Finally, another criterion for subjects’ replacement was the absence of extremely deviant reaction times in a subset of cells:

```
#our check was for cells in the GVS by Reward by Validity interaction for each subject
#a subject is replaced if exceeds +-3 standard deviations from his/her mean
ddply(ART, c("GVS", "Reward", "Validity", "Subject"), summarise, 
      rts= mean(response_time)) %>%
      with(tapply(rts, Subject, scale)) %>% 
      lapply(function(x) (ifelse(abs(as.numeric(x))>3, 1, 0))) %>%
      lapply(function(x) (sum(x)))>0
```

```
##     2     3     4     5     6     7     8     9    11    12    13    14 
## FALSE FALSE FALSE FALSE FALSE FALSE FALSE FALSE FALSE FALSE FALSE FALSE 
##    15    16    17    18    19    20    21    22    23    24    25    26 
## FALSE FALSE FALSE FALSE FALSE FALSE FALSE FALSE FALSE FALSE FALSE FALSE 
##    27    28    29    30    51    60 
## FALSE FALSE FALSE FALSE FALSE FALSE
```

All good!

## Analyses

### RTs

The main procedure on which we rely is based on mixed linear regression models, explained below and in the manuscript.

We can start with model selection procedure. The general strategy is to evaluate beforehand the random effects that increase model fitting, as to reach a parsimonious solution (i.e. supported by data). We create several different (nested) models and evaluate them against a simpler reference one through likelihood ratio tests (LRT). This holds for both random and fixed effects testing.

The simplest model to start with only includes the random intercept for Subjects (baseline level). We then start testing random slopes one by one, following this order:

1. GVS
2. Reward
3. Validity
4. Side
5. Block
6. Correct Response (Shape) - which covaries with the motor effector used in order to provide a response (i.e. index or middle figer)

Each random slope - that informs about variability in performance across levels of a factor, e.g. differences in experimental manipulations across subjects - will be retained in the model if the LRT is proven significant. Following evaluations will be made with reference models that include this slope. For example, if GVS improves model fit as random slope, StartingSide will be evaluated against the model including it. As a second step we introduce interactions for all combination of slopes proven significant (this is to respect marginality, thus include high-order terms together with their lower-order ones).

Fixed effects testing will use a similar (type 2) approach.

(To avoid verbosity, only the p value is shown when selecting random effects).

This is the starting model:

```
rtmod0= lmer(response_time ~ (1|Subject), data= ART, REML=T,
             control=lmerControl(optimizer="bobyqa"))
```

Note that: we asked for the bobyqa algorithm, which handles convergence problems very well; we asked for restricted maximum likelihood fitting in this stage (we will switch to maximum likelihood for fixed effects). The first slope to be evaluated is for GVS:

```
rtmod0a= lmer(response_time ~ (1+GVS|Subject), data= ART, REML=T,
             control=lmerControl(optimizer="bobyqa"))
```

We can test for its role with a LRT:

```
anova(rtmod0, rtmod0a, refit= F)
```

|  | Df | AIC | BIC | logLik | deviance | Chisq | Chi Df | Pr(>Chisq) |
| --- | --- | --- | --- | --- | --- | --- | --- | --- |
| rtmod0 | 3 | 369063.3 | 369088.7 | -184528.7 | 369057.3 | NA | NA | NA |
| rtmod0a | 8 | 368661.8 | 368729.4 | -184322.9 | 368645.8 | 411.5198 | 5 | 0 |

It is very important (also look at AIC and BIC criteria!). We move to the following slopes:

```
rtmod0b= lmer(response_time ~ (1+GVS+Reward|Subject), data= ART, REML=T,
             control=lmerControl(optimizer="bobyqa"))
anova(rtmod0a, rtmod0b, refit= F)$`Pr(>Chisq)`[2] #retained
```

```
## [1] 6.253952e-28
```

```
rtmod0c= lmer(response_time ~ (1+GVS+Reward+Validity|Subject), data= ART, REML=T,
             control=lmerControl(optimizer="bobyqa"))
anova(rtmod0b, rtmod0c, refit= F)$`Pr(>Chisq)`[2] #retained
```

```
## [1] 1.216935e-45
```

```
rtmod0d= lmer(response_time ~ (1+GVS+Reward+Validity+Side|Subject), data= ART, REML=T,
             control=lmerControl(optimizer="bobyqa", optCtrl=list(maxfun=10^20)))
# Warning messages:
# 1: In (function (par, fn, lower = -Inf, upper = Inf, control = list(),  :
#   NAs introduced by coercion to integer range
# 2: In optwrap(optimizer, devfun, getStart(start, rho$lower, rho$pp),  :
#   convergence code 1 from bobyqa: bobyqa -- maximum number of function evaluations exceeded
# 3: In checkConv(attr(opt, "derivs"), opt$par, ctrl = control$checkConv,  :
#   unable to evaluate scaled gradient
# 4: In checkConv(attr(opt, "derivs"), opt$par, ctrl = control$checkConv,  :
#   Model failed to converge: degenerate  Hessian with 7 negative eigenvalues
  
rtmod0e= lmer(response_time ~ (1+GVS+Reward+Validity+Block|Subject), data= ART, REML=T,
             control=lmerControl(optimizer="bobyqa", optCtrl=list(maxfun=10^20)))

# Warning messages:
# 1: In (function (par, fn, lower = -Inf, upper = Inf, control = list(),  :
#   NAs introduced by coercion to integer range
# 2: In optwrap(optimizer, devfun, getStart(start, rho$lower, rho$pp),  :
#   convergence code 1 from bobyqa: bobyqa -- maximum number of function evaluations exceeded
# 3: In checkConv(attr(opt, "derivs"), opt$par, ctrl = control$checkConv,  :
#   unable to evaluate scaled gradient
# 4: In checkConv(attr(opt, "derivs"), opt$par, ctrl = control$checkConv,  :
#   Model failed to converge: degenerate  Hessian with 9 negative eigenvalues
  
rtmod0f= lmer(response_time ~ (1+GVS+Reward+Validity+Shape|Subject), data= ART, REML=T,
             control=lmerControl(optimizer="bobyqa", optCtrl=list(maxfun=10^20)))

# Warning messages:
# 1: In (function (par, fn, lower = -Inf, upper = Inf, control = list(),  :
#   NAs introduced by coercion to integer range
# 2: In optwrap(optimizer, devfun, getStart(start, rho$lower, rho$pp),  :
#   convergence code 1 from bobyqa: bobyqa -- maximum number of function evaluations exceeded
# 3: In checkConv(attr(opt, "derivs"), opt$par, ctrl = control$checkConv,  :
#   unable to evaluate scaled gradient
# 4: In checkConv(attr(opt, "derivs"), opt$par, ctrl = control$checkConv,  :
#   Model failed to converge: degenerate  Hessian with 8 negative eigenvalues
```

Next: two-way interactions. (GVS, Reward, Validity).

```
rtmod0g= lmer(response_time ~ (1+GVS*Reward+Validity|Subject), data= ART, REML=T,
             control=lmerControl(optimizer="bobyqa", optCtrl=list(maxfun=10^20)))

# Warning messages:
# 1: In (function (par, fn, lower = -Inf, upper = Inf, control = list(),  :
#   NAs introduced by coercion to integer range
# 2: In optwrap(optimizer, devfun, getStart(start, rho$lower, rho$pp),  :
#   convergence code 1 from bobyqa: bobyqa -- maximum number of function evaluations exceeded
# 3: In checkConv(attr(opt, "derivs"), opt$par, ctrl = control$checkConv,  :
#   unable to evaluate scaled gradient
# 4: In checkConv(attr(opt, "derivs"), opt$par, ctrl = control$checkConv,  :
#   Model failed to converge: degenerate  Hessian with 17 negative eigenvalues
   
rtmod0h= lmer(response_time ~ (1+GVS*Validity+Reward|Subject), data= ART, REML=T,
             control=lmerControl(optimizer="bobyqa", optCtrl=list(maxfun=10^20)))

# Warning messages:
# 1: In (function (par, fn, lower = -Inf, upper = Inf, control = list(),  :
#   NAs introduced by coercion to integer range
# 2: In optwrap(optimizer, devfun, getStart(start, rho$lower, rho$pp),  :
#   convergence code 1 from bobyqa: bobyqa -- maximum number of function evaluations exceeded
# 3: In checkConv(attr(opt, "derivs"), opt$par, ctrl = control$checkConv,  :
#   unable to evaluate scaled gradient
# 4: In checkConv(attr(opt, "derivs"), opt$par, ctrl = control$checkConv,  :
#   Model failed to converge: degenerate  Hessian with 10 negative eigenvalues
  
rtmod0i= lmer(response_time ~ (1+GVS+Reward*Validity|Subject), data= ART, REML=T,
             control=lmerControl(optimizer="bobyqa", optCtrl=list(maxfun=10^20)))

# Warning messages:
# 1: In (function (par, fn, lower = -Inf, upper = Inf, control = list(),  :
#   NAs introduced by coercion to integer range
# 2: In optwrap(optimizer, devfun, getStart(start, rho$lower, rho$pp),  :
#   convergence code 1 from bobyqa: bobyqa -- maximum number of function evaluations exceeded
# 3: In checkConv(attr(opt, "derivs"), opt$par, ctrl = control$checkConv,  :
#   unable to evaluate scaled gradient
# 4: In checkConv(attr(opt, "derivs"), opt$par, ctrl = control$checkConv,  :
#   Model failed to converge: degenerate  Hessian with 9 negative eigenvalues
```

We have finally chosen our model, let’s switch to fixed effects exploiting the `afex::mixed` function. The all\_fit parameter tests several different optimizers to get rid of convergence problems if possible.

```
rts_models= mixed(response_time ~ GVS*Reward*Validity*Side*Block +
                 (1+GVS+Reward+Validity|Subject),
               expand_re = F, all_fit = T, data= ART, method= "LRT",  type= "2",
               control= lmerControl(optimizer="bobyqa", optCtrl=list(maxfun=10^20)))
```

A lot of optimizers have been tested. This took ~36h on an average machine (4Gb RAM, 2.8 GHz processor). Let’s check for any convergence problem left:

```
lapply(rts_models$restricted_models, function(x) x@optinfo$warnings)
```

```
## $GVS
## list()
## 
## $Reward
## list()
## 
## $Validity
## list()
## 
## $Side
## list()
## 
## $Block
## list()
## 
## $`GVS:Reward`
## list()
## 
## $`GVS:Validity`
## list()
## 
## $`Reward:Validity`
## list()
## 
## $`GVS:Side`
## list()
## 
## $`Reward:Side`
## list()
## 
## $`Validity:Side`
## list()
## 
## $`GVS:Block`
## list()
## 
## $`Reward:Block`
## list()
## 
## $`Validity:Block`
## list()
## 
## $`Side:Block`
## list()
## 
## $`GVS:Reward:Validity`
## list()
## 
## $`GVS:Reward:Side`
## list()
## 
## $`GVS:Validity:Side`
## list()
## 
## $`Reward:Validity:Side`
## list()
## 
## $`GVS:Reward:Block`
## list()
## 
## $`GVS:Validity:Block`
## list()
## 
## $`Reward:Validity:Block`
## list()
## 
## $`GVS:Side:Block`
## list()
## 
## $`Reward:Side:Block`
## list()
## 
## $`Validity:Side:Block`
## list()
## 
## $`GVS:Reward:Validity:Side`
## list()
## 
## $`GVS:Reward:Validity:Block`
## list()
## 
## $`GVS:Reward:Side:Block`
## list()
## 
## $`GVS:Validity:Side:Block`
## list()
## 
## $`Reward:Validity:Side:Block`
## list()
## 
## $`GVS:Reward:Validity:Side:Block`
## list()
```

```
lapply(rts_models$restricted_models, function(x) x@optinfo$conv) %>% unlist
```

```
##                            GVS.opt                         Reward.opt 
##                                  0                                  0 
##                       Validity.opt                           Side.opt 
##                                  0                                  0 
##                          Block.opt                     GVS:Reward.opt 
##                                  0                                  0 
##                   GVS:Validity.opt                Reward:Validity.opt 
##                                  0                                  0 
##                       GVS:Side.opt                    Reward:Side.opt 
##                                  0                                  0 
##                  Validity:Side.opt                      GVS:Block.opt 
##                                  0                                  0 
##                   Reward:Block.opt                 Validity:Block.opt 
##                                  0                                  0 
##                     Side:Block.opt            GVS:Reward:Validity.opt 
##                                  0                                  0 
##                GVS:Reward:Side.opt              GVS:Validity:Side.opt 
##                                  0                                  0 
##           Reward:Validity:Side.opt               GVS:Reward:Block.opt 
##                                  0                                  0 
##             GVS:Validity:Block.opt          Reward:Validity:Block.opt 
##                                  0                                  0 
##                 GVS:Side:Block.opt              Reward:Side:Block.opt 
##                                  0                                  0 
##            Validity:Side:Block.opt       GVS:Reward:Validity:Side.opt 
##                                  0                                  0 
##      GVS:Reward:Validity:Block.opt          GVS:Reward:Side:Block.opt 
##                                  0                                  0 
##        GVS:Validity:Side:Block.opt     Reward:Validity:Side:Block.opt 
##                                  0                                  0 
## GVS:Reward:Validity:Side:Block.opt 
##                                  0
```

```
sapply(rts_models$restricted_models, inherits, "try-error")
```

```
##                            GVS                         Reward 
##                          FALSE                          FALSE 
##                       Validity                           Side 
##                          FALSE                          FALSE 
##                          Block                     GVS:Reward 
##                          FALSE                          FALSE 
##                   GVS:Validity                Reward:Validity 
##                          FALSE                          FALSE 
##                       GVS:Side                    Reward:Side 
##                          FALSE                          FALSE 
##                  Validity:Side                      GVS:Block 
##                          FALSE                          FALSE 
##                   Reward:Block                 Validity:Block 
##                          FALSE                          FALSE 
##                     Side:Block            GVS:Reward:Validity 
##                          FALSE                          FALSE 
##                GVS:Reward:Side              GVS:Validity:Side 
##                          FALSE                          FALSE 
##           Reward:Validity:Side               GVS:Reward:Block 
##                          FALSE                          FALSE 
##             GVS:Validity:Block          Reward:Validity:Block 
##                          FALSE                          FALSE 
##                 GVS:Side:Block              Reward:Side:Block 
##                          FALSE                          FALSE 
##            Validity:Side:Block       GVS:Reward:Validity:Side 
##                          FALSE                          FALSE 
##      GVS:Reward:Validity:Block          GVS:Reward:Side:Block 
##                          FALSE                          FALSE 
##        GVS:Validity:Side:Block     Reward:Validity:Side:Block 
##                          FALSE                          FALSE 
## GVS:Reward:Validity:Side:Block 
##                          FALSE
```

```
sapply(rts_models$restricted_models, function(x) {
  with(x@optinfo$derivs, solve(Hessian,gradient)) %>% abs %>% max %>% return})
```

```
##                            GVS                         Reward 
##                   4.587378e-06                   5.816613e-06 
##                       Validity                           Side 
##                   3.832172e-06                   2.705774e-06 
##                          Block                     GVS:Reward 
##                   7.761524e-06                   1.924065e-06 
##                   GVS:Validity                Reward:Validity 
##                   3.709230e-06                   2.486460e-06 
##                       GVS:Side                    Reward:Side 
##                   8.086966e-06                   2.655817e-06 
##                  Validity:Side                      GVS:Block 
##                   2.742641e-06                   7.040005e-06 
##                   Reward:Block                 Validity:Block 
##                   2.223205e-06                   2.337322e-06 
##                     Side:Block            GVS:Reward:Validity 
##                   7.480542e-06                   3.446221e-06 
##                GVS:Reward:Side              GVS:Validity:Side 
##                   2.286542e-06                   3.033571e-06 
##           Reward:Validity:Side               GVS:Reward:Block 
##                   2.161275e-06                   3.705640e-06 
##             GVS:Validity:Block          Reward:Validity:Block 
##                   1.310998e-06                   7.021721e-06 
##                 GVS:Side:Block              Reward:Side:Block 
##                   4.815285e-06                   5.657811e-06 
##            Validity:Side:Block       GVS:Reward:Validity:Side 
##                   3.263644e-06                   2.152666e-06 
##      GVS:Reward:Validity:Block          GVS:Reward:Side:Block 
##                   5.031575e-06                   7.792428e-06 
##        GVS:Validity:Side:Block     Reward:Validity:Side:Block 
##                   6.669258e-06                   4.159371e-06 
## GVS:Reward:Validity:Side:Block 
##                   5.782350e-06
```

But all controls, and especially achieved tolerance, are satisfactory…

We can display results with:

```
rts_models %>% nice
```

| Effect | df | Chisq | p.value |
| --- | --- | --- | --- |
| GVS | 2 | 1.90 | .39 |
| Reward | 2 | 29.25 \*\*\* | <.0001 |
| Validity | 1 | 9.23 \*\* | .002 |
| Side | 1 | 55.24 \*\*\* | <.0001 |
| Block | 2 | 12.63 \*\* | .002 |
| GVS:Reward | 4 | 19.11 \*\*\* | .0007 |
| GVS:Validity | 2 | 2.18 | .34 |
| Reward:Validity | 2 | 3.69 | .16 |
| GVS:Side | 2 | 3.37 | .19 |
| Reward:Side | 2 | 1.05 | .59 |
| Validity:Side | 1 | 5.03 \* | .02 |
| GVS:Block | 4 | 23.63 \*\*\* | <.0001 |
| Reward:Block | 4 | 12.71 \* | .01 |
| Validity:Block | 2 | 9.80 \*\* | .007 |
| Side:Block | 2 | 0.27 | .87 |
| GVS:Reward:Validity | 4 | 3.98 | .41 |
| GVS:Reward:Side | 4 | 3.73 | .44 |
| GVS:Validity:Side | 2 | 1.70 | .43 |
| Reward:Validity:Side | 2 | 4.82 + | .09 |
| GVS:Reward:Block | 8 | 18.43 \* | .02 |
| GVS:Validity:Block | 4 | 1.63 | .80 |
| Reward:Validity:Block | 4 | 0.30 | .99 |
| GVS:Side:Block | 4 | 4.25 | .37 |
| Reward:Side:Block | 4 | 5.44 | .24 |
| Validity:Side:Block | 2 | 0.14 | .93 |
| GVS:Reward:Validity:Side | 4 | 7.67 | .10 |
| GVS:Reward:Validity:Block | 8 | 3.35 | .91 |
| GVS:Reward:Side:Block | 8 | 8.06 | .43 |
| GVS:Validity:Side:Block | 4 | 0.42 | .98 |
| Reward:Validity:Side:Block | 4 | 8.18 + | .09 |
| GVS:Reward:Validity:Side:Block | 8 | 7.06 | .53 |

Only the subset of significant results (uncorrected):

```
subset(rts_models$anova_table, `Pr(>Chisq)`<0.05)
```

|  | Df | Chisq | Chi Df | Pr(>Chisq) |
| --- | --- | --- | --- | --- |
| Reward | 29 | 29.245601 | 2 | 0.0000004 |
| Validity | 30 | 9.229727 | 1 | 0.0023812 |
| Side | 30 | 55.236615 | 1 | 0.0000000 |
| Block | 29 | 12.628150 | 2 | 0.0018106 |
| GVS:Reward | 52 | 19.105827 | 4 | 0.0007492 |
| Validity:Side | 55 | 5.030337 | 1 | 0.0249070 |
| GVS:Block | 52 | 23.629922 | 4 | 0.0000947 |
| Reward:Block | 52 | 12.714756 | 4 | 0.0127570 |
| Validity:Block | 54 | 9.797591 | 2 | 0.0074556 |
| GVS:Reward:Block | 86 | 18.433195 | 8 | 0.0182032 |

However, there results are uncorrected for multiple testing. We declared our interesting tests to be: Reward, Reward by Validity, GVS by Reward, and GVS by Reward by Validity. We apply a fdr correction to these values and to the others separately:

```
interesting_tests= c("Reward", "GVS:Reward", "Reward:Validity", "GVS:Reward:Validity")
rts_models$anova_table$p_fdr= NA

rts_models$anova_table[row.names(rts_models$anova_table) %in% interesting_tests, ]$p_fdr=
p.adjust(rts_models$anova_table[row.names(rts_models$anova_table) %in% interesting_tests, ]$`Pr(>Chisq)`, "fdr")

rts_models$anova_table[!row.names(rts_models$anova_table) %in% interesting_tests, ]$p_fdr=
p.adjust(rts_models$anova_table[!row.names(rts_models$anova_table) %in% interesting_tests, ]$`Pr(>Chisq)`, "fdr")

cbind(rts_models$anova_table, Sig= as.character(ifelse(rts_models$anova_table$p_fdr<0.043, "*", " ")))
```

|  | Df | Chisq | Chi Df | Pr(>Chisq) | p\_fdr | Sig |
| --- | --- | --- | --- | --- | --- | --- |
| GVS | 29 | 1.8971725 | 2 | 0.3872882 | 0.6311975 |  |
| Reward | 29 | 29.2456012 | 2 | 0.0000004 | 0.0000018 | \* |
| Validity | 30 | 9.2297267 | 1 | 0.0023812 | 0.0160729 | \* |
| Side | 30 | 55.2366149 | 1 | 0.0000000 | 0.0000000 | \* |
| Block | 29 | 12.6281498 | 2 | 0.0018106 | 0.0160729 | \* |
| GVS:Reward | 52 | 19.1058274 | 4 | 0.0007492 | 0.0014984 | \* |
| GVS:Validity | 54 | 2.1830270 | 2 | 0.3357080 | 0.6311975 |  |
| Reward:Validity | 54 | 3.6911560 | 2 | 0.1579340 | 0.2105787 |  |
| GVS:Side | 54 | 3.3726263 | 2 | 0.1852011 | 0.4167024 |  |
| Reward:Side | 54 | 1.0507034 | 2 | 0.5913473 | 0.7603037 |  |
| Validity:Side | 55 | 5.0303375 | 1 | 0.0249070 | 0.0840613 |  |
| GVS:Block | 52 | 23.6299217 | 4 | 0.0000947 | 0.0012790 | \* |
| Reward:Block | 52 | 12.7147557 | 4 | 0.0127570 | 0.0574066 |  |
| Validity:Block | 54 | 9.7975909 | 2 | 0.0074556 | 0.0402600 | \* |
| Side:Block | 54 | 0.2709016 | 2 | 0.8733221 | 0.9897857 |  |
| GVS:Reward:Validity | 90 | 3.9794304 | 4 | 0.4087968 | 0.4087968 |  |
| GVS:Reward:Side | 90 | 3.7272429 | 4 | 0.4441760 | 0.6311975 |  |
| GVS:Validity:Side | 92 | 1.7022537 | 2 | 0.4269336 | 0.6311975 |  |
| Reward:Validity:Side | 92 | 4.8196122 | 2 | 0.0898327 | 0.2425483 |  |
| GVS:Reward:Block | 86 | 18.4331948 | 8 | 0.0182032 | 0.0702123 |  |
| GVS:Validity:Block | 90 | 1.6315306 | 4 | 0.8031143 | 0.9856402 |  |
| Reward:Validity:Block | 90 | 0.3004402 | 4 | 0.9897857 | 0.9897857 |  |
| GVS:Side:Block | 90 | 4.2491294 | 4 | 0.3733385 | 0.6311975 |  |
| Reward:Side:Block | 90 | 5.4431998 | 4 | 0.2447676 | 0.5083634 |  |
| Validity:Side:Block | 92 | 0.1442789 | 2 | 0.9304012 | 0.9897857 |  |
| GVS:Reward:Validity:Side | 118 | 7.6711486 | 4 | 0.1043949 | 0.2562420 |  |
| GVS:Reward:Validity:Block | 114 | 3.3503088 | 8 | 0.9104917 | 0.9897857 |  |
| GVS:Reward:Side:Block | 114 | 8.0550822 | 8 | 0.4281081 | 0.6311975 |  |
| GVS:Validity:Side:Block | 118 | 0.4227700 | 4 | 0.9805706 | 0.9897857 |  |
| Reward:Validity:Side:Block | 118 | 8.1812351 | 4 | 0.0851604 | 0.2425483 |  |
| GVS:Reward:Validity:Side:Block | 122 | 7.0643053 | 8 | 0.5297108 | 0.7151096 |  |

This was the full table. We can thus display only tests that survive our corrections:

```
subset(rts_models$anova_table, `p_fdr`<0.043)
```

|  | Df | Chisq | Chi Df | Pr(>Chisq) | p\_fdr |
| --- | --- | --- | --- | --- | --- |
| Reward | 29 | 29.245601 | 2 | 0.0000004 | 0.0000018 |
| Validity | 30 | 9.229727 | 1 | 0.0023812 | 0.0160729 |
| Side | 30 | 55.236615 | 1 | 0.0000000 | 0.0000000 |
| Block | 29 | 12.628150 | 2 | 0.0018106 | 0.0160729 |
| GVS:Reward | 52 | 19.105827 | 4 | 0.0007492 | 0.0014984 |
| GVS:Block | 52 | 23.629922 | 4 | 0.0000947 | 0.0012790 |
| Validity:Block | 54 | 9.797591 | 2 | 0.0074556 | 0.0402600 |

These tests - because we planned sequential analyses at 24 and then 30 subjects - also survive correction for multiple longitudinal data assessment (equals to set a new alpha level of 0.043 instead of 0.05). We now run post-hoc tests to see which contrast reaches significance. For Reward:

```
lsm.options(lmer.df = "asymptotic") # the fastest, no df
lsmeans(rts_models, pairwise ~ Reward, adjust= "fdr")$contrast
```

```
## lsmeans are based on full model which includes all effects.
```

```
## NOTE: Results may be misleading due to involvement in interactions
```

```
##  contrast     estimate        SE df z.ratio p.value
##  None - Small 2.130682 0.7678204 NA   2.775  0.0055
##  None - High  7.668782 1.1235078 NA   6.826  <.0001
##  Small - High 5.538101 0.9024876 NA   6.136  <.0001
## 
## Results are averaged over the levels of: GVS, Validity, Side, Block 
## P value adjustment: fdr method for 3 tests
```

All contrasts reached significance, confirming the positive effects of Rewards (and motivation) on performance. The second test of interest was GVS by Reward:

```
lsmeans(rts_models, pairwise ~ GVS|Reward, adjust= "fdr")$contrast
```

```
## lsmeans are based on full model which includes all effects.
```

```
## NOTE: Results may be misleading due to involvement in interactions
```

```
## Reward = None:
##  contrast                     estimate       SE df z.ratio p.value
##  Left-Anodal - SHAM          1.3397829 2.140888 NA   0.626  0.5314
##  Left-Anodal - Right-Anodal  3.1242593 2.282657 NA   1.369  0.5133
##  SHAM - Right-Anodal         1.7844764 2.394345 NA   0.745  0.5314
## 
## Reward = Small:
##  contrast                     estimate       SE df z.ratio p.value
##  Left-Anodal - SHAM          1.6252414 2.139069 NA   0.760  0.6711
##  Left-Anodal - Right-Anodal  2.2940305 2.281169 NA   1.006  0.6711
##  SHAM - Right-Anodal         0.6687891 2.392446 NA   0.280  0.7798
## 
## Reward = High:
##  contrast                     estimate       SE df z.ratio p.value
##  Left-Anodal - SHAM          4.8575736 2.132979 NA   2.277  0.0683
##  Left-Anodal - Right-Anodal  0.1851978 2.274948 NA   0.081  0.9351
##  SHAM - Right-Anodal        -4.6723758 2.388588 NA  -1.956  0.0757
## 
## Results are averaged over the levels of: Validity, Side, Block 
## P value adjustment: fdr method for 3 tests
```

From the first we can see that the No Reward and Small reward conditions do not differ across GVS conditions; it is the same for the High Reward condition though we can definetly observe a trend (at the fdr threshold) for the two GVS conditions being less effective. Another way to turn the data is the following:

```
lsmeans(rts_models, pairwise ~ Reward|GVS, adjust= "fdr")$contrast
```

```
## lsmeans are based on full model which includes all effects.
```

```
## NOTE: Results may be misleading due to involvement in interactions
```

```
## GVS = Left-Anodal:
##  contrast      estimate       SE df z.ratio p.value
##  None - Small  2.312272 1.200393 NA   1.926  0.0541
##  None - High   7.475872 1.449162 NA   5.159  <.0001
##  Small - High  5.163601 1.284833 NA   4.019  0.0001
## 
## GVS = SHAM:
##  contrast      estimate       SE df z.ratio p.value
##  None - Small  2.597730 1.195204 NA   2.173  0.0297
##  None - High  10.993663 1.448289 NA   7.591  <.0001
##  Small - High  8.395933 1.282882 NA   6.545  <.0001
## 
## GVS = Right-Anodal:
##  contrast      estimate       SE df z.ratio p.value
##  None - Small  1.482043 1.199894 NA   1.235  0.2168
##  None - High   4.536811 1.451485 NA   3.126  0.0053
##  Small - High  3.054768 1.286803 NA   2.374  0.0264
## 
## Results are averaged over the levels of: Validity, Side, Block 
## P value adjustment: fdr method for 3 tests
```

The effect of Reward is more or less present everywhere, though of different magnitude. The most correct way for an interaction is probably to explore deltas:

```
lsmeans(rts_models, pairwise ~ Reward|GVS, interaction= T, adjust= "fdr")$contrast
```

```
## lsmeans are based on full model which includes all effects.
```

```
## NOTE: Results may be misleading due to involvement in interactions
```

```
##  Reward_pairwise GVS_pairwise                 estimate       SE df z.ratio
##  None - Small    Left-Anodal - SHAM         -0.2854585 1.592904 NA  -0.179
##  None - High     Left-Anodal - SHAM         -3.5177907 1.585035 NA  -2.219
##  Small - High    Left-Anodal - SHAM         -3.2323322 1.582391 NA  -2.043
##  None - Small    Left-Anodal - Right-Anodal  0.8302288 1.596342 NA   0.520
##  None - High     Left-Anodal - Right-Anodal  2.9390615 1.587886 NA   1.851
##  Small - High    Left-Anodal - Right-Anodal  2.1088327 1.585571 NA   1.330
##  None - Small    SHAM - Right-Anodal         1.1156873 1.592488 NA   0.701
##  None - High     SHAM - Right-Anodal         6.4568522 1.587171 NA   4.068
##  Small - High    SHAM - Right-Anodal         5.3411649 1.583977 NA   3.372
##  p.value
##   0.8578
##   0.0794
##   0.0924
##   0.6784
##   0.1155
##   0.2753
##   0.6217
##   0.0004
##   0.0034
## 
## Results are averaged over the levels of: Validity, Side, Block 
## P value adjustment: fdr method for 9 tests
```

The differences in the None vs High and Small vs. High are larger for the SHAM condition with respect to Right-Anodal (all p<0.004); similar trend for Left-Anodal, but does not survive fdr correction (all p ~0.08)

At the uncorrected threshold we found that this effect interacted with Block. It might be worth to have a look:

```
lsmeans(rts_models, pairwise ~ Reward:GVS|Block, interaction= T, adjust= "fdr")$contrast
```

```
## lsmeans are based on full model which includes all effects.
```

```
## NOTE: Results may be misleading due to involvement in interactions
```

```
##  Reward_pairwise GVS_pairwise               Block_pairwise    estimate
##  None - Small    Left-Anodal - SHAM         1 - 2          -1.28962462
##  None - High     Left-Anodal - SHAM         1 - 2           3.17537049
##  Small - High    Left-Anodal - SHAM         1 - 2           4.46499511
##  None - Small    Left-Anodal - Right-Anodal 1 - 2          -4.51407076
##  None - High     Left-Anodal - Right-Anodal 1 - 2          -0.11866881
##  Small - High    Left-Anodal - Right-Anodal 1 - 2           4.39540195
##  None - Small    SHAM - Right-Anodal        1 - 2          -3.22444614
##  None - High     SHAM - Right-Anodal        1 - 2          -3.29403930
##  Small - High    SHAM - Right-Anodal        1 - 2          -0.06959316
##  None - Small    Left-Anodal - SHAM         1 - 3           3.69838670
##  None - High     Left-Anodal - SHAM         1 - 3           8.98241818
##  Small - High    Left-Anodal - SHAM         1 - 3           5.28403148
##  None - Small    Left-Anodal - Right-Anodal 1 - 3          -3.75110707
##  None - High     Left-Anodal - Right-Anodal 1 - 3           9.42091395
##  Small - High    Left-Anodal - Right-Anodal 1 - 3          13.17202102
##  None - Small    SHAM - Right-Anodal        1 - 3          -7.44949377
##  None - High     SHAM - Right-Anodal        1 - 3           0.43849577
##  Small - High    SHAM - Right-Anodal        1 - 3           7.88798954
##  None - Small    Left-Anodal - SHAM         2 - 3           4.98801132
##  None - High     Left-Anodal - SHAM         2 - 3           5.80704768
##  Small - High    Left-Anodal - SHAM         2 - 3           0.81903637
##  None - Small    Left-Anodal - Right-Anodal 2 - 3           0.76296369
##  None - High     Left-Anodal - Right-Anodal 2 - 3           9.53958275
##  Small - High    Left-Anodal - Right-Anodal 2 - 3           8.77661907
##  None - Small    SHAM - Right-Anodal        2 - 3          -4.22504763
##  None - High     SHAM - Right-Anodal        2 - 3           3.73253507
##  Small - High    SHAM - Right-Anodal        2 - 3           7.95758270
##        SE df z.ratio p.value
##  3.908236 NA  -0.330  0.9099
##  3.893299 NA   0.816  0.5332
##  3.889838 NA   1.148  0.5002
##  3.917870 NA  -1.152  0.5002
##  3.901438 NA  -0.030  0.9857
##  3.897002 NA   1.128  0.5002
##  3.902389 NA  -0.826  0.5332
##  3.893244 NA  -0.846  0.5332
##  3.888135 NA  -0.018  0.9857
##  3.902162 NA   0.948  0.5149
##  3.880223 NA   2.315  0.1254
##  3.875166 NA   1.364  0.4663
##  3.911858 NA  -0.959  0.5149
##  3.885961 NA   2.424  0.1254
##  3.884931 NA   3.391  0.0188
##  3.903423 NA  -1.908  0.1901
##  3.884916 NA   0.113  0.9829
##  3.881116 NA   2.032  0.1624
##  3.894316 NA   1.281  0.4915
##  3.871055 NA   1.500  0.4007
##  3.861047 NA   0.212  0.9506
##  3.900896 NA   0.196  0.9506
##  3.877760 NA   2.460  0.1254
##  3.866517 NA   2.270  0.1254
##  3.895221 NA  -1.085  0.5005
##  3.880854 NA   0.962  0.5149
##  3.867358 NA   2.058  0.1624
## 
## Results are averaged over the levels of: Validity, Side 
## P value adjustment: fdr method for 27 tests
```

Not much actually reaches the threshold…

Before moving on to graphical depictions, we can check the other tests (not the focus of this work, but possibly important).

Validity for example, we did expect a strong IOR:

```
lsmeans(rts_models, pairwise ~ Validity, adjust= "fdr")$contrast
```

```
## lsmeans are based on full model which includes all effects.
```

```
## NOTE: Results may be misleading due to involvement in interactions
```

```
##  contrast         estimate       SE df z.ratio p.value
##  Invalid - Valid -4.683582 1.442746 NA  -3.246  0.0012
## 
## Results are averaged over the levels of: GVS, Reward, Side, Block
```

It was found to be modulated by Block anyway:

```
lsmeans(rts_models, pairwise ~ Validity|Block, interaction= T, adjust= "fdr")$contrast
```

```
## lsmeans are based on full model which includes all effects.
```

```
## NOTE: Results may be misleading due to involvement in interactions
```

```
##  Validity_pairwise Block_pairwise estimate       SE df z.ratio p.value
##  Invalid - Valid   1 - 2          2.750024 1.299658 NA   2.116  0.0515
##  Invalid - Valid   1 - 3          3.942528 1.296719 NA   3.040  0.0071
##  Invalid - Valid   2 - 3          1.192504 1.293190 NA   0.922  0.3565
## 
## Results are averaged over the levels of: GVS, Reward, Side 
## P value adjustment: fdr method for 3 tests
```

IOR was confirmed and it grews gradually in time, being larger for blocks 2 and 3 with respect to block 1.

Side: the right side was about 4 ms faster.

```
lsmeans(rts_models, pairwise ~ Side, adjust= "fdr")$contrast
```

```
## lsmeans are based on full model which includes all effects.
```

```
## NOTE: Results may be misleading due to involvement in interactions
```

```
##  contrast     estimate        SE df z.ratio p.value
##  Left - Right 3.926755 0.5293108 NA   7.419  <.0001
## 
## Results are averaged over the levels of: GVS, Reward, Validity, Block
```

The effect of Block:

```
lsmeans(rts_models, pairwise ~ Block, adjust= "fdr")$contrast
```

```
## lsmeans are based on full model which includes all effects.
```

```
## NOTE: Results may be misleading due to involvement in interactions
```

```
##  contrast   estimate        SE df z.ratio p.value
##  1 - 2    -2.2962205 0.6498699 NA  -3.533  0.0012
##  1 - 3    -0.9129523 0.6484285 NA  -1.408  0.1591
##  2 - 3     1.3832682 0.6465913 NA   2.139  0.0486
## 
## Results are averaged over the levels of: GVS, Reward, Validity, Side 
## P value adjustment: fdr method for 3 tests
```

Is actually modulated by GVS:

```
lsmeans(rts_models, pairwise ~ Block|GVS, interaction= T, adjust= "fdr")$contrast
```

```
## lsmeans are based on full model which includes all effects.
```

```
## NOTE: Results may be misleading due to involvement in interactions
```

```
##  Block_pairwise GVS_pairwise                 estimate       SE df z.ratio
##  1 - 2          Left-Anodal - SHAM          2.6158675 1.591102 NA   1.644
##  1 - 3          Left-Anodal - SHAM          7.2080700 1.586634 NA   4.543
##  2 - 3          Left-Anodal - SHAM          4.5922025 1.582212 NA   2.902
##  1 - 2          Left-Anodal - Right-Anodal -0.9474363 1.594455 NA  -0.594
##  1 - 3          Left-Anodal - Right-Anodal  2.5270324 1.590086 NA   1.589
##  2 - 3          Left-Anodal - Right-Anodal  3.4744686 1.584780 NA   2.192
##  1 - 2          SHAM - Right-Anodal        -3.5633038 1.590024 NA  -2.241
##  1 - 3          SHAM - Right-Anodal        -4.6810376 1.588203 NA  -2.947
##  2 - 3          SHAM - Right-Anodal        -1.1177339 1.584500 NA  -0.705
##  p.value
##   0.1440
##   <.0001
##   0.0111
##   0.5524
##   0.1440
##   0.0510
##   0.0510
##   0.0111
##   0.5406
## 
## Results are averaged over the levels of: Reward, Validity, Side 
## P value adjustment: fdr method for 9 tests
```

Subjects were slower with time in the SHAM, faster in Left-Anodal… Very weird…

### Plots

Here’s a function to plot data, with option to display variability and data distribution:

```
my_plot= function(DF, dv= c("correct", "response_time"), withinvars, betweenvars= NULL, 
                  idvar= "Subject", 
                  individual_data= c("none", "p", "l", "violin", "bp"),
                  plot.gain= T){
  
  #match arguments
  individual_data= match.arg(individual_data)
  dv= match.arg(dv)
  
  #concatenate all relevant variables
  allvars= c(withinvars, betweenvars)
  
  #summarise data according to grouping variables
  Xs= ddply(DF, c(allvars, idvar), summarise, y= mean(get(dv)))
  
  #set axis label
  if(dv=="correct")(ylab= "Accuracy") else (ylab= "Reaction times (ms)")
  
  #if Validity is involved, summarise the deltas
  #code everything as "gain", so it changes for rts and accuracy (positive diff= gain)
  if("Validity" %in% allvars & plot.gain== T){
    
    print("Validity is involved: plotting deltas")
    
    if(dv== "correct"){
    X.temp= subset(Xs, Validity== "Valid") 
    X.temp$y= X.temp$y - subset(Xs, Validity== "Invalid")$y} else {
      X.temp= subset(Xs, Validity== "Invalid") 
      X.temp$y= X.temp$y - subset(Xs, Validity== "Valid")$y}

    Xs= X.temp[, !names(X.temp) %in% "Validity"]
    
    withinvars= withinvars[!withinvars %in% "Validity"]
    allvars= allvars[!allvars %in% "Validity"]
     
    if(dv=="correct")(ylab= "Accuracy (gain)") else (ylab= "Reaction times (gain, ms)")
    }
  
  Xs[, idvar]= as.factor(Xs[, idvar])
  
  #obtain summary statistics
  X= summarySEwithin(Xs, measurevar = "y", 
                     betweenvars = betweenvars, 
                     withinvars = withinvars, idvar = idvar)

  #finally depict!
  p= ggplot(X, aes(y= y, x= get(allvars[1]), fill= get(allvars[1]))) + commonTheme 
  
  #if it's a delta plot line @ 0
  if(grepl("gain", ylab))(p= p + geom_hline(yintercept = 0, linetype= "dashed", size= 1.1,
                                            color= ifelse(individual_data=="l", "darkgray", "gray")))
  
  #if data distribution is asked for
  if(individual_data=="l"){
    p= p + geom_line(data = Xs, aes(x= get(allvars[1]), y=  y, group= get(idvar)), 
                     size= 1.2, color= "dark gray", alpha= 0.5)} 
  
  if(individual_data=="p"){
    pd= position_dodge(0.5)
    p= p + geom_point(data = Xs, aes(x= get(allvars[1]), y=  y, group= get(idvar)),
                      position = pd, size= 3, shape= 21, color= "black", alpha= 0.3)} 
  
  if(individual_data=="violin"){
    p= p + geom_violin(data = Xs, aes(x= get(allvars[1]), y=  y), alpha= 0.3)} 
  
  if(individual_data=="bp"){
    p= p + geom_boxplot(data = Xs, aes(x= get(allvars[1]), y=  y), alpha= 0.3, 
                        outlier.size = 3.5)} 
  
  #plot summary statistics
  if(individual_data=="none"){
    p= p + geom_errorbar(data=X, aes(ymin= y-se, ymax= y+se), 
                       size= 1.5, width= .2, colour= "black") + 
    geom_point(size= 6, stroke= 2, shape= 21, color= "black")
  }
  
   
  p= p + labs(y= ylab, x= allvars[1]) + guides(fill=F)
  
  #for many variables at once, wrapping
  if(length(allvars)>1) (p= p + facet_wrap(allvars[2:length(allvars)]))
  
  #final plot
  return(p)  
  
}
```

We can thus explore…

The main effect of Reward:

```
my_plot(ART, dv <- "response_time", withinvars = c("Reward"), 
       individual_data = "none")
```

The two-way Reward by Validity (key test, but not significant):

```
my_plot(ART, dv <- "response_time", withinvars = c("Validity", "Reward"), 
       individual_data = "none")
```

```
## [1] "Validity is involved: plotting deltas"
```

The two-way Reward by GVS:

```
my_plot(ART, dv <- "response_time", withinvars = c("Reward", "GVS"), 
       individual_data = "none")
```

A closer look to variability:

```
p1= my_plot(ART, dv <- "response_time", withinvars = c("Reward", "GVS"), 
       individual_data = "p")
p2= my_plot(ART, dv <- "response_time", withinvars = c("Reward", "GVS"), 
       individual_data = "l")
p3= my_plot(ART, dv <- "response_time", withinvars = c("Reward", "GVS"), 
       individual_data = "violin")
p4= my_plot(ART, dv <- "response_time", withinvars = c("Reward", "GVS"), 
       individual_data = "bp")
 
grid.arrange(p1, p2, p3, p4)
```

The three-way Reward by Validity by GVS (not significant, but of interest):

```
my_plot(ART, dv <- "response_time", withinvars = c("Reward", "GVS", "Validity"), 
       individual_data = "none")
```

```
## [1] "Validity is involved: plotting deltas"
```

Other findings classified as being of secondary interest.

Validity and Validity by Block:

```
my_plot(ART, dv <- "response_time", withinvars = c("Validity"), 
       individual_data = "none", plot.gain=F)
```

```
## Automatically converting the following non-factors to factors: Validity
```

```
my_plot(ART, dv <- "response_time", withinvars = c("Validity", "Block"), 
       individual_data = "none", plot.gain=T)
```

```
## [1] "Validity is involved: plotting deltas"
```

Side:

```
my_plot(ART, dv <- "response_time", withinvars = c("Side"), 
       individual_data = "none")
```

```
## Automatically converting the following non-factors to factors: Side
```

Block and GVS by Block:

```
my_plot(ART, dv <- "response_time", withinvars = c("Block"), 
       individual_data = "none")
```

```
my_plot(ART, dv <- "response_time", withinvars = c("Block", "GVS"), 
       individual_data = "none")
```

We are done for RTs! We still have to assess accuracy, but we will do it on a companion file to exploit a clean R session (and thus avoid the infamous “cannot allocate vector of size…” R problem).

## Appendix

This is the function to summarise data (`summarySEwithin`):

```
## Summarizes data.
## Gives count, mean, standard deviation, standard error of the mean, and confidence interval (default 95%).
##   data: a data frame.
##   measurevar: the name of a column that contains the variable to be summariezed
##   groupvars: a vector containing names of columns that contain grouping variables
##   na.rm: a boolean that indicates whether to ignore NA's
##   conf.interval: the percent range of the confidence interval (default is 95%)
summarySE <- function(data=NULL, measurevar, groupvars=NULL, na.rm=FALSE,
                      conf.interval=.95, .drop=TRUE) {
  library(plyr)
  
  # New version of length which can handle NA's: if na.rm==T, don't count them
  length2 <- function (x, na.rm=FALSE) {
    if (na.rm) sum(!is.na(x))
    else       length(x)
  }
  
  # This does the summary. For each group's data frame, return a vector with
  # N, mean, and sd
  datac <- ddply(data, groupvars, .drop=.drop,
                 .fun = function(xx, col) {
                   c(N    = length2(xx[[col]], na.rm=na.rm),
                     mean = mean   (xx[[col]], na.rm=na.rm),
                     sd   = sd     (xx[[col]], na.rm=na.rm)
                   )
                 },
                 measurevar
  )
  
  # Rename the "mean" column    
  datac <- plyr::rename(datac, c(mean = measurevar))
  
  datac$se <- datac$sd / sqrt(datac$N)  # Calculate standard error of the mean
  
  # Confidence interval multiplier for standard error
  # Calculate t-statistic for confidence interval: 
  # e.g., if conf.interval is .95, use .975 (above/below), and use df=N-1
  ciMult <- qt(conf.interval/2 + .5, datac$N-1)
  datac$ci <- datac$se * ciMult
  
  return(datac)
}


## Norms the data within specified groups in a data frame; it normalizes each
## subject (identified by idvar) so that they have the same mean, within each group
## specified by betweenvars.
##   data: a data frame.
##   idvar: the name of a column that identifies each subject (or matched subjects)
##   measurevar: the name of a column that contains the variable to be summariezed
##   betweenvars: a vector containing names of columns that are between-subjects variables
##   na.rm: a boolean that indicates whether to ignore NA's
normDataWithin <- function(data=NULL, idvar, measurevar, betweenvars=NULL,
                           na.rm=FALSE, .drop=TRUE) {
  library(plyr)
  
  # Measure var on left, idvar + between vars on right of formula.
  data.subjMean <- ddply(data, c(idvar, betweenvars), .drop=.drop,
                         .fun = function(xx, col, na.rm) {
                           c(subjMean = mean(xx[,col], na.rm=na.rm))
                         },
                         measurevar,
                         na.rm
  )
  
  # Put the subject means with original data
  data <- merge(data, data.subjMean)
  
  # Get the normalized data in a new column
  measureNormedVar <- paste(measurevar, "_norm", sep="")
  data[,measureNormedVar] <- data[,measurevar] - data[,"subjMean"] +
    mean(data[,measurevar], na.rm=na.rm)
  
  # Remove this subject mean column
  data$subjMean <- NULL
  
  return(data)
}


## Retrieved here: http://www.cookbook-r.com/Graphs/Plotting_means_and_error_bars_(ggplot2)/
## Summarizes data, handling within-subjects variables by removing inter-subject variability.
## It will still work if there are no within-S variables.
## Gives count, un-normed mean, normed mean (with same between-group mean),
##   standard deviation, standard error of the mean, and confidence interval.
## If there are within-subject variables, calculate adjusted values using method from Morey (2008).
##   data: a data frame.
##   measurevar: the name of a column that contains the variable to be summariezed
##   betweenvars: a vector containing names of columns that are between-subjects variables
##   withinvars: a vector containing names of columns that are within-subjects variables
##   idvar: the name of a column that identifies each subject (or matched subjects)
##   na.rm: a boolean that indicates whether to ignore NA's
##   conf.interval: the percent range of the confidence interval (default is 95%)
summarySEwithin <- function(data=NULL, measurevar, betweenvars=NULL, withinvars=NULL,
                            idvar=NULL, na.rm=FALSE, conf.interval=.95, .drop=TRUE) {
  
  # Ensure that the betweenvars and withinvars are factors
  factorvars <- vapply(data[, c(betweenvars, withinvars), drop=FALSE],
                       FUN=is.factor, FUN.VALUE=logical(1))
  
  if (!all(factorvars)) {
    nonfactorvars <- names(factorvars)[!factorvars]
    message("Automatically converting the following non-factors to factors: ",
            paste(nonfactorvars, collapse = ", "))
    data[nonfactorvars] <- lapply(data[nonfactorvars], factor)
  }
  
  # Get the means from the un-normed data
  datac <- summarySE(data, measurevar, groupvars=c(betweenvars, withinvars),
                     na.rm=na.rm, conf.interval=conf.interval, .drop=.drop)
  
  # Drop all the unused columns (these will be calculated with normed data)
  datac$sd <- NULL
  datac$se <- NULL
  datac$ci <- NULL
  
  # Norm each subject's data
  ndata <- normDataWithin(data, idvar, measurevar, betweenvars, na.rm, .drop=.drop)
  
  # This is the name of the new column
  measurevar_n <- paste(measurevar, "_norm", sep="")
  
  # Collapse the normed data - now we can treat between and within vars the same
  ndatac <- summarySE(ndata, measurevar_n, groupvars=c(betweenvars, withinvars),
                      na.rm=na.rm, conf.interval=conf.interval, .drop=.drop)
  
  # Apply correction from Morey (2008) to the standard error and confidence interval
  #  Get the product of the number of conditions of within-S variables
  nWithinGroups    <- prod(vapply(ndatac[,withinvars, drop=FALSE], FUN=nlevels,
                                  FUN.VALUE=numeric(1)))
  correctionFactor <- sqrt( nWithinGroups / (nWithinGroups-1) )
  
  # Apply the correction factor
  ndatac$sd <- ndatac$sd * correctionFactor
  ndatac$se <- ndatac$se * correctionFactor
  ndatac$ci <- ndatac$ci * correctionFactor
  
  # Combine the un-normed means with the normed results
  merge(datac, ndatac)
}
```
