# Supplementary material for: Probing the role of the vestibular system in motivation and reward-based attention
Source: Cortex. 2018 Jun;103:82–99. doi: 10.1016/j.cortex.2018.02.009 (PMC6002611; doi:10.1016/j.cortex.2018.02.009)
Supplement: Supplementary file 4 [file mmc4.zip › cortex_2252_mmc4.html]

S4: ART task, accuracy


# S4: ART task, accuracy

#### *Elvio Blini INSERM U1028, ImpAct team, CRNL, and University of Lyon elvio.blini@gmail.com*

#### *30 November 2017*

# Attention and Reward Task (ART)

This is the main task administered to subjects. Each trial started with a fixation cross lasting 750ms. Then, one of the two lateral placeholders, either the left or right one, changed colour for 100 ms (cue). After additional 600 ms (on average) a target - either square or circle - was presented either on the left or right side of the screen. The position of the cue was not predictive of the side of target presentation (50%). However, the color of the cue predicted (100%) the amount of points that the subject could earn for a correct and fast response. A feeback was presented at the end of each trial reporting whether the response was correct, incorrect, too fast, or too slow; the number of points rewarded for the current trial; the overall gain since the beginning of the task.

Time course of a typical trial of the ART.

There’s a whealth on information we can extract from this task. We expected, to begin with, a main effect of Reward, suggesting that our manipulation effectively modulated motivation. Then, we sought to look for the interaction of Reward and Validity. In theory, motivational and spatial cues might interact, and the validity gain/inhibition of return commonly observed in Posner-like tasks might be modulated consequently. With this respect, studies in literature have been inconsistent, though the specific paradigm employed changed sensibly as well. In a study very similar to ours, high rewards were found to enhance inhibition of return specifically (Bucker and Theeuwes, 2014). However, the key interests of our study were the possible interactions with a vestibular stimulation (GVS, see the companion paper). The first question was whether GVS modulates sensitivity to rewards. With this respect, we wondered whether reward-based performance boosts could be further modulated by GVS, either in the sense of an abolishment, or in the sense of a further enhancement (bi-directional hypotheses). Finally, a three-way interaction GVS by Reward by Validity was also predicted, showing that GVS might affect the interplay between motivation and spatial attention (again, please refer to the main text for the rationale).

**This document presents results for accuracy, a companion file, S3, reports the results for reaction times.**

## Preliminary setup

It’s sometimes good to clean the current environment to avoid conflicts. You can do it with `rm(list=ls())` (but be sure everything is properly saved for future use).

In order to run this script we need a few packages available on CRAN. You might need to install them first, e.g. by typing `install.packages("BayesFactor")` in the console.

```
#list packages
packages= c("plyr", "magrittr", "tidyverse", "BayesFactor", "lme4", "multcomp",
            "gridExtra", "ez" , "lsmeans", "retimes")

#load them
lapply(packages, require, character.only= T)
```

For package `afex` I’m using the developer version on github (you will need to install `devtools`).

```
#devtools::install_github("singmann/afex@master")
library(afex)
```

For reproducibility, here’s details of the system on which the script was tested and a seed for random numbers:

```
set.seed(1)

sessionInfo()
```

```
## R version 3.4.2 (2017-09-28)
## Platform: x86_64-w64-mingw32/x64 (64-bit)
## Running under: Windows 10 x64 (build 15063)
## 
## Matrix products: default
## 
## locale:
## [1] LC_COLLATE=English_United Kingdom.1252 
## [2] LC_CTYPE=English_United Kingdom.1252   
## [3] LC_MONETARY=English_United Kingdom.1252
## [4] LC_NUMERIC=C                           
## [5] LC_TIME=English_United Kingdom.1252    
## 
## attached base packages:
## [1] stats     graphics  grDevices utils     datasets  methods   base     
## 
## other attached packages:
##  [1] afex_0.18-0          retimes_0.1-2        lsmeans_2.27-2      
##  [4] estimability_1.2     ez_4.4-0             gridExtra_2.3       
##  [7] multcomp_1.4-7       TH.data_1.0-8        MASS_7.3-47         
## [10] survival_2.41-3      mvtnorm_1.0-6        lme4_1.1-14         
## [13] BayesFactor_0.9.12-2 Matrix_1.2-11        coda_0.19-1         
## [16] dplyr_0.7.4          purrr_0.2.3          readr_1.1.1         
## [19] tidyr_0.7.1          tibble_1.3.4         ggplot2_2.2.1       
## [22] tidyverse_1.1.1      magrittr_1.5         plyr_1.8.4          
## [25] printr_0.1          
## 
## loaded via a namespace (and not attached):
##  [1] nlme_3.1-131        pbkrtest_0.4-7      lubridate_1.6.0    
##  [4] RColorBrewer_1.1-2  httr_1.3.1          rprojroot_1.2      
##  [7] tools_3.4.2         backports_1.1.1     R6_2.2.2           
## [10] rpart_4.1-11        Hmisc_4.0-3         lazyeval_0.2.0     
## [13] mgcv_1.8-20         colorspace_1.3-2    nnet_7.3-12        
## [16] mnormt_1.5-5        compiler_3.4.2      rvest_0.3.2        
## [19] quantreg_5.33       htmlTable_1.9       SparseM_1.77       
## [22] xml2_1.1.1          sandwich_2.4-0      checkmate_1.8.4    
## [25] scales_0.5.0        psych_1.7.8         pbapply_1.3-3      
## [28] stringr_1.2.0       digest_0.6.12       foreign_0.8-69     
## [31] minqa_1.2.4         rmarkdown_1.7       base64enc_0.1-3    
## [34] pkgconfig_2.0.1     htmltools_0.3.6     htmlwidgets_0.9    
## [37] rlang_0.1.2         readxl_1.0.0        bindr_0.1          
## [40] zoo_1.8-0           jsonlite_1.5        gtools_3.5.0       
## [43] acepack_1.4.1       car_2.1-5           modeltools_0.2-21  
## [46] Formula_1.2-2       Rcpp_0.12.13        munsell_0.4.3      
## [49] stringi_1.1.5       yaml_2.1.14         grid_3.4.2         
## [52] parallel_3.4.2      forcats_0.2.0       lattice_0.20-35    
## [55] haven_1.1.0         splines_3.4.2       hms_0.3            
## [58] knitr_1.17          reshape2_1.4.2      codetools_0.2-15   
## [61] stats4_3.4.2        glue_1.1.1          evaluate_0.10.1    
## [64] latticeExtra_0.6-28 data.table_1.10.4   modelr_0.1.1       
## [67] nloptr_1.0.4        MatrixModels_0.4-1  cellranger_1.1.0   
## [70] gtable_0.2.0        assertthat_0.2.0    coin_1.2-1         
## [73] xtable_1.8-2        broom_0.4.2         lmerTest_2.0-33    
## [76] bindrcpp_0.2        cluster_2.0.6
```

Thanks to the function retrieved here, not displayed, the following hyperlinks download the Rdata files:

That can be loaded then with:

```
load("ART data.RData")
```

Now all relevant variables are stored in the `ART` data.frame, that you can navigate and explore with the usual commands, e.g. `str(ART)`.

## Useful functions

This is a minimal (shared by all plots) list of graphic attributes for ggplot:

```
#ggplot defaults
commonTheme = list(theme_bw(),
                   theme(text = element_text(size=18, face="bold"),
                         axis.text = element_text(size=16, face="bold", color= "black"),
                         plot.margin = unit(c(1,1,1,1), "cm")))
```

Then (ONLY SHOWN IN THE BOTTOM PART OF THIS SCRIPT) we have a convenient summarising function that produces means and within subjects SEM as in Morey (2008). I retrieved it here: link

## Preprocessing

A bunch of variables is to declare (e.g., numbers into factors). Then, for the sake of clarity, levels are renamed when helpful.

```
#declare
ART$Subject= as.factor(ART$Subject)

if(sum(levels(as.factor(ART$Reward))== c("0", "2", "10"))==3){
  ART$Reward= factor(ART$Reward, labels = c("None", "Small", "High"))} else 
    (warning("levels unordered? Check"))

if(sum(levels(as.factor(ART$GVS))== c("A", "B", "C"))==3){
  ART$GVS= factor(ART$GVS, labels = c("SHAM", "Left-Anodal", "Right-Anodal"))
  ART$GVS= relevel(ART$GVS, "Left-Anodal")} else (warning("levels unordered? Check"))
```

We must drop a few subjects ( :( ). Reasons are in the comments.

```
#subject 1 to replace for a bug in the script, my fault, :(
ART= ART[!ART$Subject== 1, ] #replaced with subject 51

#subject 10 didn't show up after first session, >:(
ART= ART[!ART$Subject== 10, ] #replaced with subject 60

ART$Subject= factor(ART$Subject)
```

We create a “Block” variable.

```
#insert variable "block"
ART$Block= ifelse(ART$Trial< 145, 1, 
                  ifelse(ART$Trial> 288, 3, 2)) %>% as.factor
```

A good response (valid) was defined as being correct, slower than 100ms, and faster than 500ms.

```
#exclusion criteria applies to valid responses
ART$Valid_Response= ifelse(ART$correct== 1 &
                           ART$response_time> 100 &
                           ART$response_time< 500, 1, 0)
```

We can start with our quality checks! First, subjects with less that 60% of trials per cell remaining have to be discarded:

```
#must be >60% for each subject
ddply(ART, "Subject", summarise, 
      acc= mean(Valid_Response)) %$% acc %>%
      sapply(function(x) x<0.6)
```

```
##  [1] FALSE FALSE FALSE FALSE FALSE FALSE FALSE FALSE FALSE FALSE FALSE
## [12] FALSE FALSE FALSE FALSE FALSE FALSE FALSE FALSE FALSE FALSE FALSE
## [23] FALSE FALSE FALSE FALSE FALSE FALSE FALSE FALSE
```

Second, the threshold was set to 30% when considering the GVS by Reward by Validity cells:

```
#must be >30% for each cell in the triple interaction
ddply(ART, c("GVS", "Reward", "Validity", "Subject"), summarise, 
      acc= mean(Valid_Response)) %>% 
      with(tapply(acc, Subject, function(x)(x<0.3))) %>%
      lapply(function(x) (sum(x)))>0
```

```
##     2     3     4     5     6     7     8     9    11    12    13    14 
## FALSE FALSE FALSE FALSE FALSE FALSE FALSE FALSE FALSE FALSE FALSE FALSE 
##    15    16    17    18    19    20    21    22    23    24    25    26 
## FALSE FALSE FALSE FALSE FALSE FALSE FALSE FALSE FALSE FALSE FALSE FALSE 
##    27    28    29    30    51    60 
## FALSE FALSE FALSE FALSE FALSE FALSE
```

Thus, so far no replacement is necessary! We had two criteria to proceed with analyses for accuracy (the objective was to avoid ceiling effects). The group mean must be less than 95%:

```
#group mean >95%
ddply(ART, "Subject", summarise, 
      acc= mean(correct)) %$% print(mean(acc))<0.95
```

```
## [1] 0.9420782
```

```
## [1] TRUE
```

Very close… Then, less than half of the subjects must present >95% of correct responses:

```
#less than half of subjects has >95%
ddply(ART, "Subject", summarise, 
      acc= mean(correct)) %$% print(mean(acc<0.95))>0.5
```

```
## [1] 0.5666667
```

```
## [1] TRUE
```

As it is now, we can proceed with analyses! (Though a few subjects still present 100% accuracy in some cells, I wonder whether this will be problematic when fitting mixed models…).

We can save a separate dataframe with wrong responses, this way we will work with reaction times for good responses only:

```
#save data for follow-up analyses and plots (before excluding wrong responses)
ART.accuracy= ART

#exclude bad responses
ART= ART[ART$Valid_Response==1, ]
```

Finally, another criterion for subjects’ replacement was the absence of extremely deviant reaction times in a subset of cells:

```
#our check was for cells in the GVS by Reward by Validity interaction for each subject
#a subject is replaced if exceeds +-3 standard deviations from his/her mean
ddply(ART, c("GVS", "Reward", "Validity", "Subject"), summarise, 
      rts= mean(response_time)) %>%
      with(tapply(rts, Subject, scale)) %>% 
      lapply(function(x) (ifelse(abs(as.numeric(x))>3, 1, 0))) %>%
      lapply(function(x) (sum(x)))>0
```

```
##     2     3     4     5     6     7     8     9    11    12    13    14 
## FALSE FALSE FALSE FALSE FALSE FALSE FALSE FALSE FALSE FALSE FALSE FALSE 
##    15    16    17    18    19    20    21    22    23    24    25    26 
## FALSE FALSE FALSE FALSE FALSE FALSE FALSE FALSE FALSE FALSE FALSE FALSE 
##    27    28    29    30    51    60 
## FALSE FALSE FALSE FALSE FALSE FALSE
```

All good!

## Analyses

### Accuracy

We now switch to testing accuracy. Our criteria were fulfilled, yet a few subjects reached the ceiling in a few cells and overall accuracy was very high. We repeat nevertheless the procedures adopted for RTs.

The starting model is:

```
accmod0= glmer(correct ~ (1|Subject), 
               data= ART.accuracy, family= binomial,
               control=glmerControl(optimizer="bobyqa"))
```

We then assess random slopes:

```
accmod0a= glmer(correct ~ (1+GVS|Subject), 
               data= ART.accuracy, family= binomial,
               control=glmerControl(optimizer="bobyqa"))

anova(accmod0, accmod0a)
```

|  | Df | AIC | BIC | logLik | deviance | Chisq | Chi Df | Pr(>Chisq) |
| --- | --- | --- | --- | --- | --- | --- | --- | --- |
| accmod0 | 2 | 16630.19 | 16647.33 | -8313.094 | 16626.19 | NA | NA | NA |
| accmod0a | 7 | 16521.51 | 16581.49 | -8253.755 | 16507.51 | 118.678 | 5 | 0 |

```
accmod0b= glmer(correct ~ (1+GVS+Reward|Subject), 
               data= ART.accuracy, family= binomial,
               control=glmerControl(optimizer="bobyqa", optCtrl=list(maxfun=10^20)))

# Warning messages:
# 1: In (function (par, fn, lower = -Inf, upper = Inf, control = list(),  :
#   NAs introduced by coercion to integer range
# 2: In optwrap(optimizer, devfun, start, rho$lower, control = control,  :
#   convergence code 1 from bobyqa: bobyqa -- maximum number of function evaluations exceeded
# 3: In (function (par, fn, lower = -Inf, upper = Inf, control = list(),  :
#   NAs introduced by coercion to integer range
# 4: In optwrap(optimizer, devfun, start, rho$lower, control = control,  :
#   convergence code 1 from bobyqa: bobyqa -- maximum number of function evaluations exceeded
# 5: In checkConv(attr(opt, "derivs"), opt$par, ctrl = control$checkConv,  :
#   unable to evaluate scaled gradient
# 6: In checkConv(attr(opt, "derivs"), opt$par, ctrl = control$checkConv,  :
#   Model failed to converge: degenerate  Hessian with 3 negative eigenvalues

accmod0c= glmer(correct ~ (1+GVS+Validity|Subject), 
               data= ART.accuracy, family= binomial,
               control=glmerControl(optimizer="bobyqa", optCtrl=list(maxfun=10^20)))

# Warning messages:
# 1: In (function (par, fn, lower = -Inf, upper = Inf, control = list(),  :
#   NAs introduced by coercion to integer range
# 2: In optwrap(optimizer, devfun, start, rho$lower, control = control,  :
#   convergence code 1 from bobyqa: bobyqa -- maximum number of function evaluations exceeded
# 3: In (function (par, fn, lower = -Inf, upper = Inf, control = list(),  :
#   NAs introduced by coercion to integer range
# 4: In optwrap(optimizer, devfun, start, rho$lower, control = control,  :
#   convergence code 1 from bobyqa: bobyqa -- maximum number of function evaluations exceeded
# 5: In checkConv(attr(opt, "derivs"), opt$par, ctrl = control$checkConv,  :
#   unable to evaluate scaled gradient
# 6: In checkConv(attr(opt, "derivs"), opt$par, ctrl = control$checkConv,  :
#   Model failed to converge: degenerate  Hessian with 2 negative eigenvalues

accmod0d= glmer(correct ~ (1+GVS+Side|Subject), 
               data= ART.accuracy, family= binomial,
               control=glmerControl(optimizer="bobyqa", optCtrl=list(maxfun=10^20)))

# Warning messages:
# 1: In (function (par, fn, lower = -Inf, upper = Inf, control = list(),  :
#   NAs introduced by coercion to integer range
# 2: In optwrap(optimizer, devfun, start, rho$lower, control = control,  :
#   convergence code 1 from bobyqa: bobyqa -- maximum number of function evaluations exceeded
# 3: In (function (par, fn, lower = -Inf, upper = Inf, control = list(),  :
#   NAs introduced by coercion to integer range
# 4: In optwrap(optimizer, devfun, start, rho$lower, control = control,  :
#   convergence code 1 from bobyqa: bobyqa -- maximum number of function evaluations exceeded
# 5: In checkConv(attr(opt, "derivs"), opt$par, ctrl = control$checkConv,  :
#   unable to evaluate scaled gradient
# 6: In checkConv(attr(opt, "derivs"), opt$par, ctrl = control$checkConv,  :
#   Model failed to converge: degenerate  Hessian with 2 negative eigenvalues

accmod0e= glmer(correct ~ (1+GVS+Block|Subject), 
               data= ART.accuracy, family= binomial,
               control=glmerControl(optimizer="bobyqa", optCtrl=list(maxfun=10^20)))

# Warning messages:
# 1: In (function (par, fn, lower = -Inf, upper = Inf, control = list(),  :
#   NAs introduced by coercion to integer range
# 2: In optwrap(optimizer, devfun, start, rho$lower, control = control,  :
#   convergence code 1 from bobyqa: bobyqa -- maximum number of function evaluations exceeded
# 3: In (function (par, fn, lower = -Inf, upper = Inf, control = list(),  :
#   NAs introduced by coercion to integer range
# 4: In optwrap(optimizer, devfun, start, rho$lower, control = control,  :
#   convergence code 1 from bobyqa: bobyqa -- maximum number of function evaluations exceeded
# 5: In checkConv(attr(opt, "derivs"), opt$par, ctrl = control$checkConv,  :
#   unable to evaluate scaled gradient
# 6: In checkConv(attr(opt, "derivs"), opt$par, ctrl = control$checkConv,  :
#   Model failed to converge: degenerate  Hessian with 3 negative eigenvalues

accmod0f= glmer(correct ~ (1+GVS+Shape|Subject), 
               data= ART.accuracy, family= binomial,
               control=glmerControl(optimizer="bobyqa", optCtrl=list(maxfun=10^20)))

# Warning messages:
# 1: In (function (par, fn, lower = -Inf, upper = Inf, control = list(),  :
#   NAs introduced by coercion to integer range
# 2: In optwrap(optimizer, devfun, start, rho$lower, control = control,  :
#   convergence code 1 from bobyqa: bobyqa -- maximum number of function evaluations exceeded
# 3: In (function (par, fn, lower = -Inf, upper = Inf, control = list(),  :
#   NAs introduced by coercion to integer range
# 4: In optwrap(optimizer, devfun, start, rho$lower, control = control,  :
#   convergence code 1 from bobyqa: bobyqa -- maximum number of function evaluations exceeded
# 5: In checkConv(attr(opt, "derivs"), opt$par, ctrl = control$checkConv,  :
#   unable to evaluate scaled gradient
# 6: In checkConv(attr(opt, "derivs"), opt$par, ctrl = control$checkConv,  :
#   Model failed to converge: degenerate  Hessian with 2 negative eigenvalues
```

Only the random slopes of GVS was flawlessly included; other terms resulted in convergence problems already… The first attempt is thus:

```
# acc_models= mixed(correct ~ GVS*Reward*Validity*Side*Block +
#                  (1+GVS|Subject), family= binomial,
#                expand_re = F, all_fit = T, data= ART.accuracy, method= "LRT",  type= "2",
#                control= glmerControl(optimizer="bobyqa", optCtrl=list(maxfun=10^20)))
```

There was no way this could finish. I tried several times but after ~ 7 days R would choke… The binomial link is known to be slower and more prone to convergence problems, especially in our case (few cells in few subjects are at ceiling). We shall simplify:

```
#memory.limit(10e12)
acc_models2= mixed(correct ~ GVS*Reward*Validity*Side*Block +
                   (1|Subject), family= binomial,
                   all_fit = T, data= ART.accuracy, method= "LRT",  type= "2",
                   control= glmerControl(optimizer="bobyqa", optCtrl=list(maxfun=10^20)))
```

This worked (and took ~5 days on an average machine (4Gb RAM, 2.8GHz processor). Controls:

```
lapply(acc_models2$restricted_models, function(x) x@optinfo$warnings)
```

```
## $GVS
## list()
## 
## $Reward
## list()
## 
## $Validity
## list()
## 
## $Side
## list()
## 
## $Block
## list()
## 
## $`GVS:Reward`
## list()
## 
## $`GVS:Validity`
## list()
## 
## $`Reward:Validity`
## list()
## 
## $`GVS:Side`
## list()
## 
## $`Reward:Side`
## list()
## 
## $`Validity:Side`
## list()
## 
## $`GVS:Block`
## list()
## 
## $`Reward:Block`
## list()
## 
## $`Validity:Block`
## list()
## 
## $`Side:Block`
## list()
## 
## $`GVS:Reward:Validity`
## list()
## 
## $`GVS:Reward:Side`
## list()
## 
## $`GVS:Validity:Side`
## list()
## 
## $`Reward:Validity:Side`
## list()
## 
## $`GVS:Reward:Block`
## list()
## 
## $`GVS:Validity:Block`
## list()
## 
## $`Reward:Validity:Block`
## list()
## 
## $`GVS:Side:Block`
## list()
## 
## $`Reward:Side:Block`
## list()
## 
## $`Validity:Side:Block`
## list()
## 
## $`GVS:Reward:Validity:Side`
## list()
## 
## $`GVS:Reward:Validity:Block`
## list()
## 
## $`GVS:Reward:Side:Block`
## list()
## 
## $`GVS:Validity:Side:Block`
## list()
## 
## $`Reward:Validity:Side:Block`
## list()
## 
## $`GVS:Reward:Validity:Side:Block`
## list()
```

```
lapply(acc_models2$restricted_models, function(x) x@optinfo$conv) %>% unlist
```

```
##                            GVS.opt                         Reward.opt 
##                                  0                                  0 
##                       Validity.opt                           Side.opt 
##                                  0                                  0 
##                          Block.opt                     GVS:Reward.opt 
##                                  0                                  0 
##                   GVS:Validity.opt                Reward:Validity.opt 
##                                  0                                  0 
##                       GVS:Side.opt                    Reward:Side.opt 
##                                  0                                  0 
##                  Validity:Side.opt                      GVS:Block.opt 
##                                  0                                  0 
##                   Reward:Block.opt                 Validity:Block.opt 
##                                  0                                  0 
##                     Side:Block.opt            GVS:Reward:Validity.opt 
##                                  0                                  0 
##                GVS:Reward:Side.opt              GVS:Validity:Side.opt 
##                                  0                                  0 
##           Reward:Validity:Side.opt               GVS:Reward:Block.opt 
##                                  0                                  0 
##             GVS:Validity:Block.opt          Reward:Validity:Block.opt 
##                                  0                                  0 
##                 GVS:Side:Block.opt              Reward:Side:Block.opt 
##                                  0                                  0 
##            Validity:Side:Block.opt       GVS:Reward:Validity:Side.opt 
##                                  0                                  0 
##      GVS:Reward:Validity:Block.opt          GVS:Reward:Side:Block.opt 
##                                  0                                  0 
##        GVS:Validity:Side:Block.opt     Reward:Validity:Side:Block.opt 
##                                  0                                  0 
## GVS:Reward:Validity:Side:Block.opt 
##                                  0
```

```
sapply(acc_models2$restricted_models, inherits, "try-error")
```

```
##                            GVS                         Reward 
##                          FALSE                          FALSE 
##                       Validity                           Side 
##                          FALSE                          FALSE 
##                          Block                     GVS:Reward 
##                          FALSE                          FALSE 
##                   GVS:Validity                Reward:Validity 
##                          FALSE                          FALSE 
##                       GVS:Side                    Reward:Side 
##                          FALSE                          FALSE 
##                  Validity:Side                      GVS:Block 
##                          FALSE                          FALSE 
##                   Reward:Block                 Validity:Block 
##                          FALSE                          FALSE 
##                     Side:Block            GVS:Reward:Validity 
##                          FALSE                          FALSE 
##                GVS:Reward:Side              GVS:Validity:Side 
##                          FALSE                          FALSE 
##           Reward:Validity:Side               GVS:Reward:Block 
##                          FALSE                          FALSE 
##             GVS:Validity:Block          Reward:Validity:Block 
##                          FALSE                          FALSE 
##                 GVS:Side:Block              Reward:Side:Block 
##                          FALSE                          FALSE 
##            Validity:Side:Block       GVS:Reward:Validity:Side 
##                          FALSE                          FALSE 
##      GVS:Reward:Validity:Block          GVS:Reward:Side:Block 
##                          FALSE                          FALSE 
##        GVS:Validity:Side:Block     Reward:Validity:Side:Block 
##                          FALSE                          FALSE 
## GVS:Reward:Validity:Side:Block 
##                          FALSE
```

```
sapply(acc_models2$restricted_models, function(x) {
  with(x@optinfo$derivs, solve(Hessian,gradient)) %>% abs %>% max %>% return})
```

```
##                            GVS                         Reward 
##                   3.178615e-06                   3.963313e-06 
##                       Validity                           Side 
##                   1.394694e-06                   4.108480e-06 
##                          Block                     GVS:Reward 
##                   4.847984e-06                   8.820994e-06 
##                   GVS:Validity                Reward:Validity 
##                   1.206689e-05                   1.225490e-05 
##                       GVS:Side                    Reward:Side 
##                   2.121599e-05                   1.369041e-05 
##                  Validity:Side                      GVS:Block 
##                   1.814635e-05                   8.850701e-06 
##                   Reward:Block                 Validity:Block 
##                   1.299140e-05                   1.564201e-05 
##                     Side:Block            GVS:Reward:Validity 
##                   8.360774e-06                   2.409852e-05 
##                GVS:Reward:Side              GVS:Validity:Side 
##                   4.240444e-05                   1.915471e-05 
##           Reward:Validity:Side               GVS:Reward:Block 
##                   6.380114e-05                   3.089985e-05 
##             GVS:Validity:Block          Reward:Validity:Block 
##                   2.511426e-05                   3.386553e-05 
##                 GVS:Side:Block              Reward:Side:Block 
##                   4.186611e-05                   3.082560e-05 
##            Validity:Side:Block       GVS:Reward:Validity:Side 
##                   2.709794e-05                   7.901250e-05 
##      GVS:Reward:Validity:Block          GVS:Reward:Side:Block 
##                   6.655053e-05                   6.175511e-05 
##        GVS:Validity:Side:Block     Reward:Validity:Side:Block 
##                   7.015879e-05                   1.033167e-04 
## GVS:Reward:Validity:Side:Block 
##                   5.787245e-05
```

It seems all good, at least!

```
acc_models2 %>% nice
```

| Effect | df | Chisq | p.value |
| --- | --- | --- | --- |
| GVS | 2 | 0.09 | .96 |
| Reward | 2 | 4.31 | .12 |
| Validity | 1 | 0.16 | .69 |
| Side | 1 | 17.70 \*\*\* | <.0001 |
| Block | 2 | 8.68 \* | .01 |
| GVS:Reward | 4 | 2.13 | .71 |
| GVS:Validity | 2 | 0.21 | .90 |
| Reward:Validity | 2 | 1.41 | .49 |
| GVS:Side | 2 | 2.88 | .24 |
| Reward:Side | 2 | 0.34 | .85 |
| Validity:Side | 1 | 1.66 | .20 |
| GVS:Block | 4 | 1.92 | .75 |
| Reward:Block | 4 | 7.78 + | .10 |
| Validity:Block | 2 | 1.07 | .59 |
| Side:Block | 2 | 2.02 | .36 |
| GVS:Reward:Validity | 4 | 7.27 | .12 |
| GVS:Reward:Side | 4 | 7.06 | .13 |
| GVS:Validity:Side | 2 | 0.95 | .62 |
| Reward:Validity:Side | 2 | 2.04 | .36 |
| GVS:Reward:Block | 8 | 5.52 | .70 |
| GVS:Validity:Block | 4 | 2.61 | .62 |
| Reward:Validity:Block | 4 | 6.01 | .20 |
| GVS:Side:Block | 4 | 2.72 | .61 |
| Reward:Side:Block | 4 | 2.45 | .65 |
| Validity:Side:Block | 2 | 2.28 | .32 |
| GVS:Reward:Validity:Side | 4 | 1.48 | .83 |
| GVS:Reward:Validity:Block | 8 | 6.48 | .59 |
| GVS:Reward:Side:Block | 8 | 5.72 | .68 |
| GVS:Validity:Side:Block | 4 | 1.79 | .77 |
| Reward:Validity:Side:Block | 4 | 3.42 | .49 |
| GVS:Reward:Validity:Side:Block | 8 | 7.18 | .52 |

However, there results are uncorrected for multiple testing. We declared our interesting tests to be: Reward, Reward by Validity, GVS by Reward, and GVS by Reward by Validity. We apply a fdr correction to these values and to the others separately:

```
interesting_tests= c("Reward", "GVS:Reward", "Reward:Validity", "GVS:Reward:Validity")
acc_models2$anova_table$p_fdr= NA

acc_models2$anova_table[row.names(acc_models2$anova_table) %in% interesting_tests, ]$p_fdr=
p.adjust(acc_models2$anova_table[row.names(acc_models2$anova_table) %in% interesting_tests, ]$`Pr(>Chisq)`, "fdr")

acc_models2$anova_table[!row.names(acc_models2$anova_table) %in% interesting_tests, ]$p_fdr=
p.adjust(acc_models2$anova_table[!row.names(acc_models2$anova_table) %in% interesting_tests, ]$`Pr(>Chisq)`, "fdr")

cbind(acc_models2$anova_table, Sig= as.character(ifelse(acc_models2$anova_table$p_fdr<0.043, "*", " ")))
```

|  | Df | Chisq | Chi Df | Pr(>Chisq) | p\_fdr | Sig |
| --- | --- | --- | --- | --- | --- | --- |
| GVS | 8 | 0.0872185 | 2 | 0.9573280 | 0.9573280 |  |
| Reward | 8 | 4.3054325 | 2 | 0.1161682 | 0.2450145 |  |
| Validity | 9 | 0.1554629 | 1 | 0.6933686 | 0.9007904 |  |
| Side | 9 | 17.6981215 | 1 | 0.0000259 | 0.0006990 | \* |
| Block | 8 | 8.6823848 | 2 | 0.0130210 | 0.1757834 |  |
| GVS:Reward | 31 | 2.1328695 | 4 | 0.7113363 | 0.7113363 |  |
| GVS:Validity | 33 | 0.2123173 | 2 | 0.8992820 | 0.9338698 |  |
| Reward:Validity | 33 | 1.4093842 | 2 | 0.4942607 | 0.6590143 |  |
| GVS:Side | 33 | 2.8818047 | 2 | 0.2367141 | 0.9007904 |  |
| Reward:Side | 33 | 0.3351632 | 2 | 0.8457076 | 0.9133642 |  |
| Validity:Side | 34 | 1.6609483 | 1 | 0.1974753 | 0.8928443 |  |
| GVS:Block | 31 | 1.9239217 | 4 | 0.7497493 | 0.9086628 |  |
| Reward:Block | 31 | 7.7816337 | 4 | 0.0999128 | 0.8928443 |  |
| Validity:Block | 33 | 1.0711941 | 2 | 0.5853197 | 0.9007904 |  |
| Side:Block | 33 | 2.0194867 | 2 | 0.3643125 | 0.9007904 |  |
| GVS:Reward:Validity | 69 | 7.2654633 | 4 | 0.1225073 | 0.2450145 |  |
| GVS:Reward:Side | 69 | 7.0582824 | 4 | 0.1328401 | 0.8928443 |  |
| GVS:Validity:Side | 71 | 0.9455841 | 2 | 0.6232597 | 0.9007904 |  |
| Reward:Validity:Side | 71 | 2.0404990 | 2 | 0.3605050 | 0.9007904 |  |
| GVS:Reward:Block | 65 | 5.5218792 | 8 | 0.7006148 | 0.9007904 |  |
| GVS:Validity:Block | 69 | 2.6107171 | 4 | 0.6249258 | 0.9007904 |  |
| Reward:Validity:Block | 69 | 6.0099042 | 4 | 0.1984098 | 0.8928443 |  |
| GVS:Side:Block | 69 | 2.7203744 | 4 | 0.6056541 | 0.9007904 |  |
| Reward:Side:Block | 69 | 2.4517770 | 4 | 0.6532912 | 0.9007904 |  |
| Validity:Side:Block | 71 | 2.2773021 | 2 | 0.3202507 | 0.9007904 |  |
| GVS:Reward:Validity:Side | 97 | 1.4809892 | 4 | 0.8300036 | 0.9133642 |  |
| GVS:Reward:Validity:Block | 93 | 6.4783894 | 8 | 0.5938057 | 0.9007904 |  |
| GVS:Reward:Side:Block | 93 | 5.7237768 | 8 | 0.6781411 | 0.9007904 |  |
| GVS:Validity:Side:Block | 97 | 1.7914511 | 4 | 0.7740460 | 0.9086628 |  |
| Reward:Validity:Side:Block | 97 | 3.4184836 | 4 | 0.4903808 | 0.9007904 |  |
| GVS:Reward:Validity:Side:Block | 101 | 7.1818721 | 8 | 0.5171434 | 0.9007904 |  |

Only the two main effects of Side and Block were significant at the uncorrected level; only the main effect of Side is significant at the fdr corrected level. Side:

```
lsm.options(lmer.df = "asymptotic") # the fastest, no df
lsmeans(acc_models2, pairwise ~ Side, adjust= "fdr")$contrast
```

```
## lsmeans are based on full model which includes all effects.
```

```
## NOTE: Results may be misleading due to involvement in interactions
```

```
##  contrast       estimate         SE df z.ratio p.value
##  Left - Right -0.1941313 0.04472449 NA  -4.341  <.0001
## 
## Results are averaged over the levels of: GVS, Reward, Validity, Block 
## Results are given on the log odds ratio (not the response) scale.
```

The accuracy was higher for stimuli presented in the right side of space (coherent with RTs). Block:

```
lsmeans(acc_models2, pairwise ~ Block, adjust= "fdr")$contrast
```

```
## lsmeans are based on full model which includes all effects.
```

```
## NOTE: Results may be misleading due to involvement in interactions
```

```
##  contrast   estimate         SE df z.ratio p.value
##  1 - 2    -0.1188089 0.05381469 NA  -2.208  0.0409
##  1 - 3    -0.1636818 0.05455856 NA  -3.000  0.0081
##  2 - 3    -0.0448729 0.05594701 NA  -0.802  0.4225
## 
## Results are averaged over the levels of: GVS, Reward, Validity, Side 
## Results are given on the log odds ratio (not the response) scale. 
## P value adjustment: fdr method for 3 tests
```

Block was only significant at the uncorrected threshold: this was due to accuracy increasing from block 1 to blocks 2 and 3, with no differences between the last two blocks.

### Plots

Here’s a function to plot data, with option to display variability and data distribution:

```
my_plot= function(DF, dv= c("correct", "response_time"), withinvars, betweenvars= NULL, 
                  idvar= "Subject", 
                  individual_data= c("none", "p", "l", "violin", "bp"),
                  plot.gain= T){
  
  #match arguments
  individual_data= match.arg(individual_data)
  dv= match.arg(dv)
  
  #concatenate all relevant variables
  allvars= c(withinvars, betweenvars)
  
  #summarise data according to grouping variables
  Xs= ddply(DF, c(allvars, idvar), summarise, y= mean(get(dv)))
  
  #set axis label
  if(dv=="correct")(ylab= "Accuracy") else (ylab= "Reaction times (ms)")
  
  #if Validity is involved, summarise the deltas
  #code everything as "gain", so it changes for rts and accuracy (positive diff= gain)
  if("Validity" %in% allvars & plot.gain== T){
    
    print("Validity is involved: plotting deltas")
    
    if(dv== "correct"){
    X.temp= subset(Xs, Validity== "Valid") 
    X.temp$y= X.temp$y - subset(Xs, Validity== "Invalid")$y} else {
      X.temp= subset(Xs, Validity== "Invalid") 
      X.temp$y= X.temp$y - subset(Xs, Validity== "Valid")$y}

    Xs= X.temp[, !names(X.temp) %in% "Validity"]
    
    withinvars= withinvars[!withinvars %in% "Validity"]
    allvars= allvars[!allvars %in% "Validity"]
     
    if(dv=="correct")(ylab= "Accuracy (gain)") else (ylab= "Reaction times (gain, ms)")
    }
  
  Xs[, idvar]= as.factor(Xs[, idvar])
  
  #obtain summary statistics
  X= summarySEwithin(Xs, measurevar = "y", 
                     betweenvars = betweenvars, 
                     withinvars = withinvars, idvar = idvar)

  #finally depict!
  p= ggplot(X, aes(y= y, x= get(allvars[1]), fill= get(allvars[1]))) + commonTheme 
  
  #if it's a delta plot line @ 0
  if(grepl("gain", ylab))(p= p + geom_hline(yintercept = 0, linetype= "dashed", size= 1.1,
                                            color= ifelse(individual_data=="l", "darkgray", "gray")))
  
  #if data distribution is asked for
  if(individual_data=="l"){
    p= p + geom_line(data = Xs, aes(x= get(allvars[1]), y=  y, group= get(idvar)), 
                     size= 1.2, color= "dark gray", alpha= 0.5)} 
  
  if(individual_data=="p"){
    pd= position_dodge(0.5)
    p= p + geom_point(data = Xs, aes(x= get(allvars[1]), y=  y, group= get(idvar)),
                      position = pd, size= 3, shape= 21, color= "black", alpha= 0.3)} 
  
  if(individual_data=="violin"){
    p= p + geom_violin(data = Xs, aes(x= get(allvars[1]), y=  y), alpha= 0.3)} 
  
  if(individual_data=="bp"){
    p= p + geom_boxplot(data = Xs, aes(x= get(allvars[1]), y=  y), alpha= 0.3, 
                        outlier.size = 3.5)} 
  
  #plot summary statistics
  if(individual_data=="none"){
    p= p + geom_errorbar(data=X, aes(ymin= y-se, ymax= y+se), 
                       size= 1.5, width= .2, colour= "black") + 
    geom_point(size= 6, stroke= 2, shape= 21, color= "black")
  }
  
   
  p= p + labs(y= ylab, x= allvars[1]) + guides(fill=F)
  
  #for many variables at once, wrapping
  if(length(allvars)>1) (p= p + facet_wrap(allvars[2:length(allvars)]))
  
  #final plot
  return(p)  
  
}
```

The only (very) robust effect was that of Side:

```
my_plot(ART.accuracy, dv <- "correct", withinvars = c("Side"))
```

```
## Automatically converting the following non-factors to factors: Side
```

And then Block (uncorrected level):

```
my_plot(ART.accuracy, dv <- "correct", withinvars = c("Block"))
```

Suggesting improvements after the first block. However, just to be 100% sure there were no trade-offs between accuracy and RTs, we depict all our tests of interest. Reward:

```
my_plot(ART.accuracy, dv <- "correct", withinvars = c("Reward"))
```

It was not significant but a trend is seen for high rewards to boost the performance (accuracy). Thus, we can tease apart the possibility of a speed-accuracy trade-off. The two-way Reward by Validity:

```
my_plot(ART.accuracy, dv <- "correct", withinvars = c("Reward", "Validity"))
```

```
## [1] "Validity is involved: plotting deltas"
```

It was not significant. The tendency for accuracy is for rewards to reduce IOR or even result into validity gain (though note the scale of the plot, differences are truly minor). Of interest, given results for RTs, is the two-way GVS by Reward:

```
my_plot(ART.accuracy, dv <- "correct", withinvars = c("Reward", "GVS"))
```

For RTs we have found a reduction of the benefical effect of rewards induced by GVS (especially Right-Anodal). There were no significant effects here, and indeed the differences are very small, yet the pattern resembles a bit what found for RTs (the effect of rewards in the SHAM is steeper, more evident, than in both GVS conditions). Again, this speaks against a possible trade-off.

Finally, the three-way interaction with Validity:

```
my_plot(ART.accuracy, dv <- "correct", withinvars = c("Reward", "GVS", "Validity"))
```

```
## [1] "Validity is involved: plotting deltas"
```

Was not significant. It is very interesting though to notice the expected pattern for SHAM (rewards enhance IOR), but the opposite for both GVS conditions!

## Appendix

This is the function to summarise data (`summarySEwithin`):

```
## Summarizes data.
## Gives count, mean, standard deviation, standard error of the mean, and confidence interval (default 95%).
##   data: a data frame.
##   measurevar: the name of a column that contains the variable to be summariezed
##   groupvars: a vector containing names of columns that contain grouping variables
##   na.rm: a boolean that indicates whether to ignore NA's
##   conf.interval: the percent range of the confidence interval (default is 95%)
summarySE <- function(data=NULL, measurevar, groupvars=NULL, na.rm=FALSE,
                      conf.interval=.95, .drop=TRUE) {
  library(plyr)
  
  # New version of length which can handle NA's: if na.rm==T, don't count them
  length2 <- function (x, na.rm=FALSE) {
    if (na.rm) sum(!is.na(x))
    else       length(x)
  }
  
  # This does the summary. For each group's data frame, return a vector with
  # N, mean, and sd
  datac <- ddply(data, groupvars, .drop=.drop,
                 .fun = function(xx, col) {
                   c(N    = length2(xx[[col]], na.rm=na.rm),
                     mean = mean   (xx[[col]], na.rm=na.rm),
                     sd   = sd     (xx[[col]], na.rm=na.rm)
                   )
                 },
                 measurevar
  )
  
  # Rename the "mean" column    
  datac <- plyr::rename(datac, c(mean = measurevar))
  
  datac$se <- datac$sd / sqrt(datac$N)  # Calculate standard error of the mean
  
  # Confidence interval multiplier for standard error
  # Calculate t-statistic for confidence interval: 
  # e.g., if conf.interval is .95, use .975 (above/below), and use df=N-1
  ciMult <- qt(conf.interval/2 + .5, datac$N-1)
  datac$ci <- datac$se * ciMult
  
  return(datac)
}


## Norms the data within specified groups in a data frame; it normalizes each
## subject (identified by idvar) so that they have the same mean, within each group
## specified by betweenvars.
##   data: a data frame.
##   idvar: the name of a column that identifies each subject (or matched subjects)
##   measurevar: the name of a column that contains the variable to be summariezed
##   betweenvars: a vector containing names of columns that are between-subjects variables
##   na.rm: a boolean that indicates whether to ignore NA's
normDataWithin <- function(data=NULL, idvar, measurevar, betweenvars=NULL,
                           na.rm=FALSE, .drop=TRUE) {
  library(plyr)
  
  # Measure var on left, idvar + between vars on right of formula.
  data.subjMean <- ddply(data, c(idvar, betweenvars), .drop=.drop,
                         .fun = function(xx, col, na.rm) {
                           c(subjMean = mean(xx[,col], na.rm=na.rm))
                         },
                         measurevar,
                         na.rm
  )
  
  # Put the subject means with original data
  data <- merge(data, data.subjMean)
  
  # Get the normalized data in a new column
  measureNormedVar <- paste(measurevar, "_norm", sep="")
  data[,measureNormedVar] <- data[,measurevar] - data[,"subjMean"] +
    mean(data[,measurevar], na.rm=na.rm)
  
  # Remove this subject mean column
  data$subjMean <- NULL
  
  return(data)
}


## Retrieved here: http://www.cookbook-r.com/Graphs/Plotting_means_and_error_bars_(ggplot2)/
## Summarizes data, handling within-subjects variables by removing inter-subject variability.
## It will still work if there are no within-S variables.
## Gives count, un-normed mean, normed mean (with same between-group mean),
##   standard deviation, standard error of the mean, and confidence interval.
## If there are within-subject variables, calculate adjusted values using method from Morey (2008).
##   data: a data frame.
##   measurevar: the name of a column that contains the variable to be summariezed
##   betweenvars: a vector containing names of columns that are between-subjects variables
##   withinvars: a vector containing names of columns that are within-subjects variables
##   idvar: the name of a column that identifies each subject (or matched subjects)
##   na.rm: a boolean that indicates whether to ignore NA's
##   conf.interval: the percent range of the confidence interval (default is 95%)
summarySEwithin <- function(data=NULL, measurevar, betweenvars=NULL, withinvars=NULL,
                            idvar=NULL, na.rm=FALSE, conf.interval=.95, .drop=TRUE) {
  
  # Ensure that the betweenvars and withinvars are factors
  factorvars <- vapply(data[, c(betweenvars, withinvars), drop=FALSE],
                       FUN=is.factor, FUN.VALUE=logical(1))
  
  if (!all(factorvars)) {
    nonfactorvars <- names(factorvars)[!factorvars]
    message("Automatically converting the following non-factors to factors: ",
            paste(nonfactorvars, collapse = ", "))
    data[nonfactorvars] <- lapply(data[nonfactorvars], factor)
  }
  
  # Get the means from the un-normed data
  datac <- summarySE(data, measurevar, groupvars=c(betweenvars, withinvars),
                     na.rm=na.rm, conf.interval=conf.interval, .drop=.drop)
  
  # Drop all the unused columns (these will be calculated with normed data)
  datac$sd <- NULL
  datac$se <- NULL
  datac$ci <- NULL
  
  # Norm each subject's data
  ndata <- normDataWithin(data, idvar, measurevar, betweenvars, na.rm, .drop=.drop)
  
  # This is the name of the new column
  measurevar_n <- paste(measurevar, "_norm", sep="")
  
  # Collapse the normed data - now we can treat between and within vars the same
  ndatac <- summarySE(ndata, measurevar_n, groupvars=c(betweenvars, withinvars),
                      na.rm=na.rm, conf.interval=conf.interval, .drop=.drop)
  
  # Apply correction from Morey (2008) to the standard error and confidence interval
  #  Get the product of the number of conditions of within-S variables
  nWithinGroups    <- prod(vapply(ndatac[,withinvars, drop=FALSE], FUN=nlevels,
                                  FUN.VALUE=numeric(1)))
  correctionFactor <- sqrt( nWithinGroups / (nWithinGroups-1) )
  
  # Apply the correction factor
  ndatac$sd <- ndatac$sd * correctionFactor
  ndatac$se <- ndatac$se * correctionFactor
  ndatac$ci <- ndatac$ci * correctionFactor
  
  # Combine the un-normed means with the normed results
  merge(datac, ndatac)
}
```
