# Supplementary material for: Probing the role of the vestibular system in motivation and reward-based attention
Source: Cortex. 2018 Jun;103:82–99. doi: 10.1016/j.cortex.2018.02.009 (PMC6002611; doi:10.1016/j.cortex.2018.02.009)
Supplement: Supplementary file 5 [file mmc5.zip › cortex_2252_mmc5.html]

S3: ART task, Robustness check and Exploratory analyses


# S3: ART task, Robustness check and Exploratory analyses

#### *Elvio Blini INSERM U1028, ImpAct team, CRNL, and University of Lyon elvio.blini@gmail.com*

#### *17 November 2017*

**Please refer to the other companion html files (S3, ART accuracy, and S4, ART rts) for more detailed description of the task and pre-processing procedures**

# Robustness check

With preregistered analyses we have provided evidence for the two-way interaction **GVS by Reward**. On the contrary, we failed to provide evidence for the three-way **GVS by Validity by Reward**.

In this document we ran secondary analyses and robustness checks in order to collect converging statistical evidence around the *same* family of tests of interest. Basically, we aim to respond to the same questions we attempted to respond to with the main inferential approach, which was based on mixed linear regression models. After this first step, we will look for data-driven and post-hoc results in a separate section.

The analyses presented in this document, with the exception of Bayesian ANOVAs, have not been pre-registered, thus they ought to be interpreted with caution.

## Preliminary setup

It’s sometimes good to clean the current environment to avoid conflicts. You can do it with `rm(list=ls())` (but be sure everything is properly saved for future use).

In order to run this script we need a few packages available on CRAN. You might need to install them first, e.g. by typing `install.packages("BayesFactor")` in the console.

```
#list packages
packages= c("psych", "plyr", "magrittr", "tidyverse", "BayesFactor", "lme4", "multcomp",
            "gridExtra", "ez" , "lsmeans", "retimes")

#load them
lapply(packages, require, character.only= T)
```

For package `afex` I’m using the developer version on github (you will need to install `devtools`).

```
#devtools::install_github("singmann/afex@master")
library(afex)
```

For reproducibility, here’s details of the system on which the script was tested and a seed for random numbers:

```
set.seed(1)

sessionInfo()
```

```
## R version 3.4.2 (2017-09-28)
## Platform: x86_64-w64-mingw32/x64 (64-bit)
## Running under: Windows 10 x64 (build 15063)
## 
## Matrix products: default
## 
## locale:
## [1] LC_COLLATE=English_United Kingdom.1252 
## [2] LC_CTYPE=English_United Kingdom.1252   
## [3] LC_MONETARY=English_United Kingdom.1252
## [4] LC_NUMERIC=C                           
## [5] LC_TIME=English_United Kingdom.1252    
## 
## attached base packages:
## [1] stats     graphics  grDevices utils     datasets  methods   base     
## 
## other attached packages:
##  [1] afex_0.18-0          retimes_0.1-2        lsmeans_2.27-2      
##  [4] estimability_1.2     ez_4.4-0             gridExtra_2.3       
##  [7] multcomp_1.4-7       TH.data_1.0-8        MASS_7.3-47         
## [10] survival_2.41-3      mvtnorm_1.0-6        lme4_1.1-14         
## [13] BayesFactor_0.9.12-2 Matrix_1.2-11        coda_0.19-1         
## [16] dplyr_0.7.4          purrr_0.2.3          readr_1.1.1         
## [19] tidyr_0.7.1          tibble_1.3.4         ggplot2_2.2.1       
## [22] tidyverse_1.1.1      magrittr_1.5         plyr_1.8.4          
## [25] psych_1.7.8          printr_0.1          
## 
## loaded via a namespace (and not attached):
##  [1] nlme_3.1-131        pbkrtest_0.4-7      lubridate_1.6.0    
##  [4] RColorBrewer_1.1-2  httr_1.3.1          rprojroot_1.2      
##  [7] tools_3.4.2         backports_1.1.1     R6_2.2.2           
## [10] rpart_4.1-11        Hmisc_4.0-3         lazyeval_0.2.0     
## [13] mgcv_1.8-20         colorspace_1.3-2    nnet_7.3-12        
## [16] mnormt_1.5-5        compiler_3.4.2      rvest_0.3.2        
## [19] quantreg_5.33       htmlTable_1.9       SparseM_1.77       
## [22] xml2_1.1.1          sandwich_2.4-0      checkmate_1.8.4    
## [25] scales_0.5.0        pbapply_1.3-3       stringr_1.2.0      
## [28] digest_0.6.12       foreign_0.8-69      minqa_1.2.4        
## [31] rmarkdown_1.7       base64enc_0.1-3     pkgconfig_2.0.1    
## [34] htmltools_0.3.6     htmlwidgets_0.9     rlang_0.1.2        
## [37] readxl_1.0.0        bindr_0.1           zoo_1.8-0          
## [40] jsonlite_1.5        gtools_3.5.0        acepack_1.4.1      
## [43] car_2.1-5           modeltools_0.2-21   Formula_1.2-2      
## [46] Rcpp_0.12.13        munsell_0.4.3       stringi_1.1.5      
## [49] yaml_2.1.14         grid_3.4.2          parallel_3.4.2     
## [52] forcats_0.2.0       lattice_0.20-35     haven_1.1.0        
## [55] splines_3.4.2       hms_0.3             knitr_1.17         
## [58] reshape2_1.4.2      codetools_0.2-15    stats4_3.4.2       
## [61] glue_1.1.1          evaluate_0.10.1     latticeExtra_0.6-28
## [64] data.table_1.10.4   modelr_0.1.1        nloptr_1.0.4       
## [67] MatrixModels_0.4-1  cellranger_1.1.0    gtable_0.2.0       
## [70] assertthat_0.2.0    coin_1.2-1          xtable_1.8-2       
## [73] broom_0.4.2         lmerTest_2.0-33     bindrcpp_0.2       
## [76] cluster_2.0.6
```

Thanks to the function retrieved here, not displayed, the following hyperlinks download the Rdata files:

That can be loaded then with:

```
load("ART data.RData")
```

Now all relevant variables are stored in the `ART` data.frame, that you can navigate and explore with the usual commands, e.g. `str(ART)`.

You can do the same for SVV and EST data frames, that we will use at the end of this file for correlations.

```
load("SVV data.RData")
```

Then the questionnaire:

```
load("EST data.RData")
```

We now have all the data produced by this study in the R environment!

## Useful functions

This is a minimal (shared by all plots) list of graphic attributes for ggplot:

```
#ggplot defaults
commonTheme = list(theme_bw(),
                   theme(text = element_text(size=18, face="bold"),
                         axis.text = element_text(size=16, face="bold", color= "black"),
                         plot.margin = unit(c(1,1,1,1), "cm")))
```

Then (ONLY SHOWN IN THE BOTTOM PART OF THIS SCRIPT) we have a convenient summarising function that produces means and within subjects SEM as in Morey (2008). I retrieved it here: link

Finally, this is a convenience function to plot various results:

```
my_plot= function(DF, dv= c("correct", "response_time"), withinvars, betweenvars= NULL, 
                  idvar= "Subject", 
                  individual_data= c("none", "p", "l", "violin", "bp"),
                  plot.gain= T){
  
  #match arguments
  individual_data= match.arg(individual_data)
  dv= match.arg(dv)
  
  #concatenate all relevant variables
  allvars= c(withinvars, betweenvars)
  
  #summarise data according to grouping variables
  Xs= ddply(DF, c(allvars, idvar), summarise, y= mean(get(dv)))
  
  #set axis label
  if(dv=="correct")(ylab= "Accuracy") else (ylab= "Reaction times (ms)")
  
  #if Validity is involved, summarise the deltas
  #code everything as "gain", so it changes for rts and accuracy (positive diff= gain)
  if("Validity" %in% allvars & plot.gain== T){
    
    print("Validity is involved: plotting deltas")
    
    if(dv== "correct"){
    X.temp= subset(Xs, Validity== "Valid") 
    X.temp$y= X.temp$y - subset(Xs, Validity== "Invalid")$y} else {
      X.temp= subset(Xs, Validity== "Invalid") 
      X.temp$y= X.temp$y - subset(Xs, Validity== "Valid")$y}

    Xs= X.temp[, !names(X.temp) %in% "Validity"]
    
    withinvars= withinvars[!withinvars %in% "Validity"]
    allvars= allvars[!allvars %in% "Validity"]
     
    if(dv=="correct")(ylab= "Accuracy (gain)") else (ylab= "Reaction times (gain, ms)")
    }
  
  Xs[, idvar]= as.factor(Xs[, idvar])
  
  #obtain summary statistics
  X= summarySEwithin(Xs, measurevar = "y", 
                     betweenvars = betweenvars, 
                     withinvars = withinvars, idvar = idvar)

  #finally depict!
  p= ggplot(X, aes(y= y, x= get(allvars[1]), fill= get(allvars[1]))) + commonTheme 
  
  #if it's a delta plot line @ 0
  if(grepl("gain", ylab))(p= p + geom_hline(yintercept = 0, linetype= "dashed", size= 1.1,
                                            color= ifelse(individual_data=="l", "darkgray", "gray")))
  
  #if data distribution is asked for
  if(individual_data=="l"){
    p= p + geom_line(data = Xs, aes(x= get(allvars[1]), y=  y, group= get(idvar)), 
                     size= 1.2, color= "dark gray", alpha= 0.5)} 
  
  if(individual_data=="p"){
    pd= position_dodge(0.5)
    p= p + geom_point(data = Xs, aes(x= get(allvars[1]), y=  y, group= get(idvar)),
                      position = pd, size= 3, shape= 21, color= "black", alpha= 0.3)} 
  
  if(individual_data=="violin"){
    p= p + geom_violin(data = Xs, aes(x= get(allvars[1]), y=  y), alpha= 0.3)} 
  
  if(individual_data=="bp"){
    p= p + geom_boxplot(data = Xs, aes(x= get(allvars[1]), y=  y), alpha= 0.3, 
                        outlier.size = 3.5)} 
  
  #plot summary statistics
  if(individual_data=="none"){
    p= p + geom_errorbar(data=X, aes(ymin= y-se, ymax= y+se), 
                       size= 1.5, width= .2, colour= "black") + 
    geom_point(size= 6, stroke= 2, shape= 21, color= "black")
  }
  
   
  p= p + labs(y= ylab, x= allvars[1]) + guides(fill=F)
  
  #for many variables at once, wrapping
  if(length(allvars)>1) (p= p + facet_wrap(allvars[2:length(allvars)]))
  
  #final plot
  return(p)  
  
}
```

## Preprocessing

Preprocessing procedures are identical to those preregistered. Please refer to the main html file for the ART task for more detailed information.  
A bunch of variables is to declare (e.g., numbers into factors). Then, for the sake of clarity, levels are renamed when helpful.

```
#declare
ART$Subject= as.factor(ART$Subject)

if(sum(levels(as.factor(ART$Reward))== c("0", "2", "10"))==3){
  ART$Reward= factor(ART$Reward, labels = c("None", "Small", "High"))} else 
    (warning("levels unordered? Check"))

if(sum(levels(as.factor(ART$GVS))== c("A", "B", "C"))==3){
  ART$GVS= factor(ART$GVS, labels = c("SHAM", "Left-Anodal", "Right-Anodal"))
  ART$GVS= relevel(ART$GVS, "Left-Anodal")} else (warning("levels unordered? Check"))
```

We must drop a few subjects ( :( ). Reasons are in the comments.

```
#subject 1 to replace for a bug in the script, my fault, :(
ART= ART[!ART$Subject== 1, ] #replaced by subject 51

#subject 10 didn't show up after first session, >:(
ART= ART[!ART$Subject== 10, ] #replaced by subject 60

ART$Subject= factor(ART$Subject)
```

We create a “Block” variable.

```
#insert variable "block"
ART$Block= ifelse(ART$Trial< 145, 1, 
                  ifelse(ART$Trial> 288, 3, 2)) %>% as.factor
```

A valid response was defined as being correct, slower than 100ms, and faster than 500ms.

```
#exclusion criteria applies to valid responses
ART$Valid_Response= ifelse(ART$correct== 1 &
                           ART$response_time> 100 &
                           ART$response_time< 500, 1, 0)
```

We can save a separate dataframe including incorrect responses, and a second one with only valid ones, this way we will work with reaction times for valid responses only:

```
#save data for follow-up analyses and plots (before excluding wrong responses)
ART.accuracy= ART

#exclude bad responses
ART= ART[ART$Valid_Response==1, ]
```

We can start performing our robustness checks!

## Frequentist ANOVA

We can start with a simple ANOVA. First we use `ddply()` to collapse and summarise data, then we use the function `ezANOVA` to run the analysis.

```
EZ= ddply(ART, c("GVS", "Reward", "Validity", "Side", "Block", "Subject"), 
          summarise, dv= mean(response_time)) %>%
          ezANOVA(dv= .(dv), wid = .(Subject), within = c("GVS", "Reward", "Validity", "Side", "Block"), 
                  detailed=T, type = 2)
```

```
## Warning: Converting "Validity" to factor for ANOVA.
```

```
## Warning: Converting "Side" to factor for ANOVA.
```

We apply Greenhouse-Geisser correction where necessary and then correct for multiple tested factors as outlined in the paper and main analysis.

```
#tests needing sphericity corrections:
EZ$`Mauchly's Test for Sphericity`$Effect[EZ$`Mauchly's Test for Sphericity`$p <0.05]
```

```
## [1] "Reward"                         "GVS:Block"                     
## [3] "Reward:Side:Block"              "GVS:Reward:Validity:Side:Block"
```

```
#apply Greenhouse-Geisser correction if necessary
EZ$ANOVA$p[EZ$ANOVA$Effect %in% EZ$`Mauchly's Test for Sphericity`$Effect[EZ$`Mauchly's Test for Sphericity`$p <0.05]] <-
EZ$`Sphericity Corrections`$`p[GG]`[EZ$`Sphericity Corrections`$Effect %in% EZ$`Mauchly's Test for Sphericity`$Effect[EZ$`Mauchly's Test for Sphericity`$p <0.05]]

#simplify
EZ= EZ$ANOVA

#corrections for multiple testing
interesting_tests= c("Reward", "GVS:Reward", "Reward:Validity", "GVS:Reward:Validity")

EZ$p_fdr= NA

#family of tests of interest 
EZ[EZ$Effect %in% interesting_tests, ]$p_fdr=
p.adjust(EZ[EZ$Effect %in% interesting_tests, ]$p, "fdr")

#second family of tests
EZ[!EZ$Effect %in% interesting_tests, ]$p_fdr=
p.adjust(EZ[!EZ$Effect %in% interesting_tests, ]$p, "fdr")
```

The `ez` package by default returns the generalized eta squared. Because more established we switch to the partial eta squared.

```
EZ$pes= EZ$SSn/(EZ$SSn + EZ$SSd)
```

Keeping in mind that our alpha threshold was set to 0.043 due to multiple longitudinal data peeks, here’s the results:

```
EZ$p_fdr_sig= ifelse(EZ$p_fdr<0.043, "*", "")
EZ
```

| Effect | DFn | DFd | SSn | SSd | F | p | p<.05 | ges | p\_fdr | pes | p\_fdr\_sig |
| --- | --- | --- | --- | --- | --- | --- | --- | --- | --- | --- | --- |
| (Intercept) | 1 | 29 | 4.762113e+08 | 594620.44 | 2.322512e+04 | 0.0000000 | \* | 0.9965437 | 0.0000000 | 0.9987529 | \* |
| GVS | 2 | 58 | 4.250960e+03 | 140258.74 | 8.789316e-01 | 0.4206831 |  | 0.0025672 | 0.6361232 | 0.0294164 |  |
| Reward | 2 | 58 | 3.395922e+04 | 29072.05 | 3.387505e+01 | 0.0000000 | \* | 0.0201470 | 0.0000000 | 0.5387678 | \* |
| Validity | 1 | 29 | 1.849090e+04 | 52013.06 | 1.030964e+01 | 0.0032276 | \* | 0.0110717 | 0.0380368 | 0.2622675 | \* |
| Side | 1 | 29 | 1.185474e+04 | 35334.74 | 9.729450e+00 | 0.0040754 | \* | 0.0071265 | 0.0380368 | 0.2512158 | \* |
| Block | 2 | 58 | 2.452040e+03 | 55498.32 | 1.281285e+00 | 0.2854218 |  | 0.0014824 | 0.5708436 | 0.0423128 |  |
| GVS:Reward | 4 | 116 | 4.496883e+03 | 42647.03 | 3.057882e+00 | 0.0194956 | \* | 0.0027153 | 0.0389912 | 0.0953863 | \* |
| GVS:Validity | 2 | 58 | 4.583726e+02 | 23056.33 | 5.765360e-01 | 0.5650280 |  | 0.0002775 | 0.7533707 | 0.0194930 |  |
| Reward:Validity | 2 | 58 | 7.272197e+02 | 16703.55 | 1.262568e+00 | 0.2905857 |  | 0.0004401 | 0.3147213 | 0.0417205 |  |
| GVS:Side | 2 | 58 | 9.443915e+02 | 18473.66 | 1.482508e+00 | 0.2355438 |  | 0.0005715 | 0.5073251 | 0.0486347 |  |
| Reward:Side | 2 | 58 | 2.377573e+02 | 21682.52 | 3.179963e-01 | 0.7288659 |  | 0.0001439 | 0.9276475 | 0.0108465 |  |
| Validity:Side | 1 | 29 | 1.395363e+03 | 17929.69 | 2.256902e+00 | 0.1438319 |  | 0.0008441 | 0.3661174 | 0.0722049 |  |
| GVS:Block | 4 | 116 | 5.711355e+03 | 80464.28 | 2.058420e+00 | 0.1067697 |  | 0.0034461 | 0.3321724 | 0.0662757 |  |
| Reward:Block | 4 | 116 | 2.492950e+03 | 28385.08 | 2.546956e+00 | 0.0430628 | \* | 0.0015071 | 0.2079359 | 0.0807354 |  |
| Validity:Block | 2 | 58 | 2.023590e+03 | 19677.22 | 2.982338e+00 | 0.0584986 |  | 0.0012237 | 0.2339946 | 0.0932495 |  |
| Side:Block | 2 | 58 | 5.205320e+01 | 15658.45 | 9.640430e-02 | 0.9082420 |  | 0.0000315 | 0.9684346 | 0.0033133 |  |
| GVS:Reward:Validity | 4 | 116 | 7.809296e+02 | 18875.69 | 1.199795e+00 | 0.3147213 |  | 0.0004726 | 0.3147213 | 0.0397286 |  |
| GVS:Reward:Side | 4 | 116 | 8.196145e+02 | 25109.10 | 9.466218e-01 | 0.4397611 |  | 0.0004960 | 0.6361232 | 0.0316103 |  |
| GVS:Validity:Side | 2 | 58 | 4.315930e+02 | 13897.00 | 9.006401e-01 | 0.4119152 |  | 0.0002612 | 0.6361232 | 0.0301211 |  |
| Reward:Validity:Side | 2 | 58 | 1.168359e+03 | 16056.72 | 2.110170e+00 | 0.1304286 |  | 0.0007069 | 0.3652000 | 0.0678290 |  |
| GVS:Reward:Block | 8 | 232 | 3.919349e+03 | 47187.79 | 2.408698e+00 | 0.0162671 | \* | 0.0023674 | 0.1138700 | 0.0766889 |  |
| GVS:Validity:Block | 4 | 116 | 2.733088e+02 | 26198.27 | 3.025374e-01 | 0.8757543 |  | 0.0001655 | 0.9684346 | 0.0103246 |  |
| Reward:Validity:Block | 4 | 116 | 1.212970e+02 | 25732.66 | 1.366984e-01 | 0.9684346 |  | 0.0000734 | 0.9684346 | 0.0046916 |  |
| GVS:Side:Block | 4 | 116 | 8.273514e+02 | 26054.91 | 9.208703e-01 | 0.4543737 |  | 0.0005007 | 0.6361232 | 0.0307769 |  |
| Reward:Side:Block | 4 | 116 | 1.174776e+03 | 32852.39 | 1.037017e+00 | 0.3815907 |  | 0.0007108 | 0.6361232 | 0.0345246 |  |
| Validity:Side:Block | 2 | 58 | 6.537220e+01 | 16617.86 | 1.140817e-01 | 0.8923848 |  | 0.0000396 | 0.9684346 | 0.0039184 |  |
| GVS:Reward:Validity:Side | 4 | 116 | 2.051720e+03 | 23566.13 | 2.524805e+00 | 0.0445577 | \* | 0.0012407 | 0.2079359 | 0.0800895 |  |
| GVS:Reward:Validity:Block | 8 | 232 | 6.297078e+02 | 44612.58 | 4.093358e-01 | 0.9146021 |  | 0.0003811 | 0.9684346 | 0.0139186 |  |
| GVS:Reward:Side:Block | 8 | 232 | 2.242286e+03 | 45999.03 | 1.413645e+00 | 0.1914967 |  | 0.0013558 | 0.4468256 | 0.0464806 |  |
| GVS:Validity:Side:Block | 4 | 116 | 1.206655e+02 | 18987.58 | 1.842942e-01 | 0.9461592 |  | 0.0000731 | 0.9684346 | 0.0063148 |  |
| Reward:Validity:Side:Block | 4 | 116 | 2.243450e+03 | 32332.90 | 2.012193e+00 | 0.0972983 |  | 0.0013565 | 0.3321724 | 0.0648839 |  |
| GVS:Reward:Validity:Side:Block | 8 | 232 | 1.707765e+03 | 46059.64 | 1.075240e+00 | 0.3788624 |  | 0.0010329 | 0.6361232 | 0.0357517 |  |

The GVS by Reward interaction is thus confirmed (p=0.019, p\_fdr=0.039). There was no three-way interaction.

As a reminder, here’s the graphical depiction of the two-way:

```
my_plot(ART, dv <- "response_time", withinvars = c("Reward", "GVS"), 
       individual_data = "none")
```

## Bayesian ANOVA

Our secondary analysis was based on a Bayesian ANOVA, as implemented in the `BayesFactor` package (see for example here). We will use objective prior to avoid some degree of freedom.We use a bottom-up approach which mimics our type 2 test and limits computational time.

```
DF= ART %>% 
    ddply(c("GVS", "Validity", "Reward", "Block", "Side", "Subject"), 
          summarise, dv= mean(response_time)) %>%
    mutate(Validity= as.factor(Validity)) %>%
    mutate(Side= as.factor(Side))

anovaBF(dv ~ GVS*Reward*Validity*Block*Side + Subject, DF, 
        whichRandom="Subject", whichModels= "bottom", progress= F)
```

When compared against the model 

...

Enter one or more search terms in the box to filter the models in the table. If more than one term is included, matching will be performed with `or`. Special search terms are allowed:

| Code | Function | Example | What example does |
| --- | --- | --- | --- |
| + | Require this term in search results | +shape | Requires all models to include âshapeâ |
| - | Require this term NOT to appear in search results | -shape | Requires all models to exclude âshapeâ |
| # (with number) | Return results with certain number of terms | #2 | Requires all models to have two terms |
| @ (with :, ::, â¦) | Return results containing interactions of certain size | @:: | Requires all models to have a three-way interaction |
| < or > | Limits sizes of Bayes factors | >2 | Returns models whose Bayes factor is greater than 2 |

Click on a row in the Bayes factor table to make that model the denominator. Sort by clicking on the arrows in the column headers.

|  |  |  |
| --- | --- | --- |
| ...the model below... | ...is preferred by... |  |

The two-way is not confirmed here instead, we only have convincing evidence for the three main effects of Reward, Validity, and Side. We can have a sensitivity check for the two-way, since results partially depend on the specific prior we choose:

```
sapply(seq(0.1, 1, by= 0.1), function(x)
extractBF(anovaBF(dv ~ GVS:Reward + Subject, DF, rscaleFixed= x,
                  whichRandom="Subject", whichModels= "bottom", progress= F))["GVS:Reward + Subject", "bf"])
```

```
##  [1] 3.18432916 1.37310979 0.55360380 0.24968592 0.12363663 0.06589923
##  [7] 0.03857219 0.02333978 0.01526622 0.01030670
```

The Bayes Factor was consistently below 1 with priors >= 0.3. The exceptions were for a prior scaled width of 0.1 (BF>3) or 0.2 (BF= 1.38). In my understanding it is not unusual: it peaks in correspondence of the effect size of the data (pes= ~ 0.1). It is generally quite consistent across different priors, the order of magnitude should not change much, but in this case there’s some ambiguity left…

We can do the same for the three-way GVS by Reward by Validity:

```
sapply(seq(0.1, 1, by= 0.1), function(x)
extractBF(anovaBF(dv ~ GVS:Reward:Validity + Subject, DF, rscaleFixed= x,
                  whichRandom="Subject", whichModels= "bottom", progress= F))["GVS:Validity:Reward + Subject", "bf"])
```

```
##  [1] 0.1931508313 0.0500838163 0.0168206772 0.0068245489 0.0032257959
##  [6] 0.0016706283 0.0009565629 0.0005837157 0.0003684057 0.0002505582
```

The results in this case were more consistent, and even with the narrowest prior (r= 0.1) the BF was supporting the null (BF= 0.19).

## Individual Results

How many subjects show an effect of reward that is more pronounced (steep) for the SHAM condition with respect to the Right-Anodal condition? RTs are now predicted by the number of points rewarded for each response (0, 2, 10); b coefficients are extracted for each Subject and GVS condition. We now proceed in calculating the differences between slopes. B coefficients are first multiplied by -1, which is more intuitive. Then the coefficients for Right-Anodal are subtracted from those in the SHAM condition. At this point large values indicate larger abolishment of reward sensitivity, negative values an enhancement instead.

```
slopes= ddply(ART, c("Reward", "GVS", "Subject"), 
              summarise, RT= mean(response_time)) %>%
        ddply(c("GVS", "Subject"), summarise, 
              b= lm(RT ~ c(0, 2, 10))$coefficients[2]) %>%
        with(lapply(levels(Subject), function(x){
             return((b[Subject==x & GVS=="SHAM"]*(-1)) - (b[Subject==x & GVS=="Right-Anodal"])*(-1))})) %>% 
        unlist

sum(slopes>0)
```

```
## [1] 22
```

The effect was thus there for 22 subjects over 30 (73.3%).

```
boxplot(slopes)
```

```
t.test(slopes)
```

```
## 
##  One Sample t-test
## 
## data:  slopes
## t = 3.0365, df = 29, p-value = 0.00502
## alternative hypothesis: true mean is not equal to 0
## 95 percent confidence interval:
##  0.216610 1.110461
## sample estimates:
## mean of x 
## 0.6635356
```

The 95% CI of the indices we computed do not include 0. We will use this index for correlations of interest in exploratory analyses.

## Data Distribution

We now assess data distribution with greater details. We set a response limit to 500 ms. As a result, we now have a truncated distribution. The following plot helps understanding if something is happening beyond this limit, with respect to the Reward by GVS interaction that we have described. (We also had a lower bound to 100 ms but anticipations were very uncommon).

```
p_full= ART.accuracy %>% filter(correct==1) %>% #exclude wrong responses only
        ggplot(aes(x= response_time, fill= Reward)) + 
        commonTheme + xlab("Response Time (ms)") + xlim(c(0, 800)) +
        geom_vline(xintercept= c(100, 500), linetype= "dashed", size= 1.1, color= "darkgray") +
        geom_density(alpha= 0.3) + 
        facet_wrap("GVS", nrow= 3)


p_zoom_centre= ART.accuracy %>% filter(correct==1) %>% #exclude wrong responses only
        ggplot(aes(x= response_time, fill= Reward)) + 
        commonTheme + xlab("Response Time (ms)") + xlim(c(0, 800)) +
        geom_vline(xintercept= c(100, 500), linetype= "dashed", size= 1.1, color= "darkgray") +
        geom_density(alpha= 0.3) + coord_cartesian(xlim= c(350, 400), ylim= c(0.005, 0.008))+
        facet_wrap("GVS", nrow= 3) + guides(fill= F)

p_zoom_tail= ART.accuracy %>% filter(correct==1) %>% #exclude wrong responses only
        ggplot(aes(x= response_time, fill= Reward)) + 
        commonTheme + xlab("Response Time (ms)") + xlim(c(0, 800)) +
        geom_vline(xintercept= c(100, 500), linetype= "dashed", size= 1.1, color= "darkgray") +
        geom_density(alpha= 0.3) + coord_cartesian(xlim= c(500, 650), ylim= c(0, 0.002))+
        facet_wrap("GVS", nrow= 3) + guides(fill= F)

grid.arrange(p_full, p_zoom_centre, p_zoom_tail, layout_matrix = rbind(c(1,1), c(2,3)))
```

Here we can see that high rewards are associated with: smaller latencies; higher peaks for fast RTs; smaller “long tails” of the distribution. This is fairly abolished for the Right-Anodal GVS condition (bottom). At least visually not much seems to be going on after our cut off (500 ms, gray dashed line). Specifically, a residual effect of Reward (larger density of RTs following no rewards or small rewards with respect to high rewards) seems to be present for all GVS conditions. The distribution can be described by three parameters: mu, reflecting the mean of the gaussian part of the distribution, sigma, reflecting its amplitude (or standard deviation), and tau, reflecting the exponential component of the long tail and thus “residual” effects. We can calculate the three indices with the package `retimes` and submit them to three ANOVAs. We will do it only for GVS and Reward factors (more observations yield more precise estimates).

```
DF= ART.accuracy %>% filter(correct==1) %>%
    ddply(c("GVS", "Reward", "Subject"), summarise, mu= mexgauss(response_time)[1], 
                                                    sigma= mexgauss(response_time)[2], 
                                                    tau= mexgauss(response_time)[3])
#results
#mu
with(DF, tapply(mu, list(Reward, GVS), mean))
```

|  | Left-Anodal | SHAM | Right-Anodal |
| --- | --- | --- | --- |
| None | 356.2487 | 356.2280 | 353.1263 |
| Small | 351.9080 | 354.7869 | 353.9909 |
| High | 351.7638 | 344.6082 | 350.6483 |

```
#sigma
with(DF, tapply(sigma, list(Reward, GVS), mean))
```

|  | Left-Anodal | SHAM | Right-Anodal |
| --- | --- | --- | --- |
| None | 42.88395 | 44.66523 | 43.67564 |
| Small | 39.97114 | 44.24642 | 44.33242 |
| High | 42.98603 | 42.27451 | 43.41478 |

```
#tau
with(DF, tapply(tau, list(Reward, GVS), mean))
```

|  | Left-Anodal | SHAM | Right-Anodal |
| --- | --- | --- | --- |
| None | 42.11099 | 39.32095 | 42.08372 |
| Small | 43.56226 | 37.88330 | 39.12253 |
| High | 35.19182 | 38.40608 | 37.53430 |

```
#mu
ezANOVA(DF, .(mu), wid= .(Subject), within= c("GVS", "Reward"), type= 2)
```

```
## $ANOVA
##       Effect DFn DFd          F            p p<.05          ges
## 2        GVS   2  58  0.1217468 0.8855981505       0.0008050604
## 3     Reward   2  58 10.4370612 0.0001343724     * 0.0159213159
## 4 GVS:Reward   4 116  2.6160531 0.0387085152     * 0.0097063143
## 
## $`Mauchly's Test for Sphericity`
##       Effect         W           p p<.05
## 2        GVS 0.8552253 0.111976623      
## 3     Reward 0.6541973 0.002629798     *
## 4 GVS:Reward 0.8335650 0.835458429      
## 
## $`Sphericity Corrections`
##       Effect       GGe        p[GG] p[GG]<.05       HFe        p[HF]
## 2        GVS 0.8735343 0.8594669522           0.9248924 0.8708217107
## 3     Reward 0.7430510 0.0006653983         * 0.7738463 0.0005488448
## 4 GVS:Reward 0.9181949 0.0436797442         * 1.0678569 0.0387085152
##   p[HF]<.05
## 2          
## 3         *
## 4         *
```

```
#sigma
ezANOVA(DF, .(sigma), wid= .(Subject), within= c("GVS", "Reward"), type= 2)
```

```
## $ANOVA
##       Effect DFn DFd         F         p p<.05         ges
## 2        GVS   2  58 0.5286642 0.5922045       0.005967015
## 3     Reward   2  58 0.4432447 0.6441063       0.001370816
## 4 GVS:Reward   4 116 1.3593005 0.2523990       0.007221980
## 
## $`Mauchly's Test for Sphericity`
##       Effect         W          p p<.05
## 2        GVS 0.9540365 0.51749946      
## 3     Reward 0.9426039 0.43712842      
## 4 GVS:Reward 0.5073998 0.02908939     *
## 
## $`Sphericity Corrections`
##       Effect       GGe     p[GG] p[GG]<.05       HFe     p[HF] p[HF]<.05
## 2        GVS 0.9560563 0.5843385           1.0219213 0.5922045          
## 3     Reward 0.9457194 0.6332514           1.0097025 0.6441063          
## 4 GVS:Reward 0.7438499 0.2607817           0.8382644 0.2579425
```

```
#tau
ezANOVA(DF, .(tau), wid= .(Subject), within= c("GVS", "Reward"), type= 2)
```

```
## $ANOVA
##       Effect DFn DFd         F           p p<.05         ges
## 2        GVS   2  58 0.4291027 0.653143114       0.004094088
## 3     Reward   2  58 6.8736681 0.002094325     * 0.024027791
## 4 GVS:Reward   4 116 2.0100810 0.097607620       0.020605192
## 
## $`Mauchly's Test for Sphericity`
##       Effect         W          p p<.05
## 2        GVS 0.8068438 0.04955103     *
## 3     Reward 0.8772292 0.15980243      
## 4 GVS:Reward 0.8884916 0.95405985      
## 
## $`Sphericity Corrections`
##       Effect       GGe       p[GG] p[GG]<.05       HFe       p[HF]
## 2        GVS 0.8381133 0.618291065           0.8836041 0.628716108
## 3     Reward 0.8906538 0.003153231         * 0.9449246 0.002573091
## 4 GVS:Reward 0.9431881 0.101985345           1.1018103 0.097607620
##   p[HF]<.05
## 2          
## 3         *
## 4
```

Most of the differences (GVS by Reward interaction) seem thus driven by a displacement in the mu parameter rather than tau - residual - processing. The tau parameter though appears to present indeed the reverse pattern, suggesting the time-pressure was relevant.

## Effect of Session / Counterbalancing Order

During each session, and from the first to the last one, participants on average gradually improved. We sought to check whether somehow this has a role in the effects we reported (for example being only or particularly evident for the first session, where variability was greater).

We now have to connect each GVS session with its order.

```
#ART$datetime #this variables includes session dates and time
ART$datetime[1] #e.g.
```

```
## [1] "07/10/17 10:12:59"
```

```
#initialise Session variable
ART$SessionNb= NA

#only retain the dates:
ART$day= as.Date(ART$datetime, format=  "%m/%d/%Y %H:%M:%S")

#now summarise for each subject and GVS session
tapply(ART$day, list(ART$Subject, ART$GVS), function(x) as.character(x[1]))
```

|  | Left-Anodal | SHAM | Right-Anodal |
| --- | --- | --- | --- |
| 2 | 0017-06-28 | 0017-06-14 | 0017-06-22 |
| 3 | 0017-06-14 | 0017-06-16 | 0017-06-22 |
| 4 | 0017-06-14 | 0017-06-23 | 0017-06-16 |
| 5 | 0017-06-30 | 0017-06-29 | 0017-06-28 |
| 6 | 0017-07-07 | 0017-07-21 | 0017-06-29 |
| 7 | 0017-06-29 | 0017-06-22 | 0017-06-30 |
| 8 | 0017-06-30 | 0017-06-22 | 0017-06-23 |
| 9 | 0017-07-03 | 0017-07-07 | 0017-07-12 |
| 11 | 0017-07-19 | 0017-07-10 | 0017-07-07 |
| 12 | 0017-07-12 | 0017-07-20 | 0017-07-10 |
| 13 | 0017-07-20 | 0017-07-12 | 0017-07-21 |
| 14 | 0017-07-21 | 0017-07-19 | 0017-07-20 |
| 15 | 0017-08-21 | 0017-08-23 | 0017-08-25 |
| 16 | 0017-08-23 | 0017-08-31 | 0017-08-30 |
| 17 | 0017-09-06 | 0017-08-30 | 0017-08-25 |
| 18 | 0017-08-30 | 0017-08-31 | 0017-08-28 |
| 19 | 0017-08-30 | 0017-08-28 | 0017-09-04 |
| 20 | 0017-09-07 | 0017-08-31 | 0017-09-06 |
| 21 | 0017-09-04 | 0017-09-06 | 0017-09-07 |
| 22 | 0017-09-25 | 0017-10-02 | 0017-09-27 |
| 23 | 0017-10-05 | 0017-10-04 | 0017-09-27 |
| 24 | 0017-10-04 | 0017-10-05 | 0017-09-27 |
| 25 | 0017-10-12 | 0017-10-11 | 0017-10-13 |
| 26 | 0017-10-13 | 0017-10-11 | 0017-10-12 |
| 27 | 0017-10-11 | 0017-10-13 | 0017-10-18 |
| 28 | 0017-10-16 | 0017-10-20 | 0017-10-18 |
| 29 | 0017-10-23 | 0017-10-20 | 0017-10-16 |
| 30 | 0017-10-20 | 0017-10-23 | 0017-10-18 |
| 51 | 0017-10-05 | 0017-09-27 | 0017-10-06 |
| 60 | 0017-10-02 | 0017-10-06 | 0017-10-04 |

The table above reports the dates in which we tested subjects with the relative GVS session.

```
#for every subject, check the order of sessions/days and assign value to SessionNb
for(i in levels(ART$Subject)){
  
  ordered= sort(levels(as.factor(ART$day[ART$Subject== i])))
  
  ART$SessionNb[ART$Subject== i & ART$day== ordered[1]]<- 1
  ART$SessionNb[ART$Subject== i & ART$day== ordered[2]]<- 2
  ART$SessionNb[ART$Subject== i & ART$day== ordered[3]]<- 3
  
}

#now summarise and double check with laboratory log
tapply(ART$GVS, list(ART$Subject, ART$SessionNb), function(x) as.character(x[1]))
```

|  | 1 | 2 | 3 |
| --- | --- | --- | --- |
| 2 | SHAM | Right-Anodal | Left-Anodal |
| 3 | Left-Anodal | SHAM | Right-Anodal |
| 4 | Left-Anodal | Right-Anodal | SHAM |
| 5 | Right-Anodal | SHAM | Left-Anodal |
| 6 | Right-Anodal | Left-Anodal | SHAM |
| 7 | SHAM | Left-Anodal | Right-Anodal |
| 8 | SHAM | Right-Anodal | Left-Anodal |
| 9 | Left-Anodal | SHAM | Right-Anodal |
| 11 | Right-Anodal | SHAM | Left-Anodal |
| 12 | Right-Anodal | Left-Anodal | SHAM |
| 13 | SHAM | Left-Anodal | Right-Anodal |
| 14 | SHAM | Right-Anodal | Left-Anodal |
| 15 | Left-Anodal | SHAM | Right-Anodal |
| 16 | Left-Anodal | Right-Anodal | SHAM |
| 17 | Right-Anodal | SHAM | Left-Anodal |
| 18 | Right-Anodal | Left-Anodal | SHAM |
| 19 | SHAM | Left-Anodal | Right-Anodal |
| 20 | SHAM | Right-Anodal | Left-Anodal |
| 21 | Left-Anodal | SHAM | Right-Anodal |
| 22 | Left-Anodal | Right-Anodal | SHAM |
| 23 | Right-Anodal | SHAM | Left-Anodal |
| 24 | Right-Anodal | Left-Anodal | SHAM |
| 25 | SHAM | Left-Anodal | Right-Anodal |
| 26 | SHAM | Right-Anodal | Left-Anodal |
| 27 | Left-Anodal | SHAM | Right-Anodal |
| 28 | Left-Anodal | Right-Anodal | SHAM |
| 29 | Right-Anodal | SHAM | Left-Anodal |
| 30 | Right-Anodal | Left-Anodal | SHAM |
| 51 | SHAM | Left-Anodal | Right-Anodal |
| 60 | Left-Anodal | Right-Anodal | SHAM |

The table above reports the Order of GVS sessions for each subject. You can spot a pattern (e.g. for the first column, i.e. session 1) which is only interrupted by drop outs (i.e. subjects 1 and 10). It matches the counterbalancing strategy that we adopted.

The small, gradual increase in accuracy is also mirrored by reaction times, gradually faster across sessions:

```
with(ART, tapply(response_time, list(SessionNb, Subject), mean)) %>% rowMeans()
```

```
##        1        2        3 
## 386.9252 382.5694 380.3050
```

Now we create a between-subject factor that refers to the specific counterbalancing order of GVS sessions.

```
ART$GVS_Order= NA

for(i in levels(ART$Subject)){

  ART$GVS_Order[ART$Subject== i]=
  paste(ART$GVS[ART$Subject== i & ART$SessionNb== 1][1],
        ART$GVS[ART$Subject== i & ART$SessionNb== 2][1],
        ART$GVS[ART$Subject== i & ART$SessionNb== 3][1])
  
}  

ART$GVS_Order= as.factor(ART$GVS_Order)

levels(ART$GVS_Order)
```

```
## [1] "Left-Anodal Right-Anodal SHAM" "Left-Anodal SHAM Right-Anodal"
## [3] "Right-Anodal Left-Anodal SHAM" "Right-Anodal SHAM Left-Anodal"
## [5] "SHAM Left-Anodal Right-Anodal" "SHAM Right-Anodal Left-Anodal"
```

We can now summarise data by collapsing everything in a dataframe, then run ANOVAs. Of course now GVS\_Order is a between-subject variable that leaves only a few participants per cell, so not very powerful…

```
ART %>% 
  mutate(GVS_Order= factor(GVS_Order)) %>%
  ddply(c("GVS", "GVS_Order", "Reward", "Subject"), 
        summarise, dv= mean(response_time)) %>%
  ezANOVA(dv= .(dv), wid = .(Subject), within = c("GVS", "Reward"), between = .(GVS_Order), 
          detailed=T, type = 2)
```

```
## $ANOVA
##                 Effect DFn DFd          SSn       SSd            F
## 1          (Intercept)   1  24 3.967485e+07 37657.693 2.528557e+04
## 2            GVS_Order   5  24 1.207264e+04 37657.693 1.538827e+00
## 3                  GVS   2  48 3.125049e+02  9019.739 8.315227e-01
## 5               Reward   2  48 2.821254e+03  2101.406 3.222133e+01
## 4        GVS_Order:GVS  10  48 2.635796e+03  9019.739 1.402681e+00
## 6     GVS_Order:Reward  10  48 2.538851e+02  2101.406 5.799206e-01
## 7           GVS:Reward   4  96 3.746108e+02  3023.324 2.973767e+00
## 8 GVS_Order:GVS:Reward  20  96 5.541377e+02  3023.324 8.797805e-01
##              p p<.05         ges
## 1 8.523519e-38     * 0.998696035
## 2 2.153563e-01       0.189004695
## 3 4.415596e-01       0.005996487
## 5 1.341046e-09     * 0.051649164
## 4 2.079395e-01       0.048418345
## 6 8.221152e-01       0.004877149
## 7 2.310578e-02     * 0.007179647
## 8 6.122005e-01       0.010583975
## 
## $`Mauchly's Test for Sphericity`
##                 Effect         W          p p<.05
## 3                  GVS 0.9290687 0.42909172      
## 4        GVS_Order:GVS 0.9290687 0.42909172      
## 5               Reward 0.7795250 0.05702238      
## 6     GVS_Order:Reward 0.7795250 0.05702238      
## 7           GVS:Reward 0.6502134 0.38095807      
## 8 GVS_Order:GVS:Reward 0.6502134 0.38095807      
## 
## $`Sphericity Corrections`
##                 Effect       GGe        p[GG] p[GG]<.05       HFe
## 3                  GVS 0.9337667 4.347910e-01           1.0095652
## 4        GVS_Order:GVS 0.9337667 2.138618e-01           1.0095652
## 5               Reward 0.8193531 3.004341e-08         * 0.8713193
## 6     GVS_Order:Reward 0.8193531 7.915819e-01           0.8713193
## 7           GVS:Reward 0.8428075 3.117597e-02         * 0.9971602
## 8 GVS_Order:GVS:Reward 0.8428075 5.980285e-01           0.9971602
##          p[HF] p[HF]<.05
## 3 4.415596e-01          
## 4 2.079395e-01          
## 5 1.226864e-08         *
## 6 8.010671e-01          
## 7 2.323044e-02         *
## 8 6.119598e-01
```

We confirmed the effects of Reward and GVS by Reward. The GVS\_Order had no effect, nor alone nor in interaction. It’s true that this factor has 6 levels, which leaves a total of 5 subjects per cell…

We can plot the two-way separately for SessionNb (for each session GVS will be a between groups variable):

The first session:

```
my_plot(ART[ART$SessionNb== 1,], dv <- "response_time", withinvars = "Reward", betweenvars= "GVS")
```

The effect of Reward appears large in SHAM, but very less so in both GVS conditions (especially Right-Anodal). The second session:

```
my_plot(ART[ART$SessionNb== 2,], dv <- "response_time", withinvars = "Reward", betweenvars= "GVS")
```

Presented less clear differences between conditions. Finally, the last session:

```
my_plot(ART[ART$SessionNb== 3,], dv <- "response_time", withinvars = "Reward", betweenvars= "GVS")
```

Present a pattern more similar to the first one: the effect of Reward seems reduced in both GVS conditions, Right-Anodal in first place.

To summarise: at least visually, the decrease in the effect of Reward appears there for the first and last sessions, less so for the second one (just remember that the three facets in each graph depict three different *groups* of subjects in this case)… This would speak against novelty/practice effects to drive entirely the observed results.

# Exploratory analyses

After collecting encouraging and converging statistical evidence for the two-way GVS by Reward interaction, as opposed to the three-way, we can cope with other questions. This part is completely data-driven, be warned.

## Side of the **cue**

It is possible that the Side of the *cue*, instead of that of the *target*, had an important role. We ran a further ANOVA with Cue\_Side instead of Target\_side.

We need to code it first:

```
ART$Cue_Side= NA

ART$Cue_Side= ifelse(ART$Validity== "Valid", ART$Side, 
                     ifelse(ART$Side== "Left", "Right", "Left"))

#rename
ART$Cue_Side= ifelse(ART$Cue_Side== "Left", "Left Cue", "Right Cue")

table(ART$Side, ART$Cue_Side, ART$Validity)
```

| Var1 | Var2 | Var3 | Freq |
| --- | --- | --- | --- |
| Left | Left Cue | Invalid | 0 |
|  |  | Valid | 8553 |
|  | Right Cue | Invalid | 8624 |
|  |  | Valid | 0 |
| Right | Left Cue | Invalid | 8694 |
|  |  | Valid | 0 |
|  | Right Cue | Invalid | 0 |
|  |  | Valid | 8718 |

We can now compute the ANOVA.

```
EZ= ddply(ART, c("GVS", "Validity", "Reward", "Block", "Cue_Side", "Subject"), 
          summarise, dv= mean(response_time)) %>%
    ezANOVA(dv= .(dv), wid = .(Subject), within = c("GVS", "Validity", "Reward", "Block", "Cue_Side"), 
          detailed=T, type = 2)
```

```
## Warning: Converting "Validity" to factor for ANOVA.
```

```
## Warning: Converting "Cue_Side" to factor for ANOVA.
```

We apply Greenhouse-Geisser correction where necessary and then correct for multiple tested factors (similar to what was preregistered).

```
#tests needing sphericity corrections:
EZ$`Mauchly's Test for Sphericity`$Effect[EZ$`Mauchly's Test for Sphericity`$p <0.05]
```

```
## [1] "Reward"                         "GVS:Block"                     
## [3] "GVS:Reward:Block:Cue_Side"      "Validity:Reward:Block:Cue_Side"
```

```
#apply Greenhouse-Geisser correction if necessary
EZ$ANOVA$p[EZ$ANOVA$Effect %in% EZ$`Mauchly's Test for Sphericity`$Effect[EZ$`Mauchly's Test for Sphericity`$p <0.05]] <-
EZ$`Sphericity Corrections`$`p[GG]`[EZ$`Sphericity Corrections`$Effect %in% EZ$`Mauchly's Test for Sphericity`$Effect[EZ$`Mauchly's Test for Sphericity`$p <0.05]]

#simplify
EZ= EZ$ANOVA

#corrections for multiple testing
interesting_tests= c("Reward", "GVS:Reward", "Validity:Reward", "GVS:Validity:Reward")

EZ$p_fdr= NA

#family of tests of interest 
EZ[EZ$Effect %in% interesting_tests, ]$p_fdr=
p.adjust(EZ[EZ$Effect %in% interesting_tests, ]$p, "fdr")

#second family of tests
EZ[!EZ$Effect %in% interesting_tests, ]$p_fdr=
p.adjust(EZ[!EZ$Effect %in% interesting_tests, ]$p, "fdr")
```

The `ez` package by default returns the generalized eta squared. Because more established we switch to the partial eta squared.

```
EZ$pes= EZ$SSn/(EZ$SSn + EZ$SSd)
```

Keeping in mind that our alpha threshold was set to 0.043 due to multiple longitudinal data peeks, here’s the results:

```
EZ$p_fdr_sig= ifelse(EZ$p_fdr<0.043, "*", "")
EZ
```

| Effect | DFn | DFd | SSn | SSd | F | p | p<.05 | ges | p\_fdr | pes | p\_fdr\_sig |
| --- | --- | --- | --- | --- | --- | --- | --- | --- | --- | --- | --- |
| (Intercept) | 1 | 29 | 4.762113e+08 | 594620.44 | 2.322512e+04 | 0.0000000 | \* | 0.9965437 | 0.0000000 | 0.9987529 | \* |
| GVS | 2 | 58 | 4.250960e+03 | 140258.74 | 8.789316e-01 | 0.4206831 |  | 0.0025672 | 0.6361232 | 0.0294164 |  |
| Validity | 1 | 29 | 1.849090e+04 | 52013.06 | 1.030964e+01 | 0.0032276 | \* | 0.0110717 | 0.0380368 | 0.2622675 | \* |
| Reward | 2 | 58 | 3.395922e+04 | 29072.05 | 3.387505e+01 | 0.0000000 | \* | 0.0201470 | 0.0000000 | 0.5387678 | \* |
| Block | 2 | 58 | 2.452040e+03 | 55498.32 | 1.281285e+00 | 0.2854218 |  | 0.0014824 | 0.5708436 | 0.0423128 |  |
| Cue\_Side | 1 | 29 | 1.395363e+03 | 17929.69 | 2.256902e+00 | 0.1438319 |  | 0.0008441 | 0.3661174 | 0.0722049 |  |
| GVS:Validity | 2 | 58 | 4.583726e+02 | 23056.33 | 5.765360e-01 | 0.5650280 |  | 0.0002775 | 0.7533707 | 0.0194930 |  |
| GVS:Reward | 4 | 116 | 4.496883e+03 | 42647.03 | 3.057882e+00 | 0.0194956 | \* | 0.0027153 | 0.0389912 | 0.0953863 | \* |
| Validity:Reward | 2 | 58 | 7.272197e+02 | 16703.55 | 1.262568e+00 | 0.2905857 |  | 0.0004401 | 0.3147213 | 0.0417205 |  |
| GVS:Block | 4 | 116 | 5.711355e+03 | 80464.28 | 2.058420e+00 | 0.1067697 |  | 0.0034461 | 0.3321724 | 0.0662757 |  |
| Validity:Block | 2 | 58 | 2.023590e+03 | 19677.22 | 2.982338e+00 | 0.0584986 |  | 0.0012237 | 0.2339946 | 0.0932495 |  |
| Reward:Block | 4 | 116 | 2.492950e+03 | 28385.08 | 2.546956e+00 | 0.0430628 | \* | 0.0015071 | 0.2079359 | 0.0807354 |  |
| GVS:Cue\_Side | 2 | 58 | 4.315930e+02 | 13897.00 | 9.006401e-01 | 0.4119152 |  | 0.0002612 | 0.6361232 | 0.0301211 |  |
| Validity:Cue\_Side | 1 | 29 | 1.185474e+04 | 35334.74 | 9.729450e+00 | 0.0040754 | \* | 0.0071265 | 0.0380368 | 0.2512158 | \* |
| Reward:Cue\_Side | 2 | 58 | 1.168359e+03 | 16056.72 | 2.110170e+00 | 0.1304286 |  | 0.0007069 | 0.3652000 | 0.0678290 |  |
| Block:Cue\_Side | 2 | 58 | 6.537220e+01 | 16617.86 | 1.140817e-01 | 0.8923848 |  | 0.0000396 | 0.9684346 | 0.0039184 |  |
| GVS:Validity:Reward | 4 | 116 | 7.809296e+02 | 18875.69 | 1.199795e+00 | 0.3147213 |  | 0.0004726 | 0.3147213 | 0.0397286 |  |
| GVS:Validity:Block | 4 | 116 | 2.733088e+02 | 26198.27 | 3.025374e-01 | 0.8757543 |  | 0.0001655 | 0.9684346 | 0.0103246 |  |
| GVS:Reward:Block | 8 | 232 | 3.919349e+03 | 47187.79 | 2.408698e+00 | 0.0162671 | \* | 0.0023674 | 0.1138700 | 0.0766889 |  |
| Validity:Reward:Block | 4 | 116 | 1.212970e+02 | 25732.66 | 1.366984e-01 | 0.9684346 |  | 0.0000734 | 0.9684346 | 0.0046916 |  |
| GVS:Validity:Cue\_Side | 2 | 58 | 9.443915e+02 | 18473.66 | 1.482508e+00 | 0.2355438 |  | 0.0005715 | 0.5073251 | 0.0486347 |  |
| GVS:Reward:Cue\_Side | 4 | 116 | 2.051720e+03 | 23566.13 | 2.524805e+00 | 0.0445577 | \* | 0.0012407 | 0.2079359 | 0.0800895 |  |
| Validity:Reward:Cue\_Side | 2 | 58 | 2.377573e+02 | 21682.52 | 3.179963e-01 | 0.7288659 |  | 0.0001439 | 0.9276475 | 0.0108465 |  |
| GVS:Block:Cue\_Side | 4 | 116 | 1.206655e+02 | 18987.58 | 1.842942e-01 | 0.9461592 |  | 0.0000731 | 0.9684346 | 0.0063148 |  |
| Validity:Block:Cue\_Side | 2 | 58 | 5.205320e+01 | 15658.45 | 9.640430e-02 | 0.9082420 |  | 0.0000315 | 0.9684346 | 0.0033133 |  |
| Reward:Block:Cue\_Side | 4 | 116 | 2.243450e+03 | 32332.90 | 2.012193e+00 | 0.0972983 |  | 0.0013565 | 0.3321724 | 0.0648839 |  |
| GVS:Validity:Reward:Block | 8 | 232 | 6.297078e+02 | 44612.58 | 4.093358e-01 | 0.9146021 |  | 0.0003811 | 0.9684346 | 0.0139186 |  |
| GVS:Validity:Reward:Cue\_Side | 4 | 116 | 8.196145e+02 | 25109.10 | 9.466218e-01 | 0.4397611 |  | 0.0004960 | 0.6361232 | 0.0316103 |  |
| GVS:Validity:Block:Cue\_Side | 4 | 116 | 8.273514e+02 | 26054.91 | 9.208703e-01 | 0.4543737 |  | 0.0005007 | 0.6361232 | 0.0307769 |  |
| GVS:Reward:Block:Cue\_Side | 8 | 232 | 1.707765e+03 | 46059.64 | 1.075240e+00 | 0.3788624 |  | 0.0010329 | 0.6361232 | 0.0357517 |  |
| Validity:Reward:Block:Cue\_Side | 4 | 116 | 1.174776e+03 | 32852.39 | 1.037017e+00 | 0.3815907 |  | 0.0007108 | 0.6361232 | 0.0345246 |  |
| GVS:Validity:Reward:Block:Cue\_Side | 8 | 232 | 2.242286e+03 | 45999.03 | 1.413645e+00 | 0.1914967 |  | 0.0013558 | 0.4468256 | 0.0464806 |  |

Results are similar, though Validity and Cue\_Side now interact. Let’s depict it:

```
my_plot(ART, dv <- "response_time", withinvars = c("Validity", "Cue_Side"), 
       individual_data = "none")
```

```
## [1] "Validity is involved: plotting deltas"
```

```
## Automatically converting the following non-factors to factors: Cue_Side
```

The IOR is stronger following left-sided cues. I wonder whether this is related to the main effect of target Side (faster RTs for right-sided targets). Here’s the values:

```
with(ART, tapply(response_time, list(Validity, Cue_Side), mean))
```

|  | Left Cue | Right Cue |
| --- | --- | --- |
| Invalid | 378.1478 | 383.1535 |
| Valid | 386.4758 | 383.8956 |

There is an interaction of Cue\_side with GVS and Reward, *at the uncorrected level*:

```
my_plot(ART, dv <- "response_time", withinvars = c("Reward", "Cue_Side", "GVS"), 
       individual_data = "none")
```

```
## Automatically converting the following non-factors to factors: Cue_Side
```

It seems that the reduction of reward-related boost in performance is more evident in Right-Anodal GVS when cues appear on the Left side…

We promised a Bayesian counterpart:

```
DF= ART %>% 
  ddply(c("GVS", "Validity", "Reward", "Block", "Cue_Side", "Subject"), 
        summarise, dv= mean(response_time)) %>%
  mutate(Validity= as.factor(Validity)) %>%
  mutate(Cue_Side= as.factor(Cue_Side))

sapply(seq(0.1, 1, by= 0.1), function(x)
extractBF(anovaBF(dv ~ GVS:Reward:Cue_Side + Subject, DF, rscaleFixed= x,
                  whichRandom="Subject", whichModels= "bottom", progress= F))["GVS:Reward:Cue_Side + Subject", "bf"])
```

```
##  [1] 0.481549420 0.178695393 0.072420227 0.032553075 0.016183058
##  [6] 0.008808577 0.005076783 0.003151238 0.002043715 0.001374324
```

The BF remains consistently below 1…

## Correlations

We reported that the effect of Reward is less pronounced for Right-Anodal stimulation with respect to the SHAM. The first question thus concerns a possible correlation between this and the effectiveness of GVS measured with an established perceptual task (i.e. SVV). We load and pre-process SVV data as in the relative file (**please refer to S1: SVV file for detailed information**).

```
#declare
SVV$Subject= as.factor(SVV$Subject)
SVV$GVS= factor(SVV$GVS, labels = c("SHAM", "Left-Anodal", "Right-Anodal"))
SVV$GVS= relevel(SVV$GVS, "Left-Anodal")

#drop subjects
#subject 1 to replace for a bug in the ART script, my fault, :(
SVV= SVV[!SVV$Subject== 1, ] #replaced by subject 51

#subject 10 didn't show up after first session, >:(
SVV= SVV[!SVV$Subject== 10, ] #replaced by subject 60

SVV$Subject= factor(SVV$Subject) 

#trim
#create a variables containing normalized values, for each subject
SVV$scaledFO= 0
for (i in levels(SVV$Subject)) {
  SVV$scaledFO[SVV$Subject== i]= scale(SVV$FinalOrientation[SVV$Subject== i])
}

#outlier if more than 2.5 away from the mean
SVV$Outlier= ifelse(abs(SVV$scaledFO)>2.5, 1, 0)

SVV= SVV[SVV$Outlier==0,]
```

SVV data are summarised in the companion file. Here we store the difference between SVV orientation between the Right-Anodal condition and the SHAM:

```
x= with(SVV, tapply(FinalOrientation, list(subject_nr, GVS), mean))
x= x[,3]- x[,2]

DF.correlations= data.frame(SVV_effects= x)
```

Larger values ideally indicate a larger perceptual GVS effect.

Now we summarise the effect of Reward, mostly seen for the maximum Reward amount against the null reward condition. Please refer to the relative Supplementary file to access all the details and information.

Calculating the slope of Reward for each Subject and GVS should account for the small Reward as well.

```
y= ddply(ART, c("Reward", "GVS", "Subject"), 
         summarise, RT= mean(response_time)) %>%
   ddply(c("GVS", "Subject"), 
         summarise, b= lm(RT ~ c(0, 2, 10))$coefficients[2])
```

RTs are now predicted by the number of points rewarded for each response (0, 2, 10); b coefficients are extracted for each Subject and GVS condition. We now proceed in calculating the differences between slopes. B coefficients are first multiplied by -1, which is more intuitive. Then the coefficients for Right-Anodal are subtracted from those in the SHAM condition. At this point large values indicate larger abolishment of reward sensitivity, small values an enhancement instead. (We did this before when computing the variable `slopes`).

```
#same as raw difference
#calculates delta/abolishment
y= unlist(lapply(levels(y$Subject), function(x){
  return((y$b[y$Subject==x & y$GVS=="SHAM"]*(-1)) - (y$b[y$Subject==x & y$GVS=="Right-Anodal"])*(-1))
}))
```

The number of subjects showing a slope that was more negative (steep) for SHAM with respect to Right-Anodal (pointing to enhanced sensitivity to Reward) is, as seen before:

```
sum(y>0)
```

```
## [1] 22
```

We add the slopes to our data frame:

```
DF.correlations= data.frame(DF.correlations, GVS_Reward= y)
```

Third question: is the decrease in sensitivity to rewards related to the decrease of attentional capture (as shown by the tendency for inhibition of return to be enhanced with large rewards in SHAM but less in right-Anodal condition)?

Similarly to what we did before, we now compute the slopes for the validity costs (deltas) for each Subject and GVS condition.

```
#possibly a bit unelegant... Sorry...
x= {}
for(i in levels(ART$Subject)){

 foo= ART[ART$Subject==i,] %>% with(tapply(response_time, list(Reward, Validity, GVS), mean))  
 foo= foo[, 1,]- foo[, 2,] 
 foo= apply(foo, 2, function(d) lm(d ~ c(0, 2, 10))$coefficients[2])
 
 #we now have coefficients for each GVS condition
 foo= foo[2]*(-1) - foo[3]*(-1)
 names(foo)= NULL
 x= c(x, foo)
}
```

X now indexes the amount of *abolishment* of attentional capture by high rewards due to the Right-Anodal condition as compared to SHAM. It seems less consistent than the effects of GVS on Reward, because shown by:

```
sum(x>0)
```

```
## [1] 18
```

Subjects (60%). A few extra checks:

```
boxplot(x)
```

```
t.test(x)
```

```
## 
##  One Sample t-test
## 
## data:  x
## t = 1.1891, df = 29, p-value = 0.244
## alternative hypothesis: true mean is not equal to 0
## 95 percent confidence interval:
##  -0.2303410  0.8701817
## sample estimates:
## mean of x 
## 0.3199204
```

And finally we add these slopes to our data frame:

```
DF.correlations= data.frame(DF.correlations, GVS_Validity_Reward= x)
```

Now we want to check whether unspecific effects, such as faster/slower reaction times in one condition, have a role (perhaps faster RTs, for example, are more close to the floor and hamper cognitive effects).

```
y= with(ART, tapply(response_time, list(Subject, GVS), mean))
y= y[,3]- y[,2]        

#add to data frame
DF.correlations= data.frame(DF.correlations, GVS= y)
```

Positive values indicate that Right-Anodal condition was faster than SHAM, the opposite for negative values.

Finally, we check for correlations with subject’s illusions of body rotation, which was the only item proven (barely) significant (**please refer to the relative supplementary file, S2, for detailed information about questionnaires**).

```
#PREPROCESSING
FraToEngQuestions= list("I felt my body moving", "I felt my body moving",
                "I felt my body rotating", "I felt my head leaning",
                "I felt the visual scene moving", "I felt vertigo",
                "I felt nausea", "I felt my body or one part of it changing size",
                "I felt itching", "I felt burning",
                "I felt uncomfortable", "I was focused on the task",
                "I felt motivated in accomplishing the task", "I felt motivated by rewards")

FraToEngQuestions= setNames(FraToEngQuestions, unique(EST$Question))

#check
names(FraToEngQuestions) %in% EST$Question
```

```
##  [1] TRUE TRUE TRUE TRUE TRUE TRUE TRUE TRUE TRUE TRUE TRUE TRUE TRUE TRUE
```

```
#now substitute
EST$Eng_Question= unlist(FraToEngQuestions[EST$Question])

#same for responses left / right
FraToEngLeft= list("Left", "Downward", "Backward", "Not at all")
FraToEngRight= list("Right", "Upward", "Forward", "Very much")

FraToEngLeft= setNames(FraToEngLeft, unique(EST$RespLeft))
FraToEngRight= setNames(FraToEngRight, unique(EST$RespRight))

EST$Eng_Left= unlist(FraToEngLeft[EST$RespLeft])
EST$Eng_Right= unlist(FraToEngRight[EST$RespRight])


#declare
EST$Subject= as.factor(EST$Subject)

if(sum(levels(as.factor(EST$GVS))== c("A", "B", "C"))==3){
EST$GVS= factor(EST$GVS, labels = c("SHAM", "Left-Anodal", "Right-Anodal"))
EST$GVS= relevel(EST$GVS, "Left-Anodal")} else (warning("levels unordered? Check"))

#subject 1 to replace for a bug in the script, my fault, :(
EST= EST[!EST$Subject== 1, ] #replaced with n. 51

#subject 10 didn't show up after first session, >:(
EST= EST[!EST$Subject== 10, ] #replaced with n. 60

EST$Subject= factor(EST$Subject)

#for the three similar questions...
EST$Quest_Nb= 0
interaction= interaction(EST$Subject, EST$GVS)

invisible(sapply(levels(interaction(EST$Subject, EST$GVS)), function(x){
  EST$Quest_Nb[interaction== x] <<- 1:15
}))

#half-length of the segment is 350 pixels
EST$X_Dev= EST$X_Resp/3.50
```

We can now summarise:

```
x= with(EST[EST$Quest_Nb==4,], tapply(X_Dev, list(Subject, GVS), mean))
x= x[,3]- x[,2]

#add to data frame
DF.correlations= data.frame(DF.correlations, GVS_Rotation= x)
```

Positive values indicate that Right-Anodal was associated with subjective feeling of clockwise body motion.

We can finally explore correlations, let’s prepare the functions:

```
#function to extract interesting information/parameters/coefficients
lm_parameters = function(y, x, data) {
    
    m= cor.test(data[,names(data)==y], data[,names(data)==x])
    
    DF= data.frame(y= data[,names(data)==y], x= data[,names(data)==x])
    
    #BFs for regressions with only one IV==BFs for correlations
    #Note that default objective priors differ from those of ANOVAs
    bf= extractBF(regressionBF(y ~ x, data = DF, progress = F))$bf
      
    (l= list(R = format(m$estimate, digits = 2),
            P = format(m$p.value, digits = 2),
            bf = format(bf, digits = 2)))
                    
  }

#adjust color according to strength of evidence
col.critic<-function(x,trasparency= 0.3, direction="large is better"){
  if (direction=="small.better")(
    col<-rgb(x,1-x,0,trasparency)
  ) else (
    col<-rgb(1-x,x,0,trasparency)
  )
  return(col)  
}

my_corplot= function(x, y, xlab, ylab, Cook_omit= T, data= DF.correlations){
  
  DF= data.frame(y= data[,names(data)==y], x= data[,names(data)==x])
    
  #in case of outliers
  if(Cook_omit) {
    DF= DF[!cooks.distance(lm(y ~ x, DF)) > 4/nrow(DF),]
    print(paste("Influential observations omitted:", nrow(DF), "Subjects included"))
    }
  
  color= col.critic(abs(as.numeric(
            lm_parameters("y", "x", DF)$R)))
      
  p= ggplot(DF, aes(x= x, y= y)) + commonTheme +
        geom_point(shape= 21, size= 4, stroke= 2, fill= color) +   
        geom_smooth(method= lm, color= "black", fill=color) +
        ggtitle(paste0("R= ", lm_parameters("y", "x", DF)$R,
                       ", p= ", lm_parameters("y", "x", DF)$P,
                       ", BF= ", lm_parameters("y", "x", DF)$bf)) +
     ylab(ylab) + xlab(xlab)

  return(p)
}
```

We associate each variable and label:

```
corr_var= list("SVV_effects"= "GVS perceptual effects (SVV, °)",
               "GVS_Reward"= "GVS effects on Reward (ms)",
               "GVS_Validity_Reward"= "GVS effects on Attentional Capture (ms)",
               "GVS"= "GVS general RTs advantage (ms)",
               "GVS_Rotation"= "GVS-induced illusion of rotation (%)")
```

Set up all possible combinations:

```
corr_comb= combn(length(colnames(DF.correlations)), 2)
```

Finally plot!

```
for (i in 1: ncol(corr_comb)){
  
  p= my_corplot(colnames(DF.correlations)[corr_comb[1, i]], 
             colnames(DF.correlations)[corr_comb[2, i]],
             corr_var[colnames(DF.correlations)[corr_comb[1, i]]][[1]],
             corr_var[colnames(DF.correlations)[corr_comb[2, i]]][[1]])

  print(p)
}
```

```
## [1] "Influential observations omitted: 27 Subjects included"
```

```
## [1] "Influential observations omitted: 30 Subjects included"
```

```
## [1] "Influential observations omitted: 27 Subjects included"
```

```
## [1] "Influential observations omitted: 28 Subjects included"
```

```
## [1] "Influential observations omitted: 29 Subjects included"
```

```
## [1] "Influential observations omitted: 27 Subjects included"
```

```
## [1] "Influential observations omitted: 28 Subjects included"
```

```
## [1] "Influential observations omitted: 29 Subjects included"
```

```
## [1] "Influential observations omitted: 27 Subjects included"
```

```
## [1] "Influential observations omitted: 26 Subjects included"
```

Thus, this is the only possible link among those many:

```
my_corplot("GVS_Reward", "GVS_Validity_Reward", "GVS effects on Reward (ms)", "GVS effects on Attentional Capture (ms)")
```

```
## [1] "Influential observations omitted: 29 Subjects included"
```

The reduction in attentional capture seems to correlate - at least visually, and to a small extent - with the reduction in sensitivity to rewards.

Note that statistics displayed on plots: 1) exclude extremely influential points; 2) are not corrected for multiple tests. This is what happens when a correction is applied (but no influent points are omitted):

```
corr.test(DF.correlations, adjust= "fdr")
```

```
## Call:corr.test(x = DF.correlations, adjust = "fdr")
## Correlation matrix 
##                     SVV_effects GVS_Reward GVS_Validity_Reward   GVS
## SVV_effects                1.00      -0.05                0.12 -0.04
## GVS_Reward                -0.05       1.00                0.28  0.08
## GVS_Validity_Reward        0.12       0.28                1.00  0.30
## GVS                       -0.04       0.08                0.30  1.00
## GVS_Rotation              -0.17      -0.02                0.15 -0.50
##                     GVS_Rotation
## SVV_effects                -0.17
## GVS_Reward                 -0.02
## GVS_Validity_Reward         0.15
## GVS                        -0.50
## GVS_Rotation                1.00
## Sample Size 
## [1] 30
## Probability values (Entries above the diagonal are adjusted for multiple tests.) 
##                     SVV_effects GVS_Reward GVS_Validity_Reward  GVS
## SVV_effects                0.00       0.93                0.90 0.93
## GVS_Reward                 0.80       0.00                0.46 0.93
## GVS_Validity_Reward        0.54       0.14                0.00 0.46
## GVS                        0.84       0.67                0.11 0.00
## GVS_Rotation               0.38       0.93                0.43 0.00
##                     GVS_Rotation
## SVV_effects                 0.87
## GVS_Reward                  0.93
## GVS_Validity_Reward         0.87
## GVS                         0.05
## GVS_Rotation                0.00
## 
##  To see confidence intervals of the correlations, print with the short=FALSE option
```

There is one significant correlation:

```
my_corplot("GVS", "GVS_Rotation", "GVS general RTs advantage (ms)", "GVS-induced illusion of rotation (%)", 
           Cook_omit=F)
```

Perhaps not surprising: those who experienced the largest GVS illusions of body rotation were generally slower with respect to the SHAM (but note that this changes dramatically when excluding four extreme observations). Data from the self-evaluation, in addition, do not appear properly normally-distributed.

```
my_corplot("GVS", "GVS_Rotation", "GVS general RTs advantage (ms)", "GVS-induced illusion of rotation (%)", 
           Cook_omit=T)
```

```
## [1] "Influential observations omitted: 26 Subjects included"
```

## Appendix

This is the function to summarise data (`summarySEwithin`):

```
## Summarizes data.
## Gives count, mean, standard deviation, standard error of the mean, and confidence interval (default 95%).
##   data: a data frame.
##   measurevar: the name of a column that contains the variable to be summariezed
##   groupvars: a vector containing names of columns that contain grouping variables
##   na.rm: a boolean that indicates whether to ignore NA's
##   conf.interval: the percent range of the confidence interval (default is 95%)
summarySE <- function(data=NULL, measurevar, groupvars=NULL, na.rm=FALSE,
                      conf.interval=.95, .drop=TRUE) {
  library(plyr)
  
  # New version of length which can handle NA's: if na.rm==T, don't count them
  length2 <- function (x, na.rm=FALSE) {
    if (na.rm) sum(!is.na(x))
    else       length(x)
  }
  
  # This does the summary. For each group's data frame, return a vector with
  # N, mean, and sd
  datac <- ddply(data, groupvars, .drop=.drop,
                 .fun = function(xx, col) {
                   c(N    = length2(xx[[col]], na.rm=na.rm),
                     mean = mean   (xx[[col]], na.rm=na.rm),
                     sd   = sd     (xx[[col]], na.rm=na.rm)
                   )
                 },
                 measurevar
  )
  
  # Rename the "mean" column    
  datac <- plyr::rename(datac, c(mean = measurevar))
  
  datac$se <- datac$sd / sqrt(datac$N)  # Calculate standard error of the mean
  
  # Confidence interval multiplier for standard error
  # Calculate t-statistic for confidence interval: 
  # e.g., if conf.interval is .95, use .975 (above/below), and use df=N-1
  ciMult <- qt(conf.interval/2 + .5, datac$N-1)
  datac$ci <- datac$se * ciMult
  
  return(datac)
}


## Norms the data within specified groups in a data frame; it normalizes each
## subject (identified by idvar) so that they have the same mean, within each group
## specified by betweenvars.
##   data: a data frame.
##   idvar: the name of a column that identifies each subject (or matched subjects)
##   measurevar: the name of a column that contains the variable to be summariezed
##   betweenvars: a vector containing names of columns that are between-subjects variables
##   na.rm: a boolean that indicates whether to ignore NA's
normDataWithin <- function(data=NULL, idvar, measurevar, betweenvars=NULL,
                           na.rm=FALSE, .drop=TRUE) {
  library(plyr)
  
  # Measure var on left, idvar + between vars on right of formula.
  data.subjMean <- ddply(data, c(idvar, betweenvars), .drop=.drop,
                         .fun = function(xx, col, na.rm) {
                           c(subjMean = mean(xx[,col], na.rm=na.rm))
                         },
                         measurevar,
                         na.rm
  )
  
  # Put the subject means with original data
  data <- merge(data, data.subjMean)
  
  # Get the normalized data in a new column
  measureNormedVar <- paste(measurevar, "_norm", sep="")
  data[,measureNormedVar] <- data[,measurevar] - data[,"subjMean"] +
    mean(data[,measurevar], na.rm=na.rm)
  
  # Remove this subject mean column
  data$subjMean <- NULL
  
  return(data)
}


## Retrieved here: http://www.cookbook-r.com/Graphs/Plotting_means_and_error_bars_(ggplot2)/
## Summarizes data, handling within-subjects variables by removing inter-subject variability.
## It will still work if there are no within-S variables.
## Gives count, un-normed mean, normed mean (with same between-group mean),
##   standard deviation, standard error of the mean, and confidence interval.
## If there are within-subject variables, calculate adjusted values using method from Morey (2008).
##   data: a data frame.
##   measurevar: the name of a column that contains the variable to be summariezed
##   betweenvars: a vector containing names of columns that are between-subjects variables
##   withinvars: a vector containing names of columns that are within-subjects variables
##   idvar: the name of a column that identifies each subject (or matched subjects)
##   na.rm: a boolean that indicates whether to ignore NA's
##   conf.interval: the percent range of the confidence interval (default is 95%)
summarySEwithin <- function(data=NULL, measurevar, betweenvars=NULL, withinvars=NULL,
                            idvar=NULL, na.rm=FALSE, conf.interval=.95, .drop=TRUE) {
  
  # Ensure that the betweenvars and withinvars are factors
  factorvars <- vapply(data[, c(betweenvars, withinvars), drop=FALSE],
                       FUN=is.factor, FUN.VALUE=logical(1))
  
  if (!all(factorvars)) {
    nonfactorvars <- names(factorvars)[!factorvars]
    message("Automatically converting the following non-factors to factors: ",
            paste(nonfactorvars, collapse = ", "))
    data[nonfactorvars] <- lapply(data[nonfactorvars], factor)
  }
  
  # Get the means from the un-normed data
  datac <- summarySE(data, measurevar, groupvars=c(betweenvars, withinvars),
                     na.rm=na.rm, conf.interval=conf.interval, .drop=.drop)
  
  # Drop all the unused columns (these will be calculated with normed data)
  datac$sd <- NULL
  datac$se <- NULL
  datac$ci <- NULL
  
  # Norm each subject's data
  ndata <- normDataWithin(data, idvar, measurevar, betweenvars, na.rm, .drop=.drop)
  
  # This is the name of the new column
  measurevar_n <- paste(measurevar, "_norm", sep="")
  
  # Collapse the normed data - now we can treat between and within vars the same
  ndatac <- summarySE(ndata, measurevar_n, groupvars=c(betweenvars, withinvars),
                      na.rm=na.rm, conf.interval=conf.interval, .drop=.drop)
  
  # Apply correction from Morey (2008) to the standard error and confidence interval
  #  Get the product of the number of conditions of within-S variables
  nWithinGroups    <- prod(vapply(ndatac[,withinvars, drop=FALSE], FUN=nlevels,
                                  FUN.VALUE=numeric(1)))
  correctionFactor <- sqrt( nWithinGroups / (nWithinGroups-1) )
  
  # Apply the correction factor
  ndatac$sd <- ndatac$sd * correctionFactor
  ndatac$se <- ndatac$se * correctionFactor
  ndatac$ci <- ndatac$ci * correctionFactor
  
  # Combine the un-normed means with the normed results
  merge(datac, ndatac)
}
```
